# Spaced Training Forms Complementary Long-Term Memories of Opposite Valence in *Drosophila*

**DOI:** 10.1016/j.neuron.2022.07.025

**Published:** 2022-08-17

**Authors:** Pedro F. Jacob, Scott Waddell

## Main text

(Neuron *106*, 977–991.e1–e4; June 17, 2020)

In the originally published version of this paper, the mean calcium traces in Figure 3H were mistakenly duplicated from Figure 3G. The corresponding area under curve quantifications were, however, correct in the respective panels of the original figure. Corrected mean calcium traces for Figure 3H are now provided in the figure below. No conclusions of the paper have changed. The authors apologize for the error.

**Figure:**
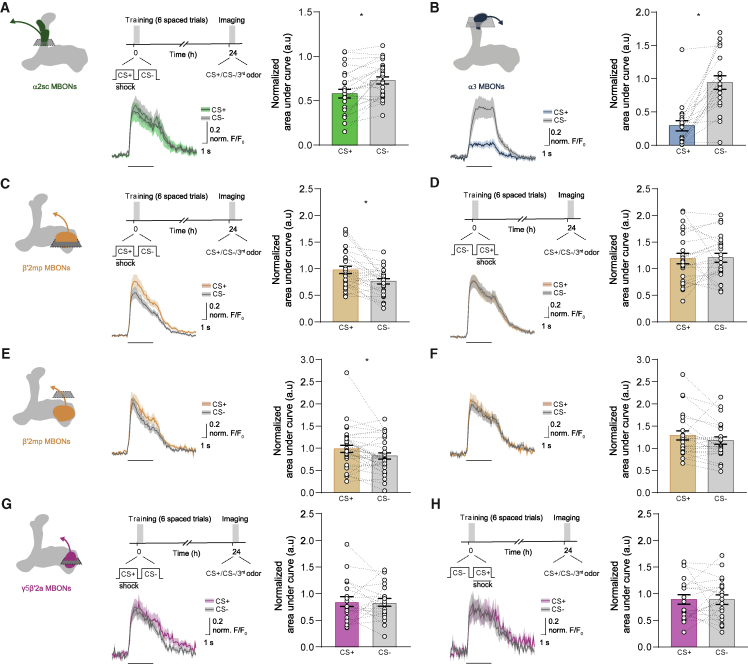
Figure 3. Parallel Aversive and Safety Memories Can Be Recorded as Depression of Odor-Specific Responses in Corresponding MBONs

