# Supplementary material for: Spaced Training Forms Complementary Long-Term Memories of Opposite Valence in Drosophila
Source: Neuron. 2020 Jun 17;106(6):977–991.e4. doi: 10.1016/j.neuron.2020.03.013 (PMC7302427; doi:10.1016/j.neuron.2020.03.013)
Supplement: Document S1. Figures S1–S6 and Tables S1 and S2 [file mmc1.pdf]

Neuron, Volume *106*

Supplemental Information

Spaced Training Forms Complementary Long-Term Memories of Opposite Valence in *Drosophila*

Pedro F. Jacob and Scott Waddell

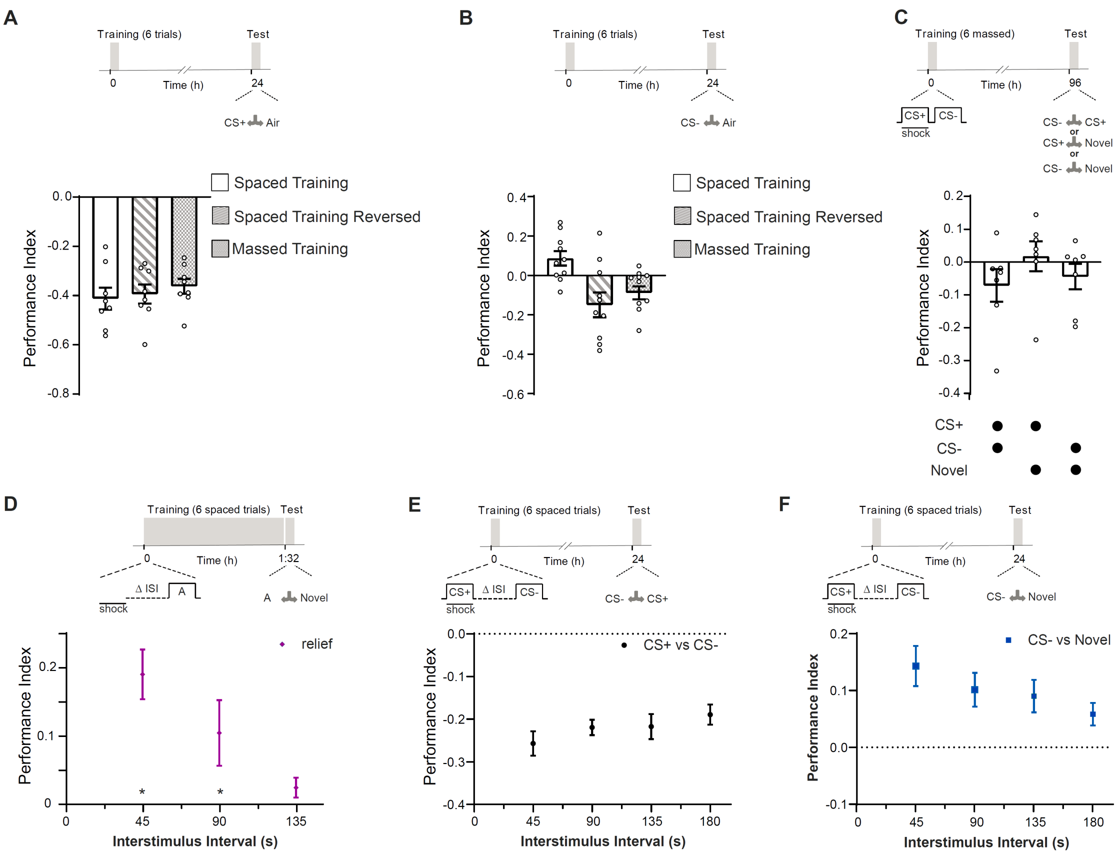

Figure S1. Related to Figure 1.

Spaced training forms CS+ aversive memory and CS- approach memory.

(**A**) Spaced, reversed spaced and massed training generated a similar 24 h CS+ aversive memory when flies were tested CS+ *vs.* Air. (**B**) A 24 h CS- approach memory (CS- *vs.* Air), was only measurable following spaced training spaced. After reversed spaced and massed training flies avoided the CS- odor. (**C**) Massed training did not generate measurable 96 h memory. (**D**) Spaced relief training (6 trials, shock then odor A training, with 15 min ITI) generated approach memory to odor A, when measured immediately after training. Relief memory to odor A was not formed when ISI was increased from 45 to 135 s. Varying ISI between CS+ and CS- in spaced training produced similar 24 h LTM (**E**) and CS- approach memory (**F**). Asterisks denote significant difference. Data mean ± SEM. Individual data points displayed as dots. See Table S1 for statistics.

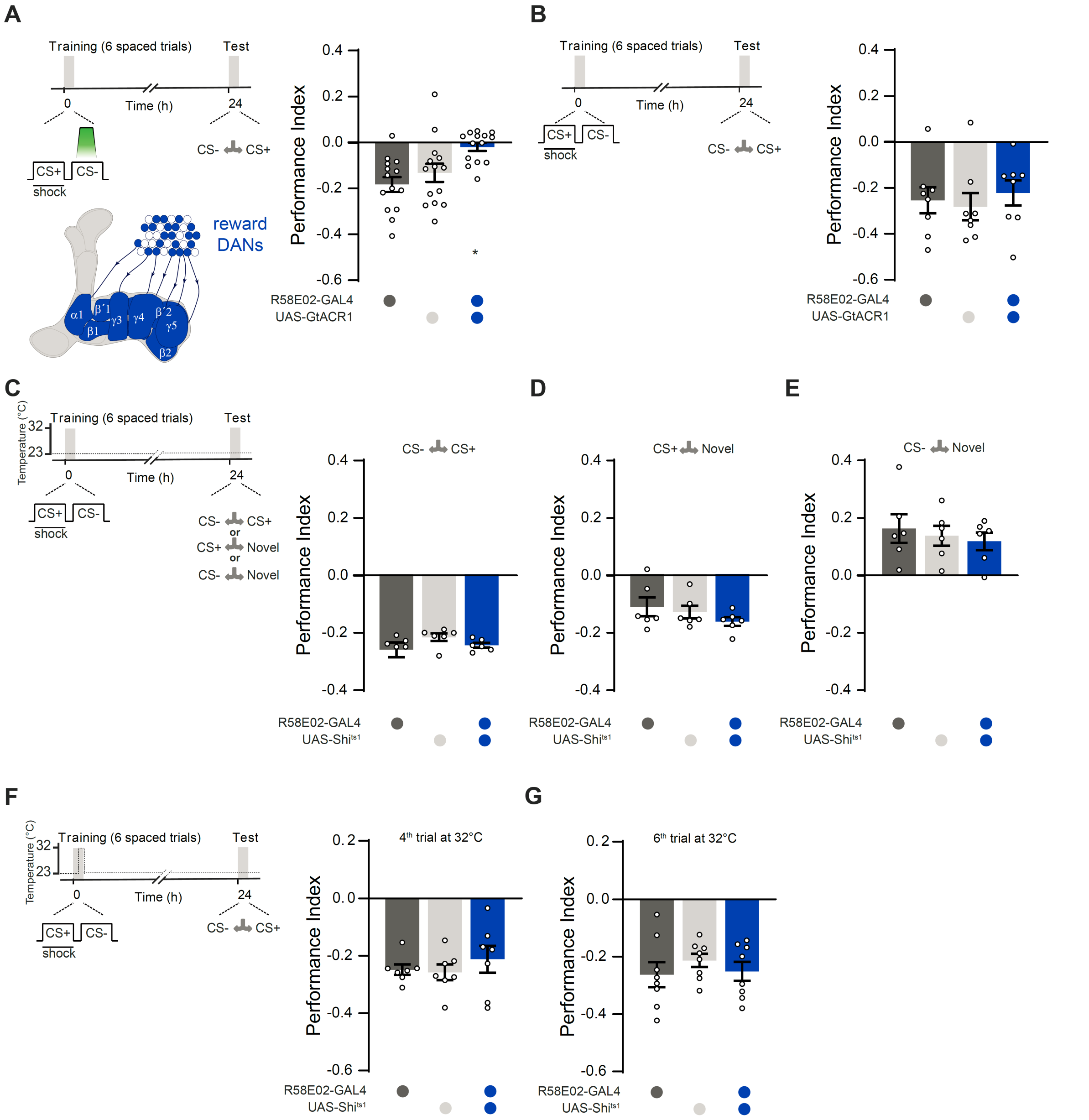

Figure S2. Related to Figure 2.

CS- approach memory requires PAM DANs during CS- presentation.

(**A**) Left: protocol with green light exposure during CS- presentation and PAM/R58E02-GAL4 DAN schematic. Right: Blocking PAM DANs with R58E02-GAL4; UAS-GtACR1 during CS-impaired 24 h memory. (**B**) Left: protocol without green light. Right: UAS-GtACR1 expression in PAM DANs (R58E02-GAL4) does not disrupt 24 h memory after spaced training. (**C**) Left: protocol. Right: Expressing UAS-*Shi*^{ts1} in R58E02-GAL4 DANs does not disrupt 24 h memory after spaced training at permissive 23˚C. (**D**) CS+ aversive memory is unaffected. (**E**) CS- approach memory is unaffected. (**F**) Left: protocol with temperature shifting, dashed line. Right: Blocking DANs with R58E02-GAL4; UAS-*Shi*^{ts1}, only during the 4th trial or (**G**) only the 6th trial did not alter 24 h memory. Asterisks denote significant differences. Data mean ± SEM. Individual data points displayed as dots. See Table S1 for statistics.

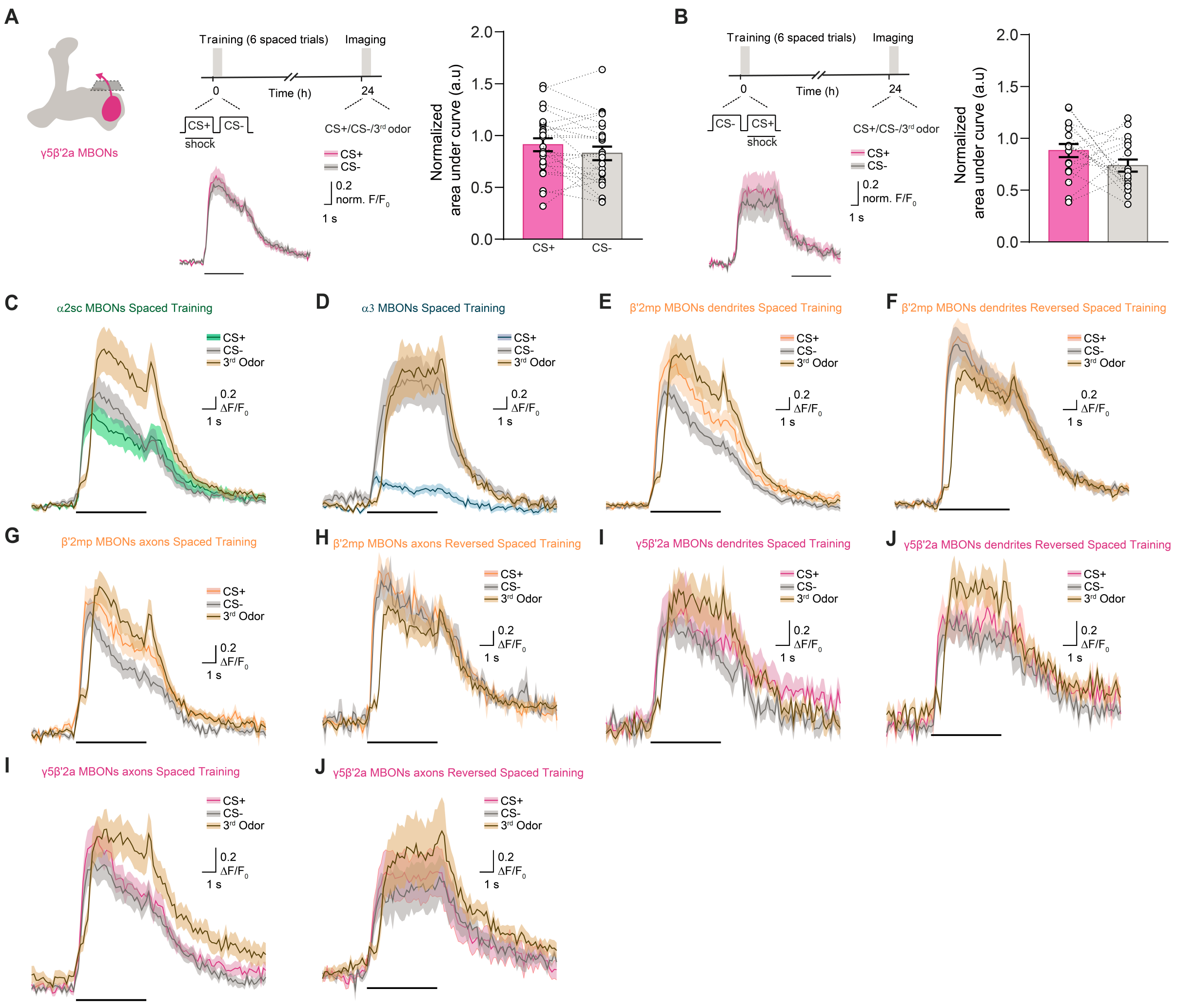

Figure S3. Related to Figure 3.

Parallel aversive and safety memories can be recorded as depression of odor-specific responses in corresponding MBONs.

Odor responses in MBON-γ5β′2a are unchanged following spaced training. Imaging plane in presynaptic terminals of MBON-γ5β′2a and training and imaging protocols. (**A**) Spaced training and (**B**) reversed spaced training do not change odor-evoked responses in MBON-γ5β′2a. (**C-J**) Non-normalized imaging traces for the MBON recordings reported in Figure 3 and S3A and B, in the order depicted in each figure. Odor-evoked activity traces show mean (solid line) with SEM (shadow). Black line underneath, 5 s odor. Asterisks denote significant difference between averaged CS+ and CS- responses. See Table S1 for statistics.

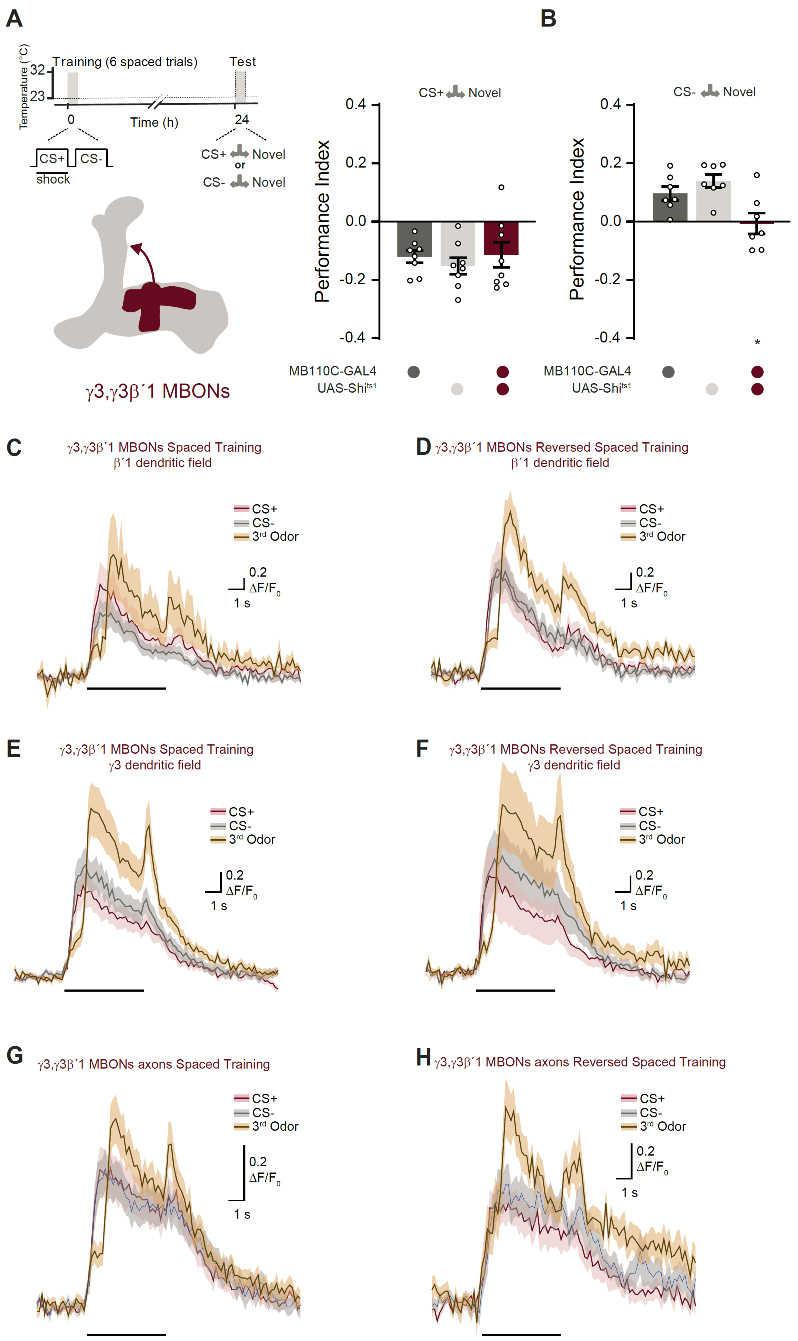

Figure S4. Related to Figure 4.

Output from γ3, γ3β′1 MBONs is required for expression of CS- approach memory.

(**A**) Left: protocol with temperature shifting (dashed line) and illustration of γ3, γ3β′1 MBONs. Right: blocking γ3, γ3β′1 MBONs during testing with MB110C-GAL4/UAS-*Shi*^{ts1} did not impair CS+ aversive memory (CS+ *vs*. Novel). (**B**) Blocking γ3, γ3β′1 MBONs during testing impaired CS- approach memory. (**C-H**) Non-normalized imaging traces for the recordings of γ3, γ3β′1 MBON responses reported in Figure 4, in the order they are depicted in the figure. Odor-evoked activity traces show mean (solid line) with SEM (shadow). Black line underneath, 5 s odor. Asterisks denote significant difference. Data mean ± SEM. Individual data points displayed as dots. See Table S1 for statistics.

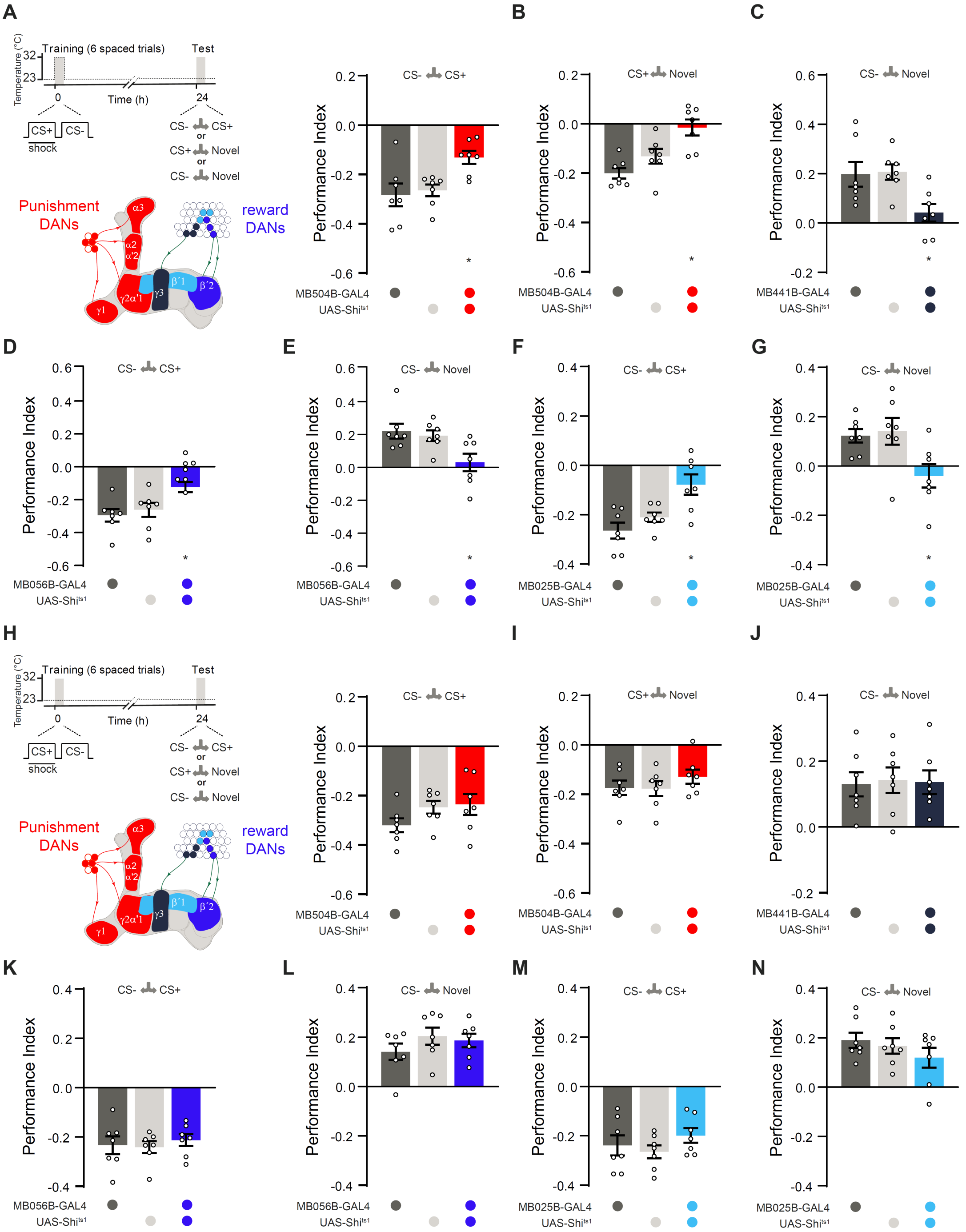

Figure S5. Related to Figure 5.

Blocking specific dopaminergic neurons during spaced training localises discrete sites of aversive and safety memory.

GAL4 driver and temperature control experiments for data presented in Figure 5. (**A**) Left: protocol with temperature shifting (dashed line) and color-coded illustration of DANs labelled with each GAL4. Right: blocking PPL1-DANs during spaced training with MB504B-GAL4; UAS-*Shi*^{ts1} impaired 24 h memory and (**B**) CS+ aversive memory (CS+ *vs*. Novel). (**C**) CS- approach memory was impaired with PAM-γ3 block. (**D**) Blocking PAM-β′2mp impaired 24 h memory and (**E**) CS- approach memory. (**F**) Blocking PAM-β′1 impaired 24 h memory and

(**G**) CS- approach memory. (**H**) Left: protocol (dashed line) and color-coded illustration of DANs labelled with each GAL4. Right: Expressing UAS-*Shi*^{ts1} in PPL1-DANs (MB504B-GAL4) did not disrupt 24 h memory after spaced training at permissive 23˚C. (**I**) CS+ aversive memory was also unaffected. (**J**) CS- approach memory was unaffected for MB441B-GAL4; UAS-*Shi*^{ts1}(PAM-γ3) flies at permissive 23˚C. (**K**) At permissive 23˚C, 24 h memory of MB056B-GAL4; UAS-*Shi*^{ts1} (PAM-β′2mp) flies was unaffected, as was (**L**) MB056B-GAL4; UAS-*Shi*^{ts1} CS- approach memory, (**M**) MB025B-GAL4; UAS-*Shi*^{ts1} (PAM-β′1) 24 h memory, and (**N**) MB025B-GAL4; UAS-*Shi*^{ts1} CS- approach memory. Asterisks denote significant differences. Data mean ± SEM. Individual data points displayed as dots. See Table S1 for statistics.

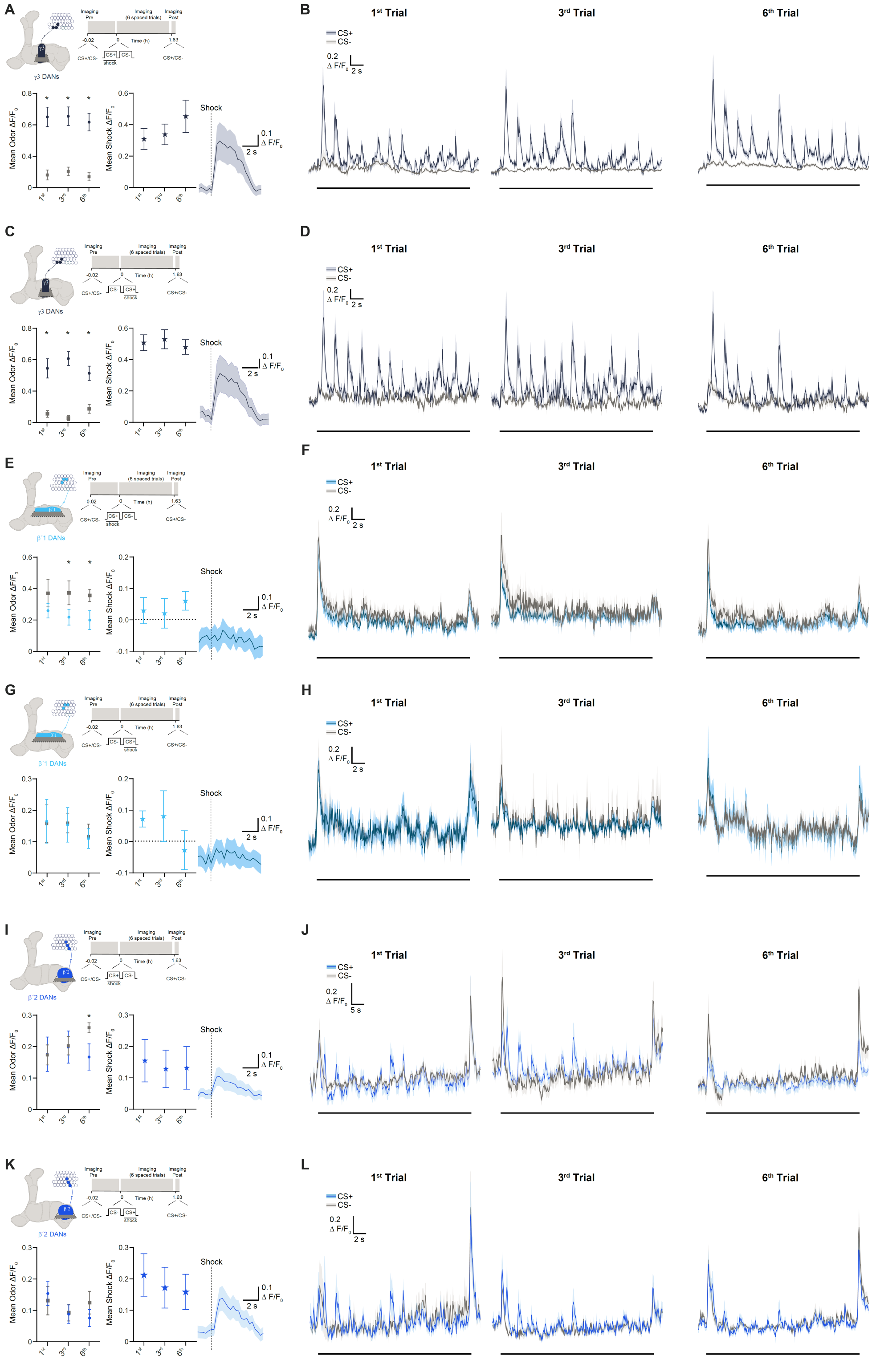

Figure S6. Related to Figure 6.

Spaced training enhanced CS+ responses in aversive γ3 DANs and CS- responses in rewarding β′1 and β′2mp DANs.

(**A and C**) Top: Imaging plane in the presynaptic field of γ3 DANs and training and imaging protocol. (**A**) Bottom left panel: From the 1st training trial the CS+ showed an increased

mean response relative to the CS-. This was also observed during reversed spaced training (**C**). (**A and C**) Bottom right panels: γ3 DANs showed a strong calcium response to electric shock. (**B and D**) Calcium responses during the 1st, 3rd and 6th training trials. Odor-evoked activity traces show mean (solid line) with SEM (shadow). Black line underneath, 60 s odor. (**E**) Top: Imaging plane in the presynaptic field of β′1 DANs and training and imaging protocol. Bottom left: From the 3rd training trial CS- responses were increased relative to CS+ responses. Bottom right: β′1 DANs did not respond to shock with an increase in calcium. (**F**) Calcium responses during the 1st, 3rd and 6th trials of spaced training. Horizontal line denotes odor stimulus. (**G**) Top: Imaging plane in the presynaptic field of β′1 DANs and training and imaging protocol. Bottom left: No differences between the CS+ and CS- responses were observed during reversed spaced training. Bottom right: β′1 DANs did not respond to shock with an increase in calcium. (**H**) Calcium responses during the 1st, 3rd and 6th trials of reversed spaced training. Horizontal line denotes odor stimulus. (**I**) Top: Imaging plane in the presynaptic field of β′2mp DANs and training and imaging protocol. Bottom left: At the 6th training trial, the CS- evoked an increased mean response (over the 60 s of odor presentation) in comparison to the CS+, but not for the other trials analyzed. Bottom right: Aligning the activity of β′2mp DANs to time of each of the 12 shocks (dashed line) reveals a shock-evoked response. Calcium responses were calculated by averaging the fluorescence over the 3 s following each shock. (**J**) β′2mp DAN calcium responses during the 1st, 3rd and 6th trials of spaced training. Horizontal line denotes odor stimulus. (**K**) Top: Imaging plane in presynaptic field of β′2mp DANs and training and imaging protocol. Bottom left: No changes were observed between CS+ and CS- responses during reversed spaced training. Bottom right: β′2mp DANs responded to shock. (**L**) β′2mp DAN calcium responses during the 1st, 3rd and 6th trial of reversed spaced training. Horizontal line denotes odor stimulus. CS+ data corresponds to average data in which 50% of trials used MCH as CS+ and 50% were OCT CS+. Same applies for CS- data. Asterisks denote significant difference between averaged CS+ and CS- responses. See Table S1 for statistics.

Table S1. Statistical details. Related to Figures 1-6 and S1-S6.

Figure	Experiment	n	Normally distributed	Statistical test	p value
Figure 1A	CS+ memory (CS+ vs Novel) vs. LTM (CS+ vs CS-); spaced training	8-13	yes	unpaired t-test t(19)= 2.890	p=0.0094
	CS- vs Novel; spaced training	10	yes	one sample t-test t(9)=3.312	p=0.0091
Figure 1B	CS+ memory (CS+ vs Novel) vs. LTM (CS+ vs CS-); reversed spaced training	8-11	yes	unpaired t-test t(17)=0.6628	p=0.5163
	Performance of CS- vs Novel different from zero; reversed spaced training	8	yes	one sample t-test t(7)=1.546	p=0.1660
Figure 1C	CS+ memory (CS+ vs Novel) vs. LTM (CS+ vs CS-); massed training	10-15	yes	unpaired t-test t(23)=0.7649	p=0.4521
	Performance of CS- vs Novel different from zero; massed training	10	yes	one sample t-test t(9)=1.151	p=0.2792
Figure 1D	CS+ memory (CS+ vs Novel) vs. LTM (CS+ vs CS-); fasting LTM protocol	10-11	yes	unpaired t-test t(19)=1.802	p=0.09
	Performance of CS- vs Novel different from zero; fasting LTM protocol	10	yes	one sample t-test t(9)=0.4190	p=0.6850
Figure 1E	Time-line of CS+ vs. CS- memory (all time points)	8-10	yes	F[5,44]=20.61	p<0.0001
	Immediate vs. 24 h			Dunnett's multiple comparisons test	p=0.0011
	30 min vs. 24 h				p=0.0122
	3 h vs. 24 h				p=0.0043
	14 h vs. 24 h				p=0.9998
	96 h vs. 24 h				p=0.0028
Figure 1F	Time-line of CS- vs. Novel (all time points)	8-10	yes	F[5,44]=4.33	p=0.0028
	Immediate vs. 24h			Dunnett's multiple comparisons test	p=0.0026
	30 min vs. 24 h				p=0.047
	3 h vs. 24 h				p=0.3717
	14 h vs. 24 h				p=0.9997
	96 h vs. 24 h				p=0.8464
	Performance of CS- vs. Novel different from zero @ Immediate time point	8	yes	one sample t-test t(7)=0.6530	p=0.5346
	Performance of CS- vs. Novel different from zero @ 30 min time point	8		one sample t-test(7)=0.9635	p=0.3674
	Performance of CS- vs. Novel different from zero @ 3 h time point	8		one sample t-test(7)=1.931	p=0.0948
	Performance of CS- vs. Novel different from zero @ 14 h time point	8		one sample t-test(7)=5.557	p<0.0001
	Performance of CS- vs. Novel different from zero @ 24 h time point	8		one sample t-test(7)= 9.305	p=0.0009
	Performance of CS- vs. Novel different from zero @ 96 h time point	10		one sample t-test(9)= 2.885	p=0.0180
	Time-line of CS+ vs. Novel (all time points)	8-10	yes	F[5,44]=20.61	p<0.0001
	Immediate vs. 24h			Dunnett's multiple comparisons test	p=0.0011
	30 min vs. 24 h				p=0.0122
	3 h vs. 24 h				p=0.0043
	14 h vs. 24 h				p=0.9921
	96 h vs. 24 h				p=0.0028
	Performance of CS- vs. Novel different from zero @ Immediate timepoint	10	yes	one sample t-test t(9)=0.1127	p=0.9219
Figure 1G	*rad* vs. WT; spaced training LTM (CS+ vs CS-)	7	yes	unpaired t-test t(12)=2.225	p=0.0416
	rad vs. WT; spaced training CS+ memory (CS+ vs Novel)	8	yes	unpaired t-test t(14)=2.969	p=0.0102
	rad vs. WT; spaced training CS- memory (CS- vs Novel)	7	yes	unpaired t-test t(12)=0.2593	p=0.7998
Figure 1G	CXM vs. WT; spaced training LTM (CS+ vs CS-)	7-8	yes	unpaired test t(13)=3.943	p=0.0017
	CXM vs. WT; spaced training CS+ memory (CS+ vs Novel)	9-12	yes	unpaired t-test t(19)=2.703	p=0.0141
	CXM vs. WT; spaced training CS- memory (CS- vs Novel)	11-13	yes	unpaired t-test t(22)=1.720	p=0.0995
Figure 2A	LTM performance (CS+ vs CS-); PAM block	14-15	yes	F[2,41]=24.93	p<0.0001
	R58E02-GAL4; UAS-Shi^{ts1} vs.+; UAS-Shi^{ts1}			Tukey's multiple comparisons test	p<0.0001
	R58E02-GAL4; UAS-Shi^{ts1} vs. R58E02-GAL4; +				p<0.0001
	R58E02-GAL4; + vs.+; UAS-Shi^{ts1}				p=0.4404
Figure 2B	CS+ memory (CS+ vs Novel); PAM block	6	yes	F[2,15]=0.59	p=0.5668
Figure 2C	CS- memory (CS- vs Novel); PAM block	7-8	yes	F[2,19]=26.32	p<0.0001
	R58E02-GAL4; UAS-Shi^{ts1} vs.+; UAS-Shi^{ts1}			Tukey's multiple comparisons test	p<0.0001
	R58E02-GAL4; UAS-Shi^{ts1} vs. R58E02-GAL4; +				p=0.0002
	R58E02-GAL4; + vs.+; UAS-Shi^{ts1}				p=0.3370
Figure 2D	LTM performance (CS+ vs CS-); PAM block during the 1st and 2nd trial	8	yes	F[2,21]=11.40	p=0.0004
	R58E02-GAL4; UAS-Shi^{ts1} vs.+; UAS-Shi^{ts1}			Tukey's multiple comparisons test	p=0.0013
	R58E02-GAL4; UAS-Shi^{ts1} vs. R58E02-GAL4; +				p=0.0014
	R58E02-GAL4; + vs.+; UAS-Shi^{ts1}				p=0.9992
Figure 2E	LTM performance (CS+ vs CS-); PAM block during the 3rd and 4th trial	8	yes	F[2,21]=9.209	p=0.0004
	R58E02-GAL4; UAS-Shi^{ts1} vs.+; UAS-Shi^{ts1}			Tukey's multiple comparisons test	p=0.0272
	R58E02-GAL4; UAS-Shi^{ts1} vs. R58E02-GAL4; +				p=0.0011
	R58E02-GAL4; + vs.+; UAS-Shi^{ts1}				p=0.3565
Figure 2F	LTM performance (CS+ vs CS-); PAM block during the 5th and 6th trial	8	yes	F[2,21]=7.741	p=0.003
	R58E02-GAL4; UAS-Shi^{ts1} vs.+; UAS-Shi^{ts1}			Tukey's multiple comparisons test	p=0.0083
	R58E02-GAL4; UAS-Shi^{ts1} vs. R58E02-GAL4; +				p=0.0062
	R58E02-GAL4; + vs.+; UAS-Shi^{ts1}				p=0.9916

Figure 3A	R71D08-GAL4 after spaced training	28	yes	paired t-test t(27)=4.277	p=0.0002
Figure 3B	G0239-GAL4 after spaced training	20	no	Wilcoxon matched-pairs signed rank W(19)=169	p<0.0001
Figure 3C	R39A05-GAL4 after spaced training, dendrites	28	yes	paired t-test t(27)=3.374	p=0.023
Figure 3D	R39A05-GAL4 after spaced training reverse order, dendrites	24	yes	paired t-test t(23)=0.19	p=0.851
Figure 3E	R39A05-GAL4 after spaced training, presynaptic terminals	34	yes	paired t-test t(33)=2.639	p=0.013
Figure 3F	R39A05-GAL4 after spaced training reverse order, presynaptic terminals	24	yes	paired t-test t(23)=0.1181	P=0.118
Figure 3G	R66C08-GAL4 after spaced training, dendrites	20	yes	paired t-test t(19)=0.1799	p=0.8591
Figure 3H	R66C08-GAL4 after spaced training reverse order, dendrites	20	yes	paired t-test t(19)=0.03199	p=0.9748
Figure 4A	MB110C-GAL4 after spaced training, β′1 dendrites	34	yes	paired t-test t(33)=3.793	p=0.0006
Figure 4B	MB110C-GAL4 after spaced training reverse order, β′1 dendrites	30	no	Wilcoxon matched-pairs signed rank W(29)=31	p=0.749
Figure 4C	MB110C-GAL4 after spaced training, γ3 dendrites	36	no	Wilcoxon matched-pairs signed rank W(35)=308	p=0.011
Figure 4D	MB110C-GAL4 after spaced training reverse order, γ3 dendrites	30	yes	paired t-test t(29)=2.140	p=0.04
Figure 4E	MB110C-GAL4 after spaced training, presynaptic terminals	34	yes	paired t-test t(33)=0.1275	p=0.899
Figure 4F	MB110C-GAL4 after spaced training reverse order, presynaptic terminals	22	yes	paired t-test t(21)=1.535	p=0.14
Figure 5A	LTM performance (CS+ vs CS-); DAN block	11-13	yes	F[4,53]=5.093	p=0.0015
	MB504B-GAL4; UAS-*Shi*^{ts1} vs.+; UAS-*Shi*^{ts1}			Dunnett's multiple comparisons test	p=0.0024
	MB441B-GAL4; UAS-*Shi*^{ts1} vs.+; UAS-*Shi*^{ts1}				p=0.8683
	MB056B-GAL4; UAS-*Shi*^{ts1} vs.+; UAS-*Shi*^{ts1}				p=0.0055
	MB025B-GAL4; UAS-*Shi*^{ts1} vs.+; UAS-*Shi*^{ts1}				p=0.0429
Figure 5B	CS+ memory (CS+ vs Novel); DAN block	8-9	yes	F[4,37]=3.371	p=0.0189
	MB504B-GAL4; UAS-*Shi*^{ts1} vs.+; UAS-*Shi*^{ts1}			Dunnett's multiple comparisons test	p=0.0362
	MB441B-GAL4; UAS-*Shi*^{ts1} vs.+; UAS-*Shi*^{ts1}				p=0.9663
	MB056B-GAL4; UAS-*Shi*^{ts1} vs.+; UAS-*Shi*^{ts1}				p=0.9992
	MB025B-GAL4; UAS-*Shi*^{ts1} vs.+; UAS-*Shi*^{ts1}				p=0.9997
Figure 5C	CS- memory (CS- vs Novel); DAN block	8	yes	F[4,335]=11.76	p<0.0001
	MB504B-GAL4; UAS-*Shi*^{ts1} vs.+; UAS-*Shi*^{ts1}			Dunnett's multiple comparisons test	p=0.8466
	MB441B-GAL4; UAS-*Shi*^{ts1} vs.+; UAS-*Shi*^{ts1}				p=0.0002
	MB056B-GAL4; UAS-*Shi*^{ts1} vs.+; UAS-*Shi*^{ts1}				p=0.0007
	MB025B-GAL4; UAS-*Shi*^{ts1} vs.+; UAS-*Shi*^{ts1}				p=0.0443
Figure 6B	MB441B-GAL4 after spaced training	20	yes	Repeated measures ANOVA, F[19,57]=5.089	p=0.0102
	pretraining CS+ vs. pretraining CS-			Bonferroni's multiple comparisons test	p>0.9999
	pretraining CS+ vs. posttraining CS+				p=0.0388
	pretraining CS+ vs. posttraining CS-				p=0.0001
	pretraining CS- vs. posttraining CS-				p>0.9999
Figure 6D	MB441B-GAL4 after reversed spaced training	20	yes	Repeated measures ANOVA, F[19,57]=6.417	p=0.0066
	pretraining CS+ vs. pretraining CS-			Bonferroni's multiple comparisons test	p>0.9999
	pretraining CS+ vs. posttraining CS+				p<0.0001
	pretraining CS+ vs. posttraining CS-				P=0.0406
	pretraining CS- vs. posttraining CS-				p>0.9999
Figure 6F	MB025B-GAL4 after spaced training	20	yes	Repeated measures ANOVA, F[19,57]= 5.244	p=0.0066
	pretraining CS+ vs. pretraining CS-			Bonferroni's multiple comparisons test	p=0.6615
	pretraining CS+ vs. posttraining CS+				p=0.0254
	pretraining CS+ vs. posttraining CS-				p=0.0019
	pretraining CS- vs. posttraining CS-				p=0.2918
Figure 6H	MB025B-GAL4 after reversed spaced training	20	yes	Repeated measures ANOVA, F[19,57]= 6.063	p=0.0045
	pretraining CS+ vs. pretraining CS-			Bonferroni's multiple comparisons test	p=0.1846
	pretraining CS+ vs. posttraining CS+				p=0.0048
	pretraining CS+ vs. posttraining CS-				p>0.9999
	pretraining CS- vs. posttraining CS-				p=0.045
Figure 6J	MB056B-GAL4 after spaced training	20	yes	Repeated measures ANOVA, F[19,57]=8.042	p=0.0012
	pretraining CS+ vs. pretraining CS-			Bonferroni's multiple comparisons test	p=0.5877
	pretraining CS+ vs. posttraining CS+				p>0.9999
	pretraining CS+ vs. posttraining CS-				p=0.0276
	pretraining CS- vs. posttraining CS-				p=0.0041
Figure 6L	MB056B-GAL4 after reversed spaced training	20	yes	Repeated measures ANOVA, F[19,57]=1.306	p=0.283

Figure S1A	CS+ vs. Air; different training regimens	8	yes	F[2,21]=0.4275	p=0.65
Figure S1B	CS- vs. Air; different training regimens	10	yes	F[2,27]=7.609	p=0.0024
	spaced vs. reversed spaced			Tukey's multiple comparisons test	p=0.0021
	spaced vs. massed				p=0.0313
	reversed spaced vs. massed				p=0.5231
	Performance of CS- vs. Air different from zero; spaced training			one sample t-test t(9)=2.375	p=0.0416
	Performance of CS- vs. Air different from zero; reversed spaced training			one sample t-test t(9)=2.511	p=0.0332
	Performance of CS- vs. Air different from zero; massed training			one sample t-test t(9)=2.595	p=0.0290
Figure S1C	Performance of CS+ vs. CS- (LTM) different from zero; massed training 96 h	7	yes	one sample t-test t(6)=1.434	p=0.2016
	Performance of CS+ vs. Novel different from zero; massed training 96 h			one sample t-test t(6)=0.3854	p=0.7133
	Performance of CS- vs. Novel different from zero; massed training 96 h			one sample t-test t(6)=1.142	p=0.2969
Figure S1D	Performance of spaced relief training 45 s ITI different from zero	8	yes	one-sample t-test t(7)=5.191	p=0.0013
	Performance of spaced relief training 90 s ITI different from zero			one-sample t-test t(7)=2.425	p=0.0411
	Performance of spaced relief training 135 s ITI different from zero			one-sample t-test t(7)=1.597	p=0.1543
Figure S1E	LTM (CS+ vs CS-) performance with varying ITI	12	yes	F[3,44]=1.228	p=0.31
Figure S1F	CS- memory (CS- vs Novel)	11	yes	F[3,40]=1.462	p=0.2393
Figure S2A	LTM performance (CS+ vs CS-); PAM block	14	yes	F[2,39]=7.229	p=0.0021
	R58E02-GAL4; UAS-GtACR1 vs.+; UAS-GtACR1			Tukey's multiple comparisons test	p=0.0370
	R58E02-GAL4; UAS-GtACR1 vs. R58E02-GAL4; +				p=0.0018
	R58E02-GAL4; + vs.+; UAS-GtACR1				p=0.4920
Figure S2B	LTM performance (CS+ vs CS-); PAM block (light controls)	8	yes	F[2,21]=0.2825	p=0.7567
Figure S2C	LTM performance (CS+ vs CS-); PAM block (temperature controls)	6	yes	F[2,15]=1.605	p=0.2335
Figure S2D	CS+ memory (CS+ vs Novel); PAM block (temperature controls)	6	yes	F[2,15]=1.132	p=0.3484
Figure S2E	CS- memory (CS- vs Novel); PAM block (temperature controls)	6	yes	F[2,15]=0.3142	p=0.7351
Figure S2F	LTM performance (CS+ vs CS-); PAM block during the 4th trial	7	yes	F[2,18]=0.9606	p=0.4014
Figure S2G	LTM performance (CS+ vs CS-); PAM block during the 6th trial	8	yes	F[2,21]=0.5720	p=0.5729
Figure S3A	R66C08-GAL4 after spaced training, presynaptic terminals	24	yes	paired t-test t(23)=1.815	p=0.0825
Figure S3B	R66C08-GAL4 after spaced training reverse order, presynaptic terminals	18	yes	paired t-test t(17)=1.464	p=0.1615
Figure S4A	CS+ memory (CS+ vs Novel); γ3, γ3β′1 MBONs block	8	yes	F[2,21]= 0.4088	p=0.6696
Figure S4B	CS- memory (CS- vs Novel); γ3, γ3β′1 MBONs block	7	yes	F[2,18]=7.316	p=0.0047
	+; MB110C-GAL4/UAS-Shi^{ts1} vs.+; UAS-Shi^{ts1}			Tukey's multiple comparisons test	p=0.0042
	+; MB110C-GAL4/UAS-Shi^{ts1} vs. +; MB110C-GAL4				p=0.0431
	+; MB110C-GAL4 vs.+; UAS-Shi^{ts1}				p=0.5292
Figure S5A	LTM performance (CS+ vs CS-); PPL1-DAN block	7	yes	F[2,18]=6.134	p=0.0093
	MB504B-GAL4; UAS-Shi^{ts1} vs.+; UAS-Shi^{ts1}			Tukey's multiple comparisons test	p=0.0290
	MB504B-GAL4; UAS-Shi^{ts1} vs. MB504B-GAL4; +				p=0.0129
	MB504B-GAL4; + vs.+; UAS-Shi^{ts1}				p=0.9216
Figure S5B	CS+ memory (CS+ vs Novel); PPL1-DAN block	7	yes	F[2,18]=10.99	p=0.0008
	MB504B-GAL4; UAS-Shi^{ts1} vs.+; UAS-Shi^{ts1}			Tukey's multiple comparisons test	p=0.0237
	MB504B-GAL4; UAS-Shi^{ts1} vs. MB504B-GAL4; +				p=0.0006
	MB504B-GAL4; + vs.+; UAS-Shi^{ts1}				p=0.2257
Figure S5C	CS- memory (CS- vs Novel); PAM-γ3 DAN block	7	yes	F[2,18]=5.393	p=0.0146
	MB441B-GAL4; UAS-Shi^{ts1} vs.+; UAS-Shi^{ts1}			Tukey's multiple comparisons test	p=0.0233
	MB441B-GAL4; UAS-Shi^{ts1} vs. MB441B-GAL4; +				p=0.0334
	MB441B-GAL4; + vs.+; UAS-Shi^{ts1}				p=0.9833
Figure S5D	LTM performance (CS+ vs CS-); PAM-β′2mp DAN block	7	yes	F[2,18]=5.855	p=0.0110
	MB056B-GAL4; UAS-Shi^{ts1} vs.+; UAS-Shi^{ts1}			Tukey's multiple comparisons test	p=0.0463
	MB056B-GAL4; UAS-Shi^{ts1} vs. MB056B-GAL4; +				p=0.0122
	MB056B-GAL4; + vs.+; UAS-Shi^{ts1}				p=0.8000
Figure S5E	CS- memory (CS- vs Novel); PAM-β′2mp DAN block	7	yes	F[2,18]=5.395	p=0.0146
	MB056B-GAL4; UAS-Shi^{ts1} vs.+; UAS-Shi^{ts1}			Tukey's multiple comparisons test	p=0.0455
	MB056B-GAL4; UAS-Shi^{ts1} vs. MB056B-GAL4; +				p=0.0184
	MB056B-GAL4; + vs.+; UAS-Shi^{ts1}				p=0.9000
Figure S5F	LTM performance (CS+ vs CS-); PAM-β′1 DAN block	7	yes	F[2,18]=8.944	p=0.002
	MB025B-GAL4; UAS-Shi^{ts1} vs.+; UAS-Shi^{ts1}			Tukey's multiple comparisons test	p=0.0239
	MB025B-GAL4; UAS-Shi^{ts1} vs. MB025B-GAL4; +				p=0.0018
	MB025B-GAL4; + vs.+; UAS-Shi^{ts1}				p=0.4695
Figure S5G	CS- memory (CS- vs Novel); PAM-β′1 DAN block	7	yes	F[2,18]=5	p=0.0188
	MB025B-GAL4; UAS-Shi^{ts1} vs.+; UAS-Shi^{ts1}			Tukey's multiple comparisons test	p=0.0261
	MB025B-GAL4; UAS-Shi^{ts1} vs. MB025B-GAL4; +				p=0.0472
	MB025B-GAL4; + vs.+; UAS-Shi^{ts1}				p=0.9546
Figure S5H	LTM performance (CS+ vs CS-); PPL1-DAN block (temperature controls)	7	yes	F[2,18]=1.925	p=0.1747
Figure S5I	CS+ memory (CS+ vs Novel); PPL1-DAN block (temperature controls)	7	yes	F[2,18]=0.8590	p=0.4402
Figure S5J	CS- memory (CS- vs Novel); PAM-γ3 DAN block (temperature controls)	7	yes	F[2,18]=0.02939	p=0.9711

Figure S5K	LTM performance (CS+ vs CS-); PAM-β′2mp DAN block (temperature controls)	7	yes	F[2,18]=0.2763	p=0.7617
Figure S5L	CS- memory (CS- vs Novel); PAM-β′2mp DAN block (temperature controls)	7	yes	F[2,18]=1.053	p=0.3693
Figure S5M	LTM performance (CS+ vs CS-); PAM-β′1 DAN block (temperature controls)	7	Yes	F[2,18]=1.031	p=0.3769
Figure S5N	CS- memory (CS- vs Novel); PAM-β′1 DAN block (temperature controls)	7	yes	F[2,18]=1.064	p=0.3658
Figure S6A	MB441B-GAL4 after spaced training CS+ vs CS- response 1st trial	20	yes	paired t-test(19)=3.219	p=0.0033
	MB441B-GAL4 after spaced training CS+ vs CS- response 3rd trial	20	Yes	paired t-test(19)=3.485	p=0.0017
	MB441B-GAL4 after spaced training CS+ vs CS- response 6th trial	20	yes	paired t-test(19)=3.805	p=0.0007
Figure S6C	MB441B-GAL4 after reversed spaced training CS+ vs CS- response 1st trial	20	yes	paired t-test(19)=4.443	p=0.0004
	MB441B-GAL4 after reversed spaced training CS+ vs CS- response 3rd trial	20	Yes	paired t-test(19)=8.27	p<0.0001
	MB441B-GAL4 after reversed spaced training CS+ vs CS- response 6th trial	20	yes	paired t-test(19)=3.567	p=0.0026
Figure S6E	MB025B-GAL4 after spaced training CS+ vs CS- response 1st trial	20	yes	paired t-test(19)=1.365	p=0.1855
	MB025B-GAL4 after spaced training CS+ vs CS- response 3rd trial	20	Yes	paired t-test(19)=2.161	p=0.0414
	MB025B-GAL4 after spaced training CS+ vs CS- response 6th trial	20	yes	paired t-test(19)=2.172	p=0.0404
Figure S6G	MB025B-GAL4 after reversed spaced training CS+ vs CS- response 1st trial	20	yes	paired t-test(19)=0.0972	p=0.9246
	MB025B-GAL4 after reversed spaced training CS+ vs CS- response 3rd trial	20	Yes	paired t-test(19)=0.0975	p=0.9242
	MB025B-GAL4 after reversed spaced training CS+ vs CS- response 6th trial	20	yes	paired t-test(19)=0.2381	p=0.8166
Figure S6I	MB056B-GAL4 after spaced training CS+ vs CS- response 1st trial	20	no	Wilcoxon matched-pairs signed rank W(19)=24	p=0.8242
	MB056B-GAL4 after spaced training CS+ vs CS- response 3rd trial	20	no	Wilcoxon matched-pairs signed rank W(19)=17	p=0.8968
	MB056B-GAL4 after spaced training CS+ vs CS- response 6th trial	20	no	Wilcoxon matched-pairs signed rank W(19)=202	p=0.0437
Figure S6K	MB056B-GAL4 after reversed spaced training CS+ vs CS- response 1st trial	20	no	Wilcoxon matched-pairs signed rank W(19)=-86	p=0.4319
	MB056B-GAL4 after reversed spaced training CS+ vs CS- response 3rd trial	20	no	Wilcoxon matched-pairs signed rank W(19)=20	p=0.8609
	MB056B-GAL4 after reversed spaced training CS+ vs CS- response 6th trial	20	no	Wilcoxon matched-pairs signed rank W(19)=184	p=0.0872

Table S2. Odor and shock acuity tests. Related to Figures 1, 2, 5 and S1, S2, S5.

Genotype	Odor Acuity						Shock Acuity	
	23°C			32°C			23°C	32°C
	MCH	OCT	IAA	MCH	OCT	IAA		
WT	-0.32±0.06	-0.30±0.06	-0.35±0.05		N.A.		-0.65±0.03	0.60±0.07
rad	-0.34±0.08	-0.31±0.08	-0.37±0.09		N.A.		-0.65±0.04	0.60±0.03
CXM+	-0.33±0.08	-0.30±0.06	-0.30±0.05		N.A.		-0.69±0.03	0.60±0.04
CXM-	-0.36±0.07	-0.37±0.03	-0.28±0.03		N.A.		-0.65±0.03	0.56±0.04
UAS-*Shi*^{ts1}/+	-0.32±0.07	-0.26±0.03	-0.28±0.04	-0.51±0.04	-0.49±0.03	-0.39±0.05	-0.65±0.06	0.61±0.04
UAS-*Shi*$^{ts1;}$ R58E02	-0.28±0.04	-0.27±0.03	-0.31±0.04	-0.40±0.06	-0.49±0.03	-0.45±0.04	-0.65±0.03	-0.62±0.03
R58E02/+	-0.32±0.04	-0.25±0.03	-0.30±0.04	-0.43±0.08	-0.40±0.05	-0.39±0.04	-0.60±0.03	-0.6±0.04
UAS-GtACR1;R58E02	-0.31±0.07	-0.39±0.06	-0.29±0.06		N.A.		-0.68±0.04	N.A.
UAS-GtACR1/+	-0.32±0.05	-0.34±0.04	-0.35±0.08		N.A.		-0.69±0.05	N.A.
UAS-*Shi*^{ts1};MB504B	-0.36±0.03	-0.29±0.04	-0.30±0.04	-0.51±0.03	-0.41±0.07	-0.34±0.04	-0.77±0.05	0.63±0.04
UAS-*Shi*^{ts1};MB056B	-0.33±0.07	-0.24±0.06	-0.36±0.06	-0.44±0.04	-0.45±0.04	-0.40±0.04	-0.78±0.03	0.66±0.02
UAS-*Shi*^{ts1};MB441B	-0.29±0.05	-0.33±0.03	-0.33±0.03	-0.49±0.04	-0.47±0.05	-0.34±0.03	-0.64±0.07	0.55±0.02
UAS-*Shi*^{ts1};MB025B	-0.36±0.03	-0.37±0.06	-0.29±0.04	-0.41±0.05	-0.51±0.04	-0.39±0.06	-0.72±0.05	0.7±0.02
MB504B/+	-0.30±0.05	-0.29±0.02	-0.34±0.06	-0.46±0.09	-0.54±0.05	-0.31±0.07	-0.68±0.03	0.7±0.04
MB056B/+	-0.32±0.04	-0.27±0.05	-0.31±0.06	-0.40±0.04	-0.54±0.05	-0.37±0.04	-0.68±0.04	0.59±0.04
MB441B/+	-0.30±0.02	-0.29±0.02	-0.37±0.05	-0.47±0.04	-0.47±0.05	-0.35±0.06	-0.63±0.05	0.56±0.04
MB025B/+	-0.31±0.03	-0.34±0.05	-0.30±0.03	-0.44±0.06	-0.49±0.03	-0.36±0.04	-0.67±0.05	0.61±0.05

There are no statistical differences between the relevant groups. Data is displayed Mean ± SEM and n=7 for all groups.

N.A. not applicable
