# Supplementary material for: Spaced Training Forms Complementary Long-Term Memories of Opposite Valence in Drosophila
Source: Neuron. 2020 Jun 17;106(6):977–991.e4. doi: 10.1016/j.neuron.2020.03.013 (PMC7302427; doi:10.1016/j.neuron.2020.03.013)
Supplement: Document S2. Article plus Supplemental Information [file mmc2.pdf]

Neuron

Article

Spaced Training Forms Complementary Long-Term Memories of Opposite Valence in *Drosophila*

Highlights

- Spaced training forms complementary long-term aversive and safety memories
- Safety memory is protein-synthesis-dependent long-term memory
- Safety memory acquisition requires repetition, order, and spacing of trials
- Specific dopaminergic neurons reinforce the delayed recognition of safety

Authors

Pedro F. Jacob, Scott Waddell

Correspondence

scott.waddell@cncb.ox.ac.uk

In Brief

Multiple trials of aversive olfactory conditioning in *Drosophila* can form an aversive memory for one odor and an unexpected safety memory for the other. Although safety memory appears slowly after training, it lasts longer than aversive memory.

Jacob & Waddell, 2020, Neuron *106*, 977–991
June 17, 2020 © 2020 The Author(s). Published by Elsevier Inc.
https://doi.org/10.1016/j.neuron.2020.03.013

CellPress

Neuron

CellPress
OPEN ACCESS

Article

Spaced Training Forms Complementary Long-Term Memories of Opposite Valence in *Drosophila*

Pedro F. Jacob[1] and Scott Waddell[1,2,3,*]
[1]Centre for Neural Circuits and Behaviour, University of Oxford, Oxford OX1 3TA, UK
[2]Twitter: @scottishwaddell
[3]Lead Contact
*Correspondence: scott.waddell@cncb.ox.ac.uk
https://doi.org/10.1016/j.neuron.2020.03.013

SUMMARY

Forming long-term memory (LTM) often requires repetitive experience spread over time. Studies in *Drosophila* suggest aversive olfactory LTM is optimal after spaced training, multiple trials of differential odor conditioning with rest intervals. Memory after spaced training is frequently compared to that after the same number of trials without intervals. Here we show that, after spaced training, flies acquire additional information and form an aversive memory for the shock-paired odor and a slowly emerging and more persistent "safety-memory" for the explicitly unpaired odor. Safety-memory acquisition requires repetition, order, and spacing of the training trials and relies on triggering specific rewarding dopaminergic neurons. Co-existence of aversive and safety memories is evident as depression of odor-specific responses at different combinations of junctions in the mushroom body output network; combining two outputs appears to signal relative safety. Having complementary aversive and safety memories augments LTM performance after spaced training by making the odor preference more certain.

INTRODUCTION

Memory allows animals to anticipate forthcoming meaningful events and use learned predictive sensory cues to guide preemptive behavior. Across the animal kingdom, forming long-term memory (LTM) often requires multiple training trials with intervening rest periods, or intertrial intervals (ITIs) (Ebbinghaus, 1913; Carew et al., 1972; Tully et al., 1994; Kogan et al., 1997; Hermitte et al., 1999; Menzel et al., 2001).

Acquisition of aversive LTM in *Drosophila* is considered to require five to ten spaced training trials with a 15 min ITI, where an individual trial pairs one of two odors with an electric-shock reinforcement. In contrast, the same number of trials without an ITI, referred to as massed training, only forms a distinct consolidated type of memory referred to as anesthesia-resistant memory (ARM) (Tully et al., 1994). Many studies have reported molecular mechanisms that differentiate between ARM and LTM. For example, flies mutant for the *radish* (*rad*) gene, which encodes a putative Rap GTPase activating protein (Folkers et al., 2006), specifically lack aversive ARM, whereas pharmacological and genetic blockers of new transcription and protein synthesis only disrupt LTM (Tully et al., 1994; Yin et al., 1994; Dubnau et al., 2003; Chen et al., 2012; Miyashita et al., 2012).

Prevailing models suggest that optimal interval timing coincides with the dynamics of cellular signaling processes that are essential for LTM (Zhang et al., 2012; Liu et al., 2013; Smolen et al., 2016). The 15 min ITI in *Drosophila* spaced training coincides with the peak of the training-induced activity of the extracellular signal-regulated kinase (ERK, aka MAPK) (Pagani et al., 2009; Miyashita et al., 2018). Similar to mechanisms of plasticity in other species, activated ERK phosphorylates and activates gene expression driven by the cAMP-response element binding (CREB) transcription factor (Bartsch et al., 1998; Impey et al., 1998; Thomas and Huganir, 2004; Miyashita et al., 2018). After spaced training in *Drosophila,* CREB activation induces expression of the c-Fos transcription factor, encoded by the *kayak* gene. In turn, c-Fos is required to activate CREB, and a CREB-cFos positive feedback loop prolongs the increased CREB activity that is essential to sustain LTM (Miyashita et al., 2018). In the mouse elevated CREB activity appears to provide an eligibility trace—it increases the likelihood that neurons become part of a memory engram (Han et al., 2007; Zhou et al., 2009; Park et al., 2016). Consistent with this model, spaced training produces more c-Fos-positive Kenyon cells (KCs) in the fly mushroom body (MB) and blocking output from all c-Fos-labeled neurons impairs expression of LTM (Miyashita et al., 2018).

Research in *Drosophila* has also provided a neural-circuit context for memory formation and retrieval. Subsets of anatomically restricted dopaminergic neurons (DANs) provide reinforcement signals that modulate connections between MB KCs and MB output neurons (MBONs), whose dendrites occupy the same MB compartment as the DANs (Claridge-Chang et al., 2009; Aso et al., 2010; Burke et al., 2012; Liu et al., 2012; Lin et al., 2014). DAN activity coincident with odor exposure depresses synaptic connections between sparse populations of odor-activated KCs and MBONs (Séjourné et al., 2011; Hige et al., 2015; Owald et al., 2015; Cohn et al., 2015; Perisse et al., 2016; Handler et al., 2019) via a dopamine-receptor-directed cAMP-dependent plasticity (Yu et al., 2006; Kim et al., 2007; Tomchik and Davis, 2009; Qin et al., 2012; Zhang and Roman, 2013;

 © 2020 The Author(s). Published by Elsevier Inc.
This is an open access article under the CC BY license (http://creativecommons.org/licenses/by/4.0/).

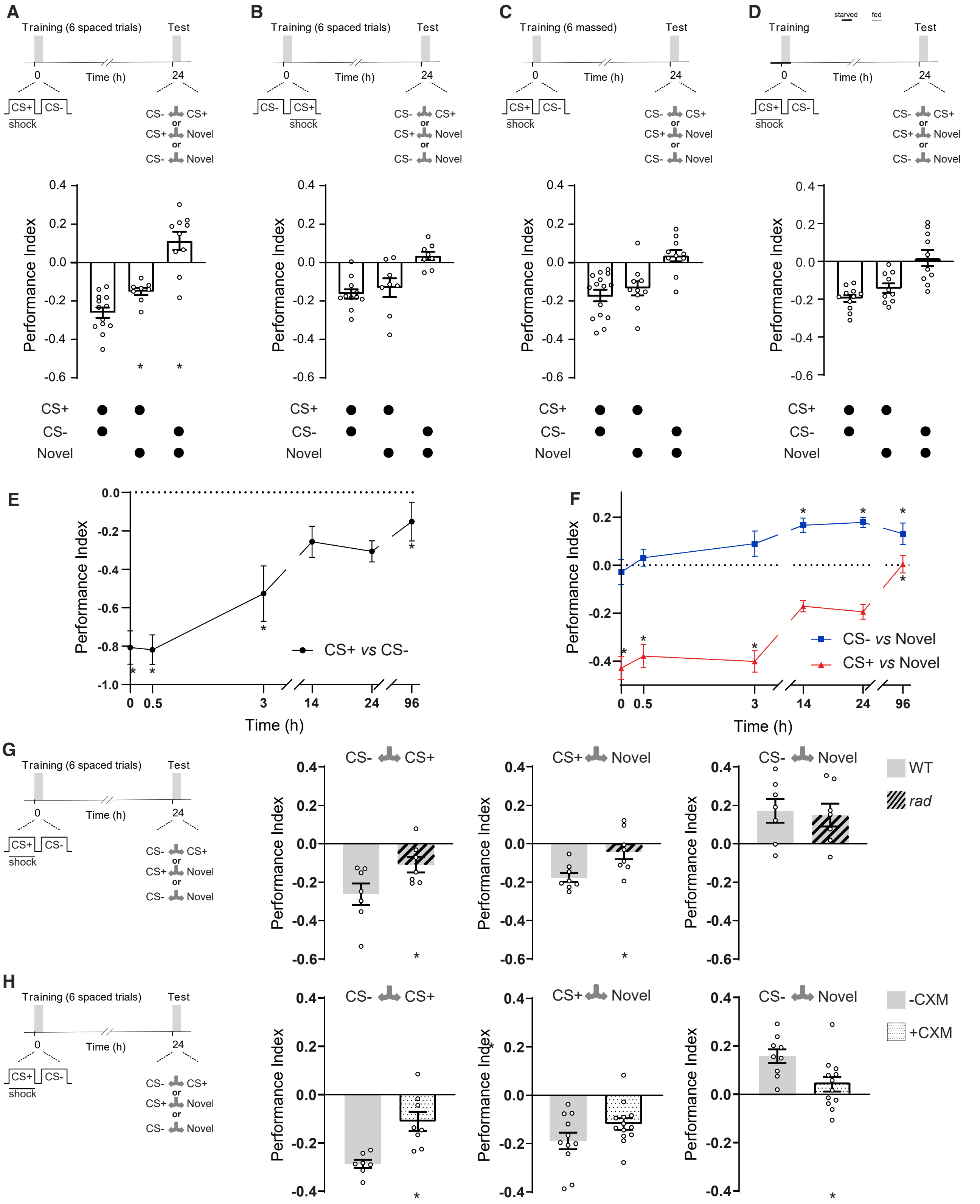

(legend on next page)

Boto et al., 2014; Hige et al., 2015; Handler et al., 2019). Aversive learning reduces the odor drive to approach directing MBONs, which primarily occupy the vertical MB lobe. In contrast, appetitive learning reduces the responses of avoidance directing MBONs, mainly on the tips of the horizontal MB lobes. Memory formation therefore establishes different configurations of the MBON network, and the trained odors subsequently drive the skewed output (Séjourné et al., 2011; Hige et al., 2015; Owald et al., 2015; Perisse et al., 2016). Aversive LTM expression after spaced training strongly relies on αβ KCs and downstream vertical lobe MBONs (MB-V2 aka MBON-α2sc, MBON-α′3m and MBON-α′3p, and MB-V3 aka MBON-α3) (Tanaka et al., 2008; Aso et al., 2014a) that pool outputs from the vertical α collaterals of αβ KCs (Pascual and Préat, 2001; Isabel et al., 2004; Yu et al., 2006; Séjourné et al., 2011; Huang et al., 2012; Bouzaiane et al., 2015). However, γ and α′β′ KCs have also been implicated in LTM, either directly or by virtue of a requirement for downstream MBONs, such as MBON-γ3, MBON-γ3β′1, and MBON-M4β′2mp (Akalal et al., 2010; Wu et al., 2017). The network requirements for aversive LTM expression are also evidently different from those for expression of 24 h ARM (Bouzaiane et al., 2015; Wu et al., 2017). However, although ARM and LTM differ at the molecular and circuit levels, it is not clear whether flies acquire comparable information, or memory content, after spaced and massed training.

Flies can simultaneously, or sequentially, form parallel avoidance and approach memories that compete to guide memory-directed behavior (Das et al., 2014; Aso and Rubin, 2016; Felsenberg et al., 2017, 2018). Initial memory performance is additive if one odor is paired with shock and the other odor with sugar during conditioning (Tempel et al., 1983). Here we show that spaced, but not massed, training gives flies the opportunity to learn that the shock-paired odor (conditioned stimulus +, CS+) is to be avoided and the non-reinforced odor (CS−) is safe. Learning that an odor is safe requires the odor to be presented after a shock-paired odor in each of at least five spaced training trials. The formation of safety memory needs the activity of two classes of rewarding DANs, whose safe-odor-driven activity increases as training progresses. Parallel aversive and safety memories can be recorded as depression of odor-specific responses in a distributed collection of unique MBONs. In addition, plasticity of MBON-γ3,γ3β′1 connections is required for flies to learn relative safety. LTM performance after spaced training therefore arises from the addition of complementary odor-specific avoidance and approach memories.

RESULTS

Spaced Training Forms Two Memories of Opposite Valence

Learning in different ways produces memories of distinct duration. In *Drosophila*, studies frequently compare differences in memory formed after a number of training trials with or without ITIs. Although five to ten differential odor-shock training trials form a memory that can be measured 24 h later, the underlying molecular and network processes are clearly different if training is spaced or massed (Tully et al., 1994). We therefore first investigated whether distinguishable neural correlates of spaced training might arise from the flies' learning different information than when taught with massed trials.

We conditioned flies using a spaced training protocol of six differential training trials separated by 15 min intervals (Figure 1A). In agreement with prior studies, this regimen induced a persistent 24 h LTM. Flies selectively avoided the previously shock-paired odor (CS+) when given the choice between that odor and the previously non-reinforced odor (CS−). Interestingly, if flies were instead tested for preference between CS+ and novel odor, they exhibited significantly reduced memory performance in comparison with the CS+ versus CS− condition. Flies also avoided CS+ if given the choice between CS+ and a clean air stream (Air) (Figure S1A). More surprisingly, if spaced-trained flies were tested for preference between the CS− and novel odor, or CS− versus Air, they showed significant approach to the CS− (Figures 1A and S1B). These data are consistent with the idea that spaced training forms a CS+ avoidance memory and a CS− approach memory and that both contribute to 24 h performance.

In each trial of standard spaced training, the CS+ precedes the CS−, which in principle could allow flies to recognize that the CS− is not reinforced and is perhaps 'safe'. To challenge this mechanism, we reversed CS+ and CS− order so that in each trial CS− instead came before CS+ (Figure 1B). After reversed spaced training, flies displayed CS+ versus novel memory that was indistinguishable from CS+ versus CS− performance.

Figure 1. Spaced Training Induces LTM Comprised of Complementary CS+ and CS– Components

(A) Spaced training (six trials of CS+/CS− training with 15 min ITIs) generates 24 h LTM measurable when testing CS+ versus CS− odors. An aversive 24 h memory was measured when testing CS+ versus a novel odor, and this memory was reduced in comparison to the CS+ versus CS− 24 h performance. An appetitive 24 h memory was measured when CS− was tested against novel odor.

(B and C) If CS+ and CS− order was reversed during training (B) or intervals were omitted between training trials, massed training (C), the LTM (CS+ versus CS−) was not different from the aversive CS+ memory, and no approach was observed to CS−.

(D) A fasting LTM protocol that lacks repetition did not generate CS− approach memory, and CS+ memory was not different from that seen after training on CS+ versus CS−.

(E) Timeline of CS+ versus CS− memory performance after spaced training. Performance decays quickly for the first 3 h and stabilizes from 14 to 24 h. 96 h performance was reduced in comparison with 24 h performance.

(F) Timelines of CS+ versus novel odor (red) and CS− versus novel odor (blue) memory after spaced training. Significant CS− memory was only observed from 14 h and persisted for at least 96 h. The CS− memory was not significantly different from zero before 14 h. CS+ memory decayed between 3 and 14 h and remained constant between 14 and 24 h, and no 96 h performance was observed.

(G) *rad* mutant flies (hashed bars), in comparison to wild-type (WT) (Canton-S) flies (gray bars), had impaired LTM performance and lacked CS+ avoidance memory, but displayed normal CS− approach memory.

(H) CXM feeding impaired LTM performance after spaced training, causing a specific defect of CS− but not CS+ memory. WT flies fed 5% glucose (gray bars) or glucose laced with 35 mM CXM (stippled bars) for 12–16 h overnight before spaced training. Asterisks denote significant differences. Data are represented as means ± standard error of the mean (SEM). Individual data points are displayed as dots. See also Figure S1 and Table S1 for statistics.

Testing CS+ versus Air also revealed avoidance of CS+ (Figure S1A). However, no approach to CS− was evident when flies were tested between CS− and a novel odor, and flies avoided CS− when tested with CS− versus Air (Figure S1B). Therefore, the order of CS+ before CS− is needed to form CS− approach memory after spaced training.

Given the reported difference in cellular requirements between spaced and massed training, we tested whether six trials of massed training involving CS+ followed by CS− (CS+/CS−) formed CS+ avoidance and CS− approach memories (Figure 1C). Mass trained flies did not show evidence of CS− approach memory. Their CS+ versus novel performance was indistinguishable from their CS+ versus CS− performance. Testing CS+ versus Air also showed avoidance of CS+ (Figure S1A). However, when tested with CS− versus a novel odor, flies showed no preference and significantly avoided the CS− when tested with CS− versus Air (Figure S1B). Intervals between training trials are therefore critical to the formation of CS− approach memory.

Hunger apparently changes the rules for the formation of aversive LTM so that a single training trial is more effective (Hirano et al., 2013). We therefore tested the nature of fasting LTM (Figure 1D). Flies were starved for 12–16 h and then subjected to one round of CS+/CS− aversive training. The flies were fed after training and tested for 24 h memory. Although flies displayed significant 24 h memory, they did not exhibit CS− approach memory when tested with CS− versus a novel odor. Moreover, their performance on CS+ versus a novel odor was indistinguishable to when tested CS+ versus CS−. Therefore, multiple trials are essential to form complementary CS+ avoidance and CS− approach memories.

Next, we generated a timeline of formation and duration of CS+ avoidance and CS− approach memories. Flies were spaced trained and tested for CS+ versus CS−, CS+ versus novel, and CS− versus novel preference immediately, 30 min, 3 h, 14 h, 24 h, and 96 h after training (Figures 1E and 1F). As previously reported, CS+ versus CS− memory decayed rapidly within 14 h, and this level of performance remained at 24 h. Preference for CS− over CS+ was still apparent at 96 h (Figure 1E). The CS+ versus novel performance followed a similar initial decay to CS+ versus CS−. However, no CS+ versus novel performance remained at 96 h (Figure 1F). In contrast, testing CS− versus novel revealed that CS− approach memory emerged slowly, reaching significance at 14 h and remaining stable until at least 96 h (Figure 1F). These data demonstrate that 24 h performance after spaced training is comprised of CS+ avoidance and CS− approach memories and that the CS− approach memory persists for 96 h.

CS+ but Not CS– Memory Is Impaired in *rad* Mutant Flies

Seminal work concluded that spaced training forms protein-synthesis-dependent LTM, whereas massed training forms protein-synthesis-independent but *rad*-dependent consolidated ARM (Tully et al., 1994; Isabel et al., 2004). Since *rad* mutation (Folkers et al., 1993, 2006) is reported to specifically impair aversive ARM, we tested *rad* mutant flies for CS+ and CS− memory (Figure 1G). After spaced training, *rad* mutants showed impaired 24 h performance. Surprisingly, *rad* mutants displayed normal CS− approach memory but lacked CS+ memory. In addition, no 96 h memory was measurable after massed training (Figure S1C), which should only form ARM (Tully et al., 1994). We also tested the memory of flies fed with cycloheximide (CXM) before training (Figure 1H). As previously reported, feeding CXM 12–16 h before spaced training (Tully et al., 1994; Yin et al., 1994) impaired 24 h performance when flies were tested with CS+ versus CS−. Whereas CXM-fed flies lacked CS− memory, aversive CS+ memory performance was not significantly altered. These experiments indicate that CS− approach memory is the CXM-sensitive LTM component of 24 h performance and CS+ avoidance memory is ARM, which also accounts for why only CS− memory persists for 96 h (Figure 1F).

Reinforcement of CS– Approach Memory Is Not Triggered by Relief

Flies can form odor approach memories if electric shock precedes odor presentation by up to 45 s. In this paradigm, the odor is assumed to gain positive value by association with relief from punishment (Tanimoto et al., 2004; König et al., 2018). Since shocks precede CS− by 45 s in each trial of spaced training, we tested whether CS− approach memory could be persistent relief memory or conditioned inhibition (Pavlov, 1927).

We trained flies by using a spaced relief paradigm that resembled regular spaced training except that the shock was presented alone rather than paired with the first odor. Flies were given 1 min of shock (12 shocks at 5 s intervals) followed 45 s later by 1 min of odor, and this procedure was repeated another five times at 15 min intervals. Spaced-relief training induced significant odor approach if flies were tested immediately (Figure S1D), but performance did not persist for 24 h (performance index = −0.06; one sample t test, $t(6) = 2$, $p = 0.09$). Moreover, if the inter-stimulus interval (ISI) between shock and odor was increased to 90 or 135 s, approach memory was significantly diminished (Figure S1D). These characteristics of relief memory differ from those of the CS− approach memory generated by spaced training; the latter is not observed immediately but emerges between 3 and 14 h after training. In addition, forming CS− approach memory with spaced training is less sensitive to ISI extension between CS+ and CS− (Figures S1E and S1F). We therefore conclude that CS− approach memory is not a relief memory.

Formation of CS– Approach Memory Requires Rewarding Dopaminergic Neurons

Several studies have established that some DANs in the protocerebral anterior medial (PAM) cluster can provide reward-specific teaching signals during learning (Burke et al., 2012; Liu et al., 2012; Lin et al., 2014) (Figure 2A). We therefore tested whether their output was required to form CS− approach memory after aversive spaced training (Figure 2). We expressed the dominant-negative temperature-sensitive UAS-*Shi*^{ts1}-encoded dynamin (Kitamoto, 2001) in PAM DANs by using R58E02-GAL4. We specifically blocked output from R58E02 DANs during spaced training by raising the temperature of flies from 23°C to 32°C. Flies were then returned to 23°C and later tested for 24 h

CellPress
OPEN ACCESS

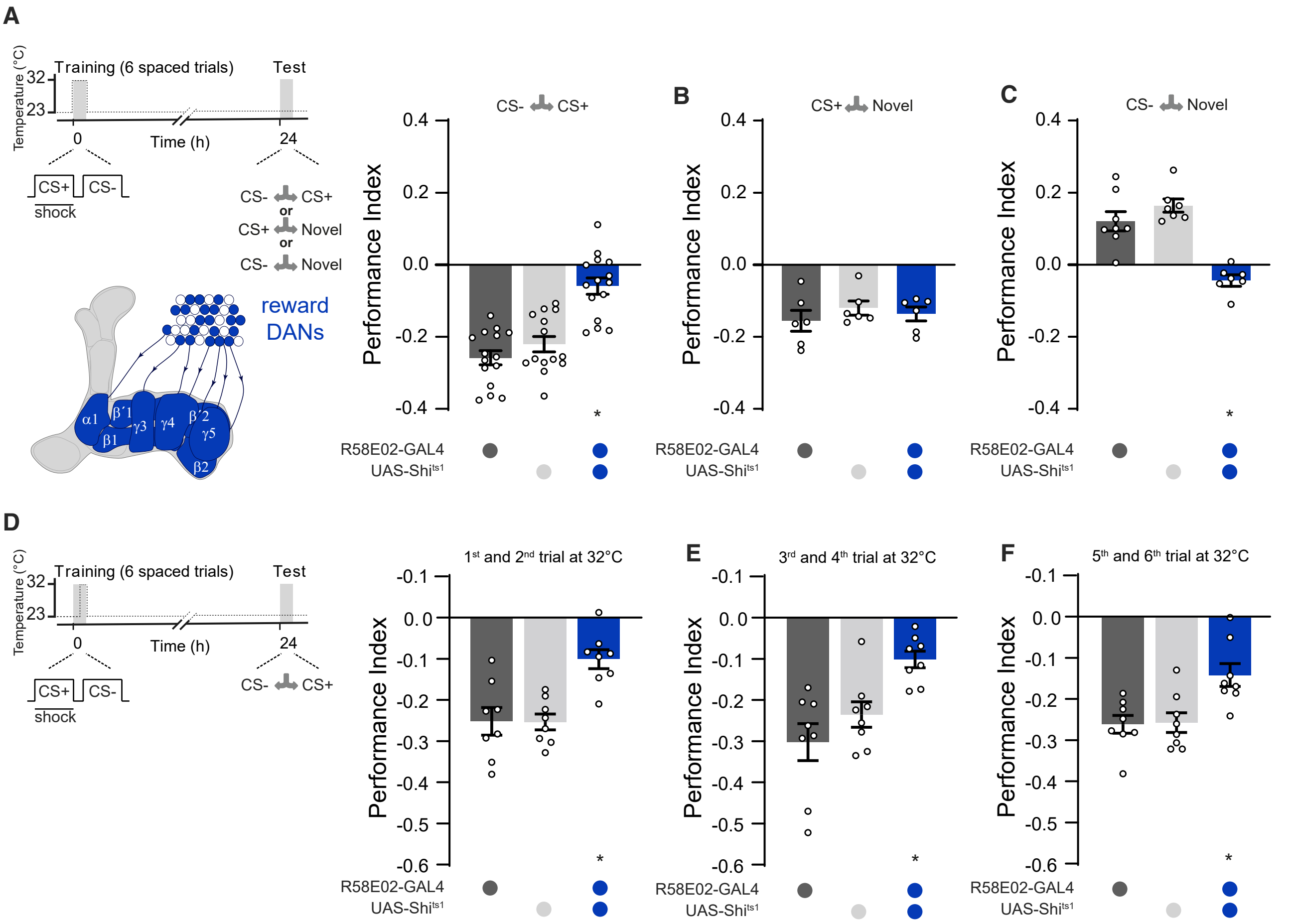

Figure 2. CS– Approach Memory Requires PAM DANs during CS– Presentation

(A) Left: protocol with temperature shifting (dashed line) and schematic depiction of PAM DANs. Right: blocking PAM DANs with R58E02-GAL4/UAS-*Shi*^{ts1} during spaced training impaired 24 h memory performance.

(B) Aversive memory to CS+ was not affected.

(C) Blocking PAM DANs specifically impaired CS− approach memory.

(D) Left: protocol with temperature shifting (dashed line). Right: blocking PAM DANs with R58E02-GAL4/UAS-*Shi*^{ts1} during the first two spaced training trials reduced 24 h memory performance.

(E and F) 24 h memory performance was also reduced when the DAN block was restricted to (E) the third and fourth training trials or (F) the fifth and sixth trials. Asterisks denote a significant difference. Data are represented as means ± SEM. Individual data points are displayed as dots. See also Figure S2 and Table S1 for statistics.

memory (Figure 2A). This manipulation impaired performance when flies were tested for CS+ versus CS− preference. Flies tested CS+ versus novel odor revealed that aversive CS+ memory was relatively unaffected (Figure 2B). However, no performance was evident when flies were tested with CS− versus a novel odor (Figure 2C). Blocking rewarding DANs therefore specifically impaired formation of CS− approach memory.

We next used the light-gated GtACR1 anion channel (Mohammad et al., 2017) to restrict DAN inactivation to the time of CS− presentation during each training trial. This manipulation caused a similar impairment to 24 h CS+ versus CS− performance, as did blocking DANs throughout all of spaced training (Figure S2A). These data are consistent with the idea that some PAM DANs are required for reinforcement of CS− approach memory during aversive spaced training.

We also tested the importance of trial repetition by using UAS-*Shi*^{ts1} to block R58E02 neurons during select training trials (Figures 2D–2F). We imposed the block by raising temperature to 32°C immediately after the last trial performed at permissive 23°C. Training then resumed at 32°C, and at the end of the last trial at restrictive temperature, flies were returned to 23°C for more training or for testing of 24 h CS+ versus CS− performance. Blocking R58E02 neurons for the first, middle, or last two of the six spaced training trials significantly impaired 24 h memory (Figures 2D–2F). However, blocking only the fourth or sixth trial did not impair 24 h CS+ versus CS− performance (Figures S2F and S2G). These results suggest that formation of CS− approach memory requires PAM DAN output during at least five spaced but not necessarily consecutive training trials.

CS+ Avoidance and CS– Approach Memories Co-exist in the MBON Network

Prior studies have reported plasticity of odor-specific responses in MB-V2 (MBON-α2sc) and MB-V3 (MBON-α3) as a correlate of aversive LTM after spaced training. α2sc-MBONs exhibit a reduced response to CS+ after aversive training, and their output is required for the expression of aversive LTM (Séjourné et al., 2011). Plasticity and the role of α3-MBONs is more contentious. Pai et al. (2013) reported that α3-MBONs are required to express aversive LTM; they also reported an increased response to CS+ after spaced but not massed aversive training. However, Plaçais et al. (2013) reported an increased response to CS+ only after appetitive training and that α3-MBON output was dispensable for retrieval of aversive LTM. In contrast, appetitive memories, such as those reinforced by sugar, induce relative depression of responses to CS+ in processes of horizontal lobe M4β′ (MBON-β′2mp) and M6 (MBON-γ5β′2a) (Owald et al., 2015). Lastly, aversive memory can be extinguished by the formation of a parallel appetitive memory that manifests as a reduced response to CS+ in MBON-γ5β′2a (Felsenberg et al., 2018). We therefore used *in vivo* calcium imaging to test for odor-evoked physiological correlates of CS+ aversive and CS− approach memories in these MBONs after spaced training. Ca^{2+} imaging was performed 24 h after training, so flies were trained in the T-maze and captured and mounted briefly before imaging.

We first attempted to reproduce previously reported physiological correlates of aversive LTM in vertical lobe α2sc- and α3-MBONs. Consistent with prior work (Séjourné et al., 2011), we observed a significantly reduced response to CS+ in the α2sc-MBON dendrites after spaced training (Figure 3A). However, contrary to both prior reports (Plaçais et al., 2013; Pai et al., 2013), we also observed strong depression of CS+-evoked responses in the α3-MBON dendrite (Figure 3B). Decreased responses to CS+ in both types of vertical-lobe MBONs after aversive spaced training is consistent with the idea that memory-directed CS+ odor avoidance arises from reduced α2sc- and α3-MBON-mediated odor approach (Aso et al., 2014a). Next, we tested for evidence of CS− approach memory by recording odor-evoked responses in MBON-β′2mp and MBON-γ5β′2a dendrites. Because our experiments in Figure 1 indicated that the order of CS+ then CS− presentation was important for the generation of CS− approach memory, we compared odor-evoked MBON responses from flies spaced trained with CS+/CS− ordered trials (spaced training) to those from flies spaced trained with CS−/CS+ trials (reversed spaced training). Reversed spaced training provides a better control for imaging than massed training does because flies are exposed to the same number of differential trials and intervals as in spaced training. In addition, like massed training, reversed spaced training only forms aversive CS+ memory (ARM). A reduced CS− response was measured in the dendritic and axonal fields of the β′2mp MBON (Figures 3C and 3E) in CS+/CS− spaced trained flies, but not when trials were reversed to CS−/CS+ (Figures 3D and 3F). The statistical significance of these results remained when apparent outliers were removed or when data were randomly subsampled so that sample numbers were equalized (data not shown). No significant change in odor-evoked responses was measured in MBON-γ5β′2a dendrites 24 h after spaced training with either CS+/CS− (Figures 3G and 3H) or CS−/CS+ trials (Figures S3A and S3B). A reduced CS− response in MBON-β′2mp after spaced training could, at least partially, contribute to the conditioned approach to the CS− odor.

Aversive Spaced Training Induces Region-Specific Plasticity in the Dendrites of γ3 and γ3β′1 MBONs

The γ3 and γ3β′1 MBONs have also been implicated in aversive LTM (Wu et al., 2017). Because their dendrites occupy MB compartments that are innervated by PAM DANs, we used Ca^{2+} imaging to test for odor-evoked physiological correlates of CS− approach memory in these MBONs. The MB110C split-GAL4 driver expresses GCaMP in both γ3 and γ3β′1 MBONs. After spaced training, we observed strikingly different responses in the γ3 and β′1 dendritic fields, which are innervated by distinct PAM DANs. Recordings in the β′1 region revealed a reduced CS− response after spaced training (Figure 4A), but not when CS+ and CS− order was reversed (Figure 4B). In contrast, a reduced response to CS+ was evident in the γ3 dendrites after spaced training, but irrespective of CS+/CS− order (Figures 4C and 4D). Interestingly, recording in the presynaptic terminals of γ3,γ3β′1-MBONs after training (Figures 4E and 4F) suggested that these neurons integrate plasticity formed in the γ3 and β′1 dendritic arbors. The relatively decreased CS− response in β′1 appeared nullified when integrated with the relatively decreased response to CS+ in γ3. However, no significant difference was observed when flies were trained with reversed CS−/CS+ trials (Figure 4F). Interestingly, blocking γ3,γ3β′1-MBON output with UAS-Shi^{ts1} during testing selectively impaired expression of CS− approach but not of CS+ avoidance memory (Figures S4A and S4B).

Distributed Plasticity Is Required for Memory after Spaced Training

Finding depression of CS− responses in MBON-β′2mp (Figure 3D) and the β′1 tuft of the MBON-γ3β′1 dendrite (Figures 4A and 4C), and decreased responses to CS+ in α2sc- and α3-MBON dendrites (Figures 3A and 3B), suggests roles for the related DANs in LTM. We therefore used DAN-specific control of UAS-Shi^{ts1} to test the importance of each site of plasticity for CS+ and CS− memories after spaced training.

The α2sc- and α3-MBON compartments on the MB vertical lobe are innervated by a subset of aversively reinforcing DANs from the paired posterior lateral 1 (PPL1) cluster (Figure 5A). As expected, using UAS-MB504B-driven UAS-Shi^{ts1} to block PPL1-DAN output during training severely impaired performance when flies were tested CS+ versus CS− (Figure 5A). PPL1-DAN block specifically impaired CS+ aversive memory. No performance was observed when flies were tested with CS+ versus a novel odor (Figure 5B), whereas significant approach remained when flies were tested with CS− versus a novel odor (Figure 5C).

We next tested the importance of DAN-directed plasticity in MBON-β′2mp and the two regions of the MBON-γ3,γ3β′1 dendritic fields for LTM formation. Blocking PAM-β′2mp or PAM-β′1 DANs during training significantly impaired LTM performance when flies were tested with CS+ versus CS− (Figure 5A).

CellPress
OPEN ACCESS

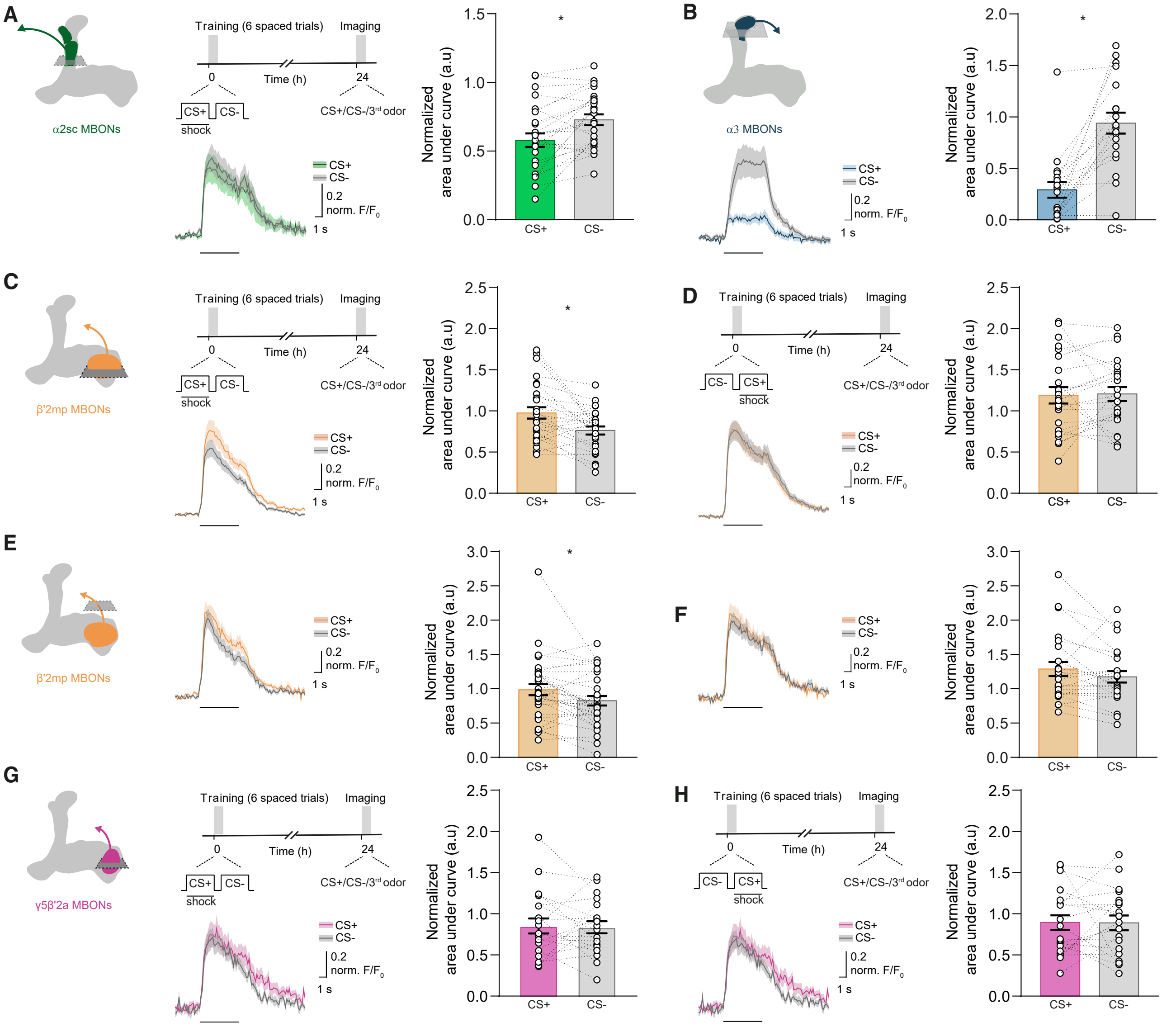

Figure 3. Parallel Aversive and Safety Memories Can Be Recorded as Depression of Odor-Specific Responses in Corresponding MBONs

(A and B) Imaging planes in MBON-α2sc (A) and MBON-α3 (B) dendritic fields and training and imaging protocol. A reduced CS+ odor-evoked response was observed in both MBONs.

(C–F) Imaging plane in the MBON-β′2mp dendritic field (C and D) or presynaptic terminals (E and F) and training and imaging protocol. Spaced training significantly reduced responses to CS− in MBON-β′2mp dendritic (C) and axonal fields (E), but not when CS+ followed CS− in reversed spaced training (D and F).

(G and H) Imaging plane in MBON-γ5β′2a dendritic field and training and imaging protocol. Neither spaced training (G) nor reversed spaced training (H) changed the odor-evoked responses of MBON-γ5β′2a. CS+ data correspond to average of experiments in which 50% of the trials used 4-methylcyclohexanol (MCH) as CS+ and 50% used 3-octanol (OCT) as CS+. The same applies for CS− data. Odor-evoked activity traces show means (solid line) with SEM (shadow). A black line underneath indicates a 5 s odor. Bar graphs display normalized area under the curve as means ± SEM. Individual data points are displayed as dots, and paired measurements are connected by stippled lines. Asterisks denote a significant difference between averaged responses to CS+ and CS−. See Figure S3 for non-normalized traces for the CS+, CS−, and third odor and Table S1 for statistics.

Defective performance could be specifically attributed to CS− memory. Performance was unaffected when flies were tested with CS+ versus a novel odor (Figure 5B), whereas no performance was evident when they were tested with CS− versus a novel odor (Figure 5C). Blocking PAM-γ3 DANs revealed a surprisingly CS− specific defect. LTM performance was not significantly impaired when flies were tested with CS+ versus CS− (Figure 5A) or CS+ versus a novel odor (Figure 5B). However, when PAM-γ3-blocked flies were tested with CS− versus a novel odor, no CS− approach was observed (Figure 5C). We confirmed the validity of all GAL4; UAS-*Shi*^{ts1} combinations that were observed to impair memory in the DAN screen (Figure 5) by retesting these flies alongside their respective UAS- and GAL4 driver controls at both restrictive (Figures S5A–S5G)

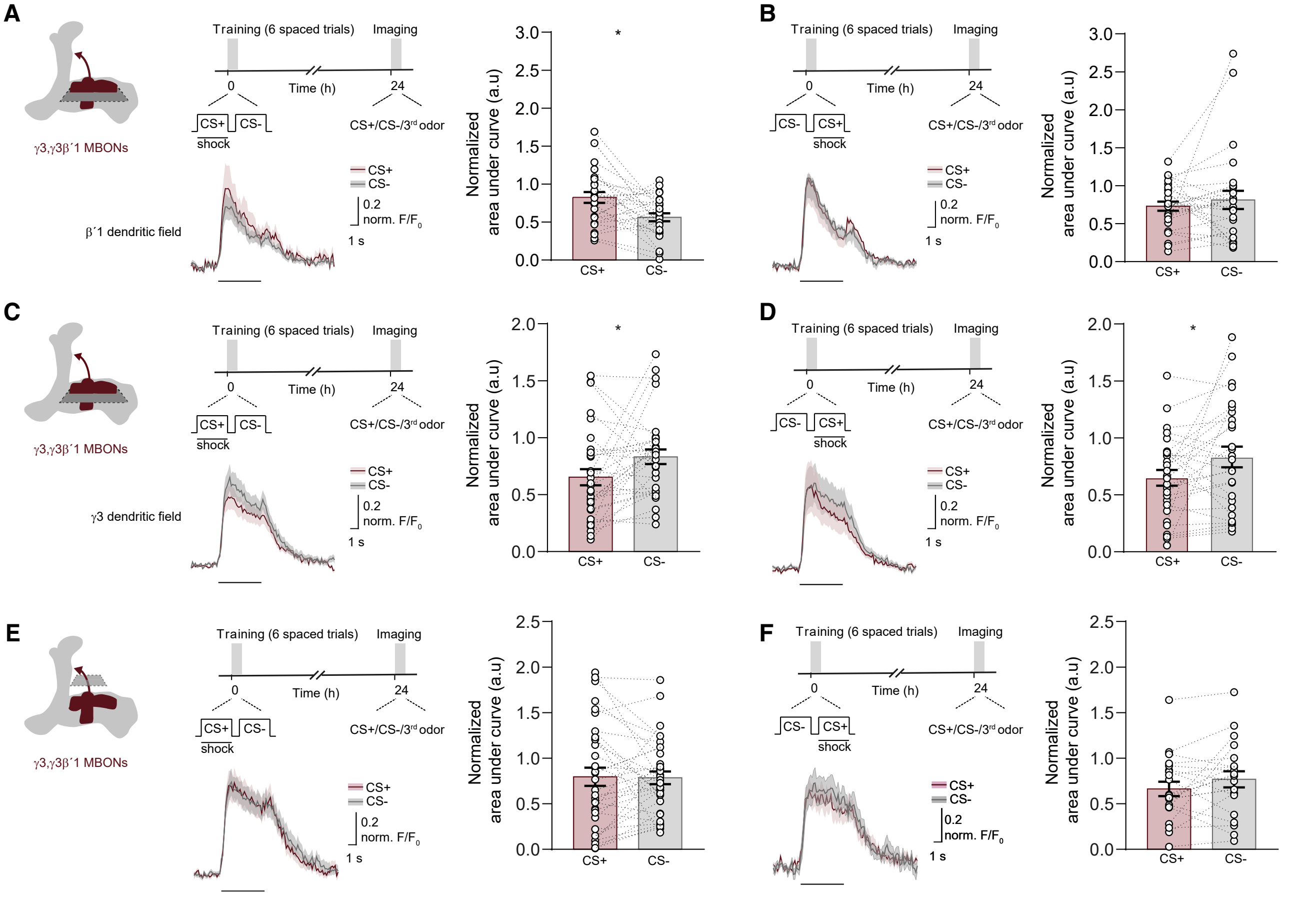

Figure 4. Spaced Training Induces Region-Specific Plasticity of γ3,γ3β′1-MBON Responses

(A–F) Measuring odor responses in γ3,γ3β′1-MBONs. (A and B) Imaging plane for the β′1 region of γ3β′1 MBON dendritic field and training and imaging protocol. (A) Spaced training significantly reduced CS− responses in β′1, but not with (B) the reversed protocol where CS− precedes CS+ in each trial. (C and D). Imaging plane for the γ3 region of the γ3,γ3β′1-MBON dendritic fields and training and imaging protocols. (C) Spaced training significantly reduced CS+ responses in γ3. (D) The reversed protocol also reduced responses to CS+ in γ3. (E and F) Imaging plane in presynaptic terminals of γ3,γ3β′1-MBONs and training and imaging protocols. Neither spaced training (E) or reversed spaced training (F) significantly altered odor-evoked responses in the γ3,γ3β′1 presynaptic terminals. CS+ data correspond to average of experiments in which 50% of trials used MCH as CS+ and 50% used OCT as CS+. Same applies for CS− data. Odor-evoked activity traces show means (solid line) with SEM (shadow). A black line underneath indicates a 5 s odor. Bar graphs display normalized area under the curve as means ± SEM. Individual data points are displayed as dots, and paired measurements are connected by stippled lines. Asterisks denote a significant difference between averaged responses to CS+ and CS−. See Figure S4 for non-normalized traces for the CS+, CS−, and third odor and Table S1 for statistics.

and permissive temperatures (Figures S5H–S5N). Together, these manipulations demonstrate roles for PPL1 DANs in driving plasticity that represents the aversive CS+ memory and for the PAM-β′2mp, PAM-β′1, and PAM-γ3 DANs in coding CS− approach memory.

β′2mp and β′1 DANs Become More Responsive to the CS– Odor after Training Trials

Forming CS− approach memory required output from PAM-γ3, PAM-β′2mp, and PAM-β′1 DANs during training (Figures 2, 5, and S2). We therefore used *in vivo* calcium imaging to test whether these DANs exhibited activity that was consistent with roles in reinforcing CS+ avoidance and CS− approach memories before, during, and after training. The training paradigm employed for imaging was identical to that used for behavioral experiments. DAN responses to a 5 s presentation of CS+ and CS− were measured before and after training, and activity was also monitored during the spaced-training schedule (Figures 6 and S6).

The γ3 DANs responded strongly to electric shocks (Figures 6A, 6C, and S6A–S6D), consistent with their reinforcement of the depression of responses to CS+ in the γ3 arbor of MBON-γ3β′1. Since the shock-evoked activity selectively increases CS+ traces, measuring and comparing responses to CS+ and CS− before and after training without the interference of shock provided a clearer indication of learning-induced changes in DAN activity. After spaced training, the γ3 DANs exhibited a larger response to CS+ than before training (Figure 6B). A similar increase in responses to CS+ was also observed after reversed spaced training (Figure 6D), consistent with the order independence of the learning-induced decrease in the responses to CS+ of the γ3 region of γ3,γ3β′1-MBONs (Figures 4C and 4D).

CellPress
OPEN ACCESS

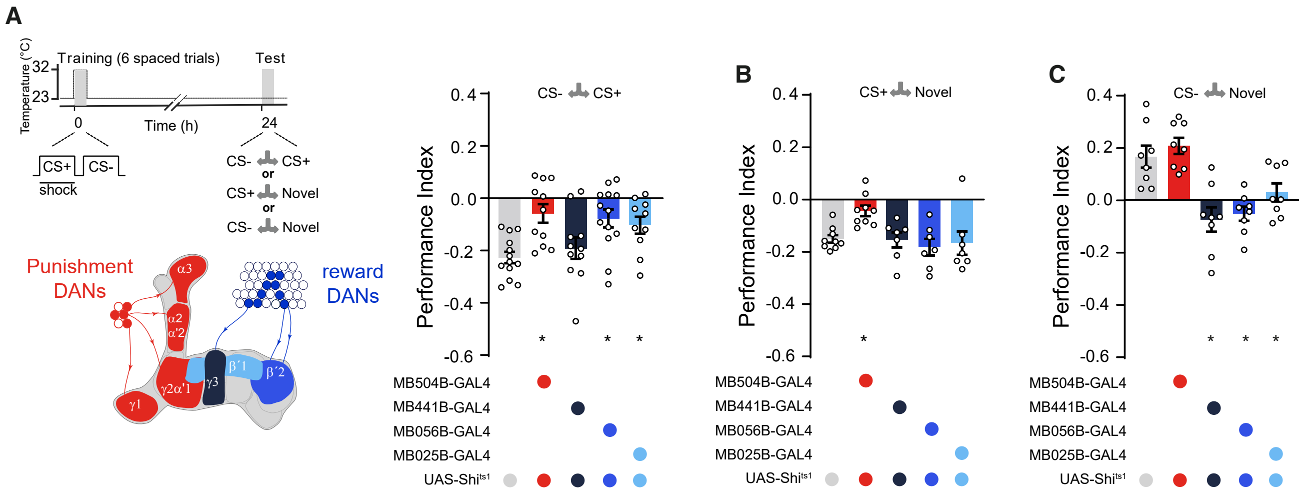

Figure 5. Blocking Specific Dopaminergic Neurons during Spaced Training Localizes Discrete Sites of Aversive and Safety Memory

(A) Left: protocol with temperature shifting (dashed line) and color-coded illustration of DANs labeled with each GAL4. Right: blocking specific DANs impaired LTM. Blocking PPL1-DANs during spaced training with MB504B-GAL4; UAS-*Shi*^{ts1} impaired 24 h memory. Performance was similarly impaired with PAM-β′2mp (MB056B-GAL4) or PAM-β′1 (MB025B-GAL4) block. Blocking PAM-γ3 (MB441B-GAL4) DANs did not impair 24 h performance.

(B) Testing flies' preference between CS+ and a novel odor revealed significant impairment with PPL1-DAN block but not with PAM-β′2mp, PAM-β′1, or PAM-γ3.

(C) CS− memory was impaired when individually blocking PAM-β′2mp, PAM-β′1, and PAM-γ3, whereas blocking PPL1 DANs had no effect. Asterisks denote significant differences. Data are mean ± SEM. Individual data points displayed as dots. See also Figure S5 and Table S1 for statistics.

The β′1 DANs did not respond to electric shocks, but their responses to CS+ decreased as spaced training proceeded, and these responses became significantly different from responses to CS− by the third trial (Figures 6E, S6E, and S6F). As a result, responses to CS+ after training were significantly decreased in comparison to those from beforehand and to responses to CS− (Figure 6F). No differences were observed between odors during training with the reversed spaced protocol (Figures 6G, 6H, S6G, and S6H). However, CS+ and CS− responses were both reduced after reversed spaced training, as compared to their before-training responses (Figure 6H). Therefore, CS− evokes a relatively higher β′1 DAN response only after regular spaced training, suggesting that these DANs reinforce CS− approach memory within the β′1 arbor of the γ3β′1 MBON dendrite.

The β′2mp DANs initially responded in a similar way to both CS+ and CS− odors. However, during spaced training the CS− response gradually increased relative to that of CS+; the difference between responses reached significance by the sixth trial (Figures 6I, S6I, and S6J). β′2mp DANs also showed a clear response to electric shocks delivered during each 1 min of CS+ exposure (Figures 6I, S6I, and S6J). Comparing responses to CS+ and CS− before and after training without the interference of shock confirmed the observation that β′2mp DANs displayed an elevated response to CS− after training, whereas responses to CS+ did not change (Figure 6J). When flies were reverse spaced trained, no differences emerged between responses to CS+ and CS− during (Figures 6K, S6K, and S6L) or after training (Figure 6L). These data support a model wherein β′2mp DANs develop the ability to reinforce CS− approach memory across repeated trials.

DISCUSSION

The gain in memory performance obtained from spacing learning sessions has intrigued scientists for over a century. Early work using *Drosophila* demonstrated that spaced training produced protein-synthesis-dependent 'aversive LTM', whereas massed training did not (Tully et al., 1994; Isabel et al., 2004). Many subsequent studies have compared memory after spaced training to that following massed training. We found that flies learn additional safety information for the CS− odor when subjected to spaced training. Parallel complementary CS+ aversive and CS− approach memories therefore account for the discriminative odor preference observed 24 h after differential spaced training. In contrast, flies only form an avoidance memory for the shock-paired odor when they are mass trained. To our surprise, *rad* mutant flies did not form CS+ aversive memory after spaced training, yet their CS− memory appeared unaffected. In contrast, CXM feeding abolished CS− memory, but CS+ memory was not significantly reduced. If we use previous operational definitions (Tully et al., 1994), these data suggest that CS− memory is protein-synthesis-dependent LTM, whereas the CS+ component is ARM. It is therefore important to rethink the many prior studies that have assumed they were measuring only avoidance of CS+ after spaced training.

Recording a timeline of performance after spaced training revealed that CS+ avoidance and CS− approach memories have a very different dynamic. The CS+ avoidance memory was evident immediately after training, rapidly decayed over 24 h, and was absent at 4 days. In stark contrast, CS− approach memory emerged slowly after training and lasted for at least 4 days—a trajectory reminiscent of that of long-term appetitive memory reinforced by nutritious sugar (Das et al., 2014). The discovery that

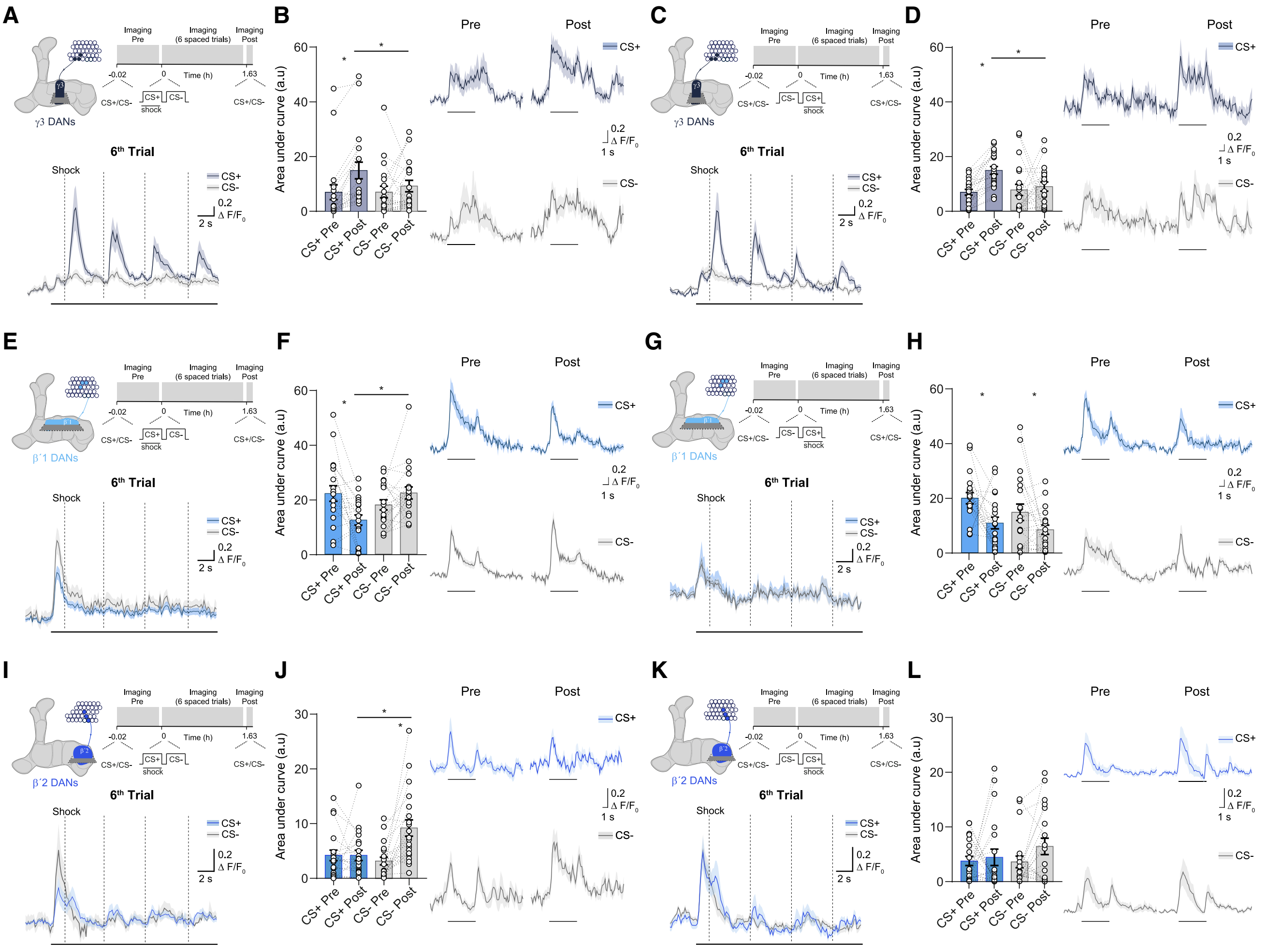

Figure 6. Spaced Training Enhanced CS+ Responses in Aversive γ3 DANs and CS– Responses in Rewarding β′1 and β′2mp DANs

(A) Top: imaging plane in the presynaptic field of γ3 DANs and training and imaging protocol. Bottom: first 20 s of odor-evoked activity from the sixth training trial; shown are CS+ and CS− odor-onset responses masked by strong responses to electric shocks (vertical dashed lines) in the CS+ trace. A horizontal line underneath represents odor presentation. (B) Spaced training changed odor-evoked responses. CS+ responses were elevated in comparison to post-training (post) CS− responses and in comparison to pretraining (pre) CS+ responses.

(C) Top: imaging plane in the presynaptic field of γ3 DANs and training and imaging protocol. Bottom: first 20 s of odor-evoked activity from the sixth training trial; shown are CS+ and CS− odor-onset responses masked by strong responses to electric shocks (vertical dashed lines) in the CS+ trace. The horizontal line underneath represents odor presentation. (D) Reversed spaced training changed odor-evoked responses. CS+ responses were elevated in comparison to post-training CS− responses and in comparison to pre-training CS+ responses.

(E) Top: imaging plane in the presynaptic field of β′1 DANs and training and imaging protocol. Bottom: the first 20 s of odor-evoked activity from the sixth training trial shows strong CS+ and CS− odor-onset responses. A horizontal line marks odor presentation, and vertical dashed lines mark shock delivery.

(F) Spaced training changed odor-evoked responses. Post-training CS− responses were increased relative to responses to CS+. This difference results from a decreased post-training response to CS+.

(G) Reversed spaced training; CS+ and CS− traces were similar during training.

(H) Post-training responses to CS+ and CS− were reduced in comparison to pre-training responses.

(I) Top: imaging plane in the presynaptic field of β′2mp DANs and training and imaging protocol. Bottom: the first 20 s of odor-evoked activity from the sixth training trial shows odor-onset responses for CS+ and CS−. CS+ traces also show rhythmic calcium responses corresponding to electric-shock delivery (vertical dashed lines). A horizontal line underneath marks odor presentation.

(J) A difference between pre-training and post-training responses to CS+ and CS− was observed. Post-training responses evoked by CS− were increased in comparison to those evoked by CS+ and the pretraining responses to CS−.

(K and L) Reversed spaced training. No significant differences were observed (K) between responses to CS+ and CS− during training or (L) between pre-training and post-training odor-evoked responses. Averaged pre- and post-traces are shown alongside all quantifications. A horizontal line marks odor presentation. CS+ data correspond to the average, in which 50% of trials used MCH as CS+ and 50% used OCT as CS+. The same applies for CS− data. Odor-evoked activity traces show means (solid line) with SEM (shadow). Bar graphs display area under the curve as means ± SEM. Individual data points are displayed as dots, and paired measurements are connected by stippled lines. Asterisks denote a significant difference between averaged responses to CS+ and CS−. See also Figure S6 and Table S1 for statistics.

CellPress
OPEN ACCESS

the processes underlying CS+ (ARM) and CS− (LTM) memories have different timing, and different anatomical locations, gives the previously reported mechanistic differences an entirely new perspective. Our data suggest that, rather than occurring in the same neurons, ARM and LTM represent each of the odors employed in differential spaced training. They are therefore likely to be represented in unique populations of odor-activated KCs. In addition, different DANs reinforce CS+ ARM and CS− LTM at different KC-MBON junctions. It follows that, after spaced training, processing of CS+ ARM, which includes the mushroom body-enriched *rad* encoded Rap GAP (Folkers et al., 2006), will occur in different KCs and at different KC locations and output synapses than do the molecular mechanisms that underlie protein-synthesis-dependent CS− LTM.

Relief or Safety Memory?

The valence of olfactory memories can be reversed from aversive to appetitive if the relative timing of odor and reinforcement is altered during training (Tanimoto et al., 2004; König et al., 2018). If shock, or artificial DAN activation, is presented $\leq$45 s before the odor, flies form an appetitive relief memory for that odor (Tanimoto et al., 2004; Aso and Rubin, 2016; Handler et al., 2019). Experiments with artificial DAN activation suggest that relief learning is represented by dopamine potentiating an MBON's response to the conditioned odor (Handler et al., 2019; although see König et al., 2018). If spaced training utilized the same relief-from-punishment mechanism as that in Handler et al. (2019), CS− approach memory would be coded as potentiation of the same connections as those coding CS+ avoidance as a depression. However, we observed co-existence of aversive and approach memories at different places in the MBON network. Our data instead indicate that CS− approach is coded by specific appetitively reinforcing DANs that direct depression of KC outputs onto corresponding MBONs. We also explicitly tested whether spaced relief training could form an equivalent long-term CS− approach memory. These experiments demonstrated that the memory formed differs greatly from that formed after differential spaced training. Most importantly, memory after spaced relief training can be measured immediately but does not persist for 24 h. CS− memory after spaced training emerges slowly and persists for at least 4 days. We therefore propose that CS− approach after spaced training reflects a safety memory for the CS−, rather than that the CS− has been associated with the cessation of punishment. Relief and safety learning are also different in rodents (Mohammadi et al., 2014). We propose that the reason massed training does not form CS− approach memory is that it lacks a period of safety after each CS− presentation.

Different MBONs Guide CS+ and CS– Performance after Spaced Training

Aversive LTM performance, after spaced training, is largely considered to rely on αβ KCs (Isabel et al., 2004) and to be retrieved via α2sc (MB-V2) MBONs (Séjourné et al., 2011; Bouzaiane et al., 2015). However, others have indicated that the network properties are more distributed and that output from γ3,γ3β′1- and α3−MBONs is required to retrieve aversive LTM (Pai et al., 2013; Wu et al., 2017). Our work here suggests that there are different reasons why blocking these MBONs during testing impairs 24 h memory after spaced training. Consistent with prior work (Séjourné et al., 2011), we recorded depressed responses to CS+ in α2sc-MBONs 24 h after spaced training. Depression of α2sc-MBON responses is therefore critical if flies are to express CS+ avoidance. We also observed strong depression of MBON-α3 CS+ responses after spaced training. The role for α3 MBONs has been disputed (Pai et al., 2013; Plaçais et al., 2013). At this point we cannot reconcile differences between the studies, other than perhaps the number of training trials, strength of reinforcement, and relative hunger state of the flies, some of which was proposed by Plaçais et al. (2013). We also note that many recent studies use robots where flies remain in the same tube for the entire training session. In contrast, earlier studies and our experiments reported here utilized manual training where flies are transferred from the training chamber between trials. Nevertheless, our data here suggest that α2sc- and α3-MBONs house plasticity relevant for expression of CS+ aversive memory.

Bouzaiane et al., (2015) reported that MBON-β′2mp and MBON-γ5β′2a (M4/6) are not required for LTM retrieval after spaced training. However, we found that appropriately ordered CS+/CS− spaced trials depressed responses to CS− in β′2mp-MBONs. In addition, we found that PAM-β′2mp DANs are required for the formation of CS− approach memory. Our results therefore indicate a specific role for the β′2mp subcompartment of the β′2 MB zone and that MBON-β′2mp plasticity is required to express CS− approach memory.

The negative sign of odor response plasticity of α2sc-, α3-, and β′2mp-MBONs makes intuitive sense with the known valence of these pathways (Aso et al., 2014a; Owald et al., 2015). Responses to CS+ in approach-directing α2sc- and α3-MBONs were depressed, which would favor odor avoidance. In contrast, depressing responses to CS− to avoidance-directing β′2mp-MBONs should promote odor approach.

γ3β′1 MBONs Compute and Provide a Measure of Relative Safety?

We also discovered roles for PAM-γ3 and PAM-β′1 DANs and recorded traces of both CS+ and CS− memory in the corresponding γ3,γ3β′1-MBONs. MBON dendrites in the γ3 compartment showed a decreased response to the CS+, irrespective of the order of CS+ and CS− in the training trials, consistent with the rules of forming aversive CS+ memory. In contrast, CS− responses were decreased in the β′1 tuft of γ3β′1 MBON dendrites, but only if flies were trained with CS+ and then CS−, in that order. Plasticity in β′1 of the γ3β′1 MBON therefore followed the order rule observed for conditioning CS− approach behavior. Interestingly, recording in the axons of γ3 and γ3β′1 MBONs suggested that CS+ and CS− plasticity cancel each other out. Unfortunately, the split-GAL4 used for driving GCaMP expression in γ3β′1 MBONs also labels γ3 MBONs. Therefore, although only the γ3β′1 MBONs have a dendrite in both γ3 and β′1 compartments, we cannot at this stage be certain that γ3β′1 MBONs alone integrate CS+ and CS− memory traces.

To decipher the relative role of γ3 and β′1 plasticity, we individually blocked output from PAM-γ3 and PAM-β′1 DANs during training and tested the resulting memories. Behavioral

observations after the PAM-γ3 block were particularly revealing. PAM-γ3 DANs respond to shock (this study and Cohn et al., 2015), and their forced activation reinforces aversive memories (Yamagata et al., 2016). However, blocking PAM-γ3 DANs during spaced training did not impair CS+ avoidance and instead impaired CS− approach when flies were tested with CS− versus a novel odor. A CS− memory defect was also observed when the appetitively reinforcing PAM β′1 DANs were blocked during training, although this manipulation also impaired CS+ versus CS− performance. Lastly, blocking the γ3,γ3β′1-MBONs during testing selectively impaired expression of CS− but not CS+ memory. We therefore propose that γ3β′1 MBONs integrate the γ3 CS+ danger and β′1 CS− safety plasticity to compute a relative safety signal. The importance of this is only obvious if we block the γ3,γ3β′1-MBONs during testing or remove aversive CS+ plasticity in γ3 and thereby reveal the behavioral consequence of unopposed CS− plasticity in the β′1 region of γ3β′1 MBONs. Since MBON-γ3 and MBON-γ3β′1 are GABAergic (Aso et al., 2014a), spaced training sequentially alters the level of CS+ and CS− driven inhibition that is imposed on their downstream target neurons.

A Subset of Dopaminergic Neurons Code Learned Safety

Our results here demonstrate that DANs reinforce the delayed recognition of safety. Formation of CS− approach memory requires appetitively reinforcing PAM-β′2mp and PAM-β′1 DANs and, surprisingly, aversively reinforcing PAM-γ3 DANs. As noted above, PAM-γ3 DANs most likely provide an aversive teaching signal (Yamagata et al., 2016) that directs CS+ plasticity in the γ3 region of MBON-γ3β′1 dendrites. Blocking output from PAM-β′2mp, PAM-β′1, or PAM-γ3 DANs, which are presumably responsible for each part of the LTM-correlated plasticity, reveals they are required for the formation of CS− approach memories during training. Blocking most PAM DANs further localized an essential role during CS− presentation in each spaced training trial, suggesting that safety-memory formation is driven by CS− odor. However, safety-memory formation also requires that each CS+ exposure precedes each CS− exposure in each training trial. Therefore, PAM DANs also have to somehow register a temporally locked negatively reinforced CS+ reference to be able to classify the following CS− as safe. Lastly, repetition is a necessary element of triggering DANs to code safety. Our imaging of the activity of appetitively reinforcing PAM-β′2mp and PAM-β′1 DANs during and after training suggests they gradually acquire the capacity to reinforce CS− approach memory across differential spaced-training trial repetitions. Both PAM-β′2mp and PAM-β′1 DANs exhibited an increased activation by CS− odor, relative to the CS+, over consecutive training trials, and this difference was particularly clear when we compared activity after the sixth training trial to activity before training. In addition, the shock responsiveness of PAM-β′2mp appeared to diminish over time. We propose that over repetitive trials the CS− odor becomes the trigger that activates PAM-β′2mp and PAM-β′1 DANs.

It is conceivable that formation of long-term CS+ and CS− memories is orchestrated by aversive reinforcement signals provided by the PPL1-γ1pedc (MP1) and PPL1-γ2α′1 (MV1) DANs in each shock-paired CS+ trial (Claridge-Chang et al., 2009; Aso et al., 2010, 2014a; Plaçais et al., 2012; Aso and Rubin, 2016). PPL1-γ1pedc DANs code aversive learning by depressing odor-specific input to feedforward GABAergic γ1pedc>αβ (MVP2) MBONs (Hige et al., 2015; Perisse et al., 2016). Although MVP2 output is only required for the expression of short-term aversive memory, the plasticity remains for several hours (Perisse et al., 2016). Each shock-reinforced odor trial therefore changes the state of the rest of the MBON and DAN network for subsequent exposures and reinforced trials. This has been proposed to release PPL1-α′2α2 DANs so they can reinforce LTM at the KC-MBON-α2sc junction (Awata et al., 2019). A similar release from inhibition of PPL1-α3, PAM-β′2mp, and PAM-β′1 DANs could account for our spaced-training-driven plasticity at MBON-α3 and prime the PAM-β′2mp and PAM-β′1 to reinforce the CS− memory.

However, our data instead suggest that plasticity of the GABAergic γ3β′1 MBONs is essential for the formation of safety memory. Whereas blocking all PPL1 DANs abolished CS+ memory, CS− memory was unaffected by this manipulation. In contrast, blocking shock-activated PAM-γ3 DANs during training selectively impaired the formation of CS− memory. We therefore propose that spaced-training-evoked PAM-γ3 DAN activity cumulatively depresses CS+ driven activity of γ3β′1 MBONs, and this releases the PAM-β′1 and PAM-β′2mp DANs from inhibition to reinforce CS− memory. Such a model potentially explains the required relationship between CS+ and CS− memories, the need for trial repetition, and the relative increase in the responses of these DANs to CS− with each training trial. Although our results do not provide an explanation for the optimal 15 min ITI (or proposed period of safety), prior studies have suggested that protein-synthesis-dependent LTM formation requires the timing of consecutive spaced training trials to coincide with the peak of training-induced MAPK activity in KCs (Pagani et al., 2009; Miyashita et al., 2018).

Reinforcing PAM DANs have also been implicated in memory formation with sugar (Burke et al., 2012; Liu et al., 2012), water (Lin et al., 2014; Shyu et al., 2017), and alcohol reward (Scaplen et al., 2019), with relative shock (Perisse et al., 2013), with the absence of expected shock (Felsenberg et al., 2018), and after courtship (Keleman et al., 2012). In addition, they provide control of state-dependent memory expression (Senapati et al., 2019) and unlearned behavioral responses to volatile cues (Lin et al., 2014; Lewis et al., 2015). In some cases, these processes clearly involve different DANs, whereas in others they appear to involve DANs that innervate the same MB compartments. More refined tools, connectomics (Zheng et al., 2018; Otto et al., 2020), and experiments should help reveal the full extent of functional heterogeneity.

Ubiquity and Utility of Parallel Memories

Fly behavior has previously been shown to depend on the addition of supporting or conflicting experience. When differentially conditioned by the pairing of one odor with shock and the other with sugar, flies show additive initial performance compared to that observed if only one of the two odors is reinforced (Tempel et al., 1983). This situation resembles that described here after

CellPress
OPEN ACCESS

spaced training except that the second odor is explicitly unpaired, and additive performance emerges from complementary LTM. With the benefit of retrospect, and as discussed before (Schleyer et al., 2018), it makes intuitive sense that over repetitive spaced trials flies learn "where the punishment is and where it is not." These parallel memories make it easier for flies to distinguish between the two odors when tested together (Barth et al., 2014).

In contrast, flies simultaneously form parallel competing memories when trained with bitter-tainted sugar, and their performance switches from aversion to approach over time, as dictated by the superior persistence of the nutrient-dependent sugar memory (Das et al., 2014). A similar time-dependent behavioral transition is evident when flies are trained with alcohol reinforcement (Kaun et al., 2011). A competition between memories of opposing valence also underlies the extinction of both appetitive and aversive memories (Felsenberg et al., 2017, 2018). However, opposing extinction memories are sequentially formed and are reinforced by the absence of an expected outcome, rather than explicit pairing. In these cases, forming parallel memories reduces the certainty of odor choice.

Together, these studies suggest that forming parallel memories in different places is a general MBON network feature that allows flies to summate experience over time to optimize the expression of learned behavior.

STAR★METHODS

Detailed methods are provided in the online version of this paper and include the following:

- KEY RESOURCES TABLE
- RESOURCE AVAILABILITY
  - Lead Contact and Materials Availability
- DATA AND CODE AVAILABILITY
- EXPERIMENTAL MODEL AND SUBJECT DETAILS
  - Fly strains
- METHOD DETAILS
  - Behavioral experiments
  - CXM feeding
  - Two-Photon Calcium Imaging
- QUANTIFICATION AND STATISTICAL ANALYSIS

SUPPLEMENTAL INFORMATION

Supplemental Information can be found online at https://doi.org/10.1016/j.neuron.2020.03.013.

ACKNOWLEDGMENTS

We thank Tim Tully and Steve de Belle for comments on the preprint version of this manuscript. We are grateful to J. Felsenberg, V. Croset, E. Perisse, G. Wright, and other members of the Waddell group for discussion. We thank G. Rubin, FlyLight, B. Dickson, and the Bloomington Stock Center for flies. S.W. was funded by a Wellcome Principal Research Fellowship (200846/Z/16/Z), Gatsby Charitable Foundation (GAT3237), ERC Advanced Grant (789274), and the Bettencourt–Schueller Foundation.

AUTHOR CONTRIBUTIONS

Conceptualization, P. F. J. and S.W.; Methodology, P.F.J. and S.W.; Investigation, P.F.J.; Resources, S.W.; Writing, S.W. and P.F.J.; Supervision, S.W.; Funding Acquisition, S.W.

DECLARATION OF INTERESTS

The authors declare no competing interests.

Received: September 27, 2019
Revised: January 25, 2020
Accepted: March 16, 2020
Published: April 13, 2020

REFERENCES

Akalal, D.-B.G., Yu, D., and Davis, R.L. (2010). A late-phase, long-term memory trace forms in the γ neurons of Drosophila mushroom bodies after olfactory classical conditioning. J. Neurosci. *30*, 16699–16708.

Aso, Y., and Rubin, G.M. (2016). Dopaminergic neurons write and update memories with cell-type-specific rules. eLife *5*, 1–15.

Aso, Y., Siwanowicz, I., Bräcker, L., Ito, K., Kitamoto, T., and Tanimoto, H. (2010). Specific dopaminergic neurons for the formation of labile aversive memory. Curr. Biol. *20*, 1445–1451.

Aso, Y., Sitaraman, D., Ichinose, T., Kaun, K.R., Vogt, K., Belliart-Guérin, G., Plaçais, P.-Y.Y., Robie, A.A., Yamagata, N., Schnaitmann, C., et al. (2014a). Mushroom body output neurons encode valence and guide memory-based action selection in Drosophila. eLife *3*, e04580.

Aso, Y., Hattori, D., Yu, Y., Johnston, R.M., Iyer, N.A., Ngo, T.-T.T.B., Dionne, H., Abbott, L.F.L., Axel, R., Tanimoto, H., and Rubin, G.M. (2014b). The neuronal architecture of the mushroom body provides a logic for associative learning. eLife *3*, e04577.

Awata, H., Takakura, M., Kimura, Y., Iwata, I., Masuda, T., and Hirano, Y. (2019). The neural circuit linking mushroom body parallel circuits induces memory consolidation in *Drosophila*. Proc. Natl. Acad. Sci. USA *116*, 16080–16085.

Barth, J., Dipt, S., Pech, U., Hermann, M., Riemensperger, T., and Fiala, A. (2014). Differential associative training enhances olfactory acuity in Drosophila melanogaster. J. Neurosci. *34*, 1819–1837.

Bartsch, D., Casadio, A., Karl, K.A., Serodio, P., and Kandel, E.R. (1998). CREB1 encodes a nuclear activator, a repressor, and a cytoplasmic modulator that form a regulatory unit critical for long-term facilitation. Cell *95*, 211–223.

Boto, T., Louis, T., Jindachomthong, K., Jalink, K., and Tomchik, S.M. (2014). Dopaminergic modulation of cAMP drives nonlinear plasticity across the Drosophila mushroom body lobes. Curr. Biol. *24*, 822–831.

Bouzaiane, E., Trannoy, S., Scheunemann, L., Plaçais, P.Y., and Preat, T. (2015). Two independent mushroom body output circuits retrieve the six discrete components of Drosophila aversive memory. Cell Rep. *11*, 1280–1292.

Burke, C.J., Huetteroth, W., Owald, D., Perisse, E., Krashes, M.J., Das, G., Gohl, D., Silies, M., Certel, S., and Waddell, S. (2012). Layered reward signalling through octopamine and dopamine in Drosophila. Nature *492*, 433–437.

Carew, T.J., Pinsker, H.M., and Kandel, E.R. (1972). Long-term habituation of a defensive withdrawal reflex in aplysia. Science *175*, 451–454.

Chen, C.C., Wu, J.K., Lin, H.W., Pai, T.P., Fu, T.F., Wu, C.L., Tully, T., and Chiang, A.S. (2012). Visualizing long-term memory formation in two neurons of the Drosophila brain. Science *335*, 678–685.

Chen, T.-W., Wardill, T.J., Sun, Y., Pulver, S.R., Renninger, S.L., Baohan, A., Schreiter, E.R., Kerr, R.A., Orger, M.B., Jayaraman, V., et al. (2013). Ultrasensitive fluorescent proteins for imaging neuronal activity. Nature *499*, 295–300.

Claridge-Chang, A., Roorda, R.D., Vrontou, E., Sjulson, L., Li, H., Hirsh, J., and Miesenböck, G. (2009). Writing memories with light-addressable reinforcement circuitry. Cell *139*, 405–415.

Cohn, R., Morantte, I., and Ruta, V. (2015). Coordinated and Compartmentalized Neuromodulation Shapes Sensory Processing in Drosophila. Cell *163*, 1742–1755.

Das, G., Klappenbach, M., Vrontou, E., Perisse, E., Clark, C.M., Burke, C.J., and Waddell, S. (2014). Drosophila learn opposing components of a compound food stimulus. Curr. Biol. *24*, 1723–1730.

Dubnau, J., Chiang, A.S., Grady, L., Barditch, J., Gossweiler, S., McNeil, J., Smith, P., Buldoc, F., Scott, R., Certa, U., et al. (2003). The staufen/pumilio pathway is involved in Drosophila long-term memory. Curr. Biol. *13*, 286–296.

Ebbinghaus, H. (1913). Memory: A contribution to experimental psychology, H.A. Ruger and C.E. Bussenius, eds. (New York: Teachers College Press), trans.

Felsenberg, J., Barnstedt, O., Cognigni, P., Lin, S., and Waddell, S. (2017). Re-evaluation of learned information in Drosophila. Nature *544*, 240–244.

Felsenberg, J., Jacob, P.F., Walker, T., Barnstedt, O., Edmondson-Stait, A.J., Pleijzier, M.W., Otto, N., Schlegel, P., Sharifi, N., Perisse, E., et al. (2018). Integration of Parallel Opposing Memories Underlies Memory Extinction. Cell *175*, 709–722.e15.

Folkers, E., Drain, P., and Quinn, W.G. (1993). Radish, a Drosophila mutant deficient in consolidated memory. Proc. Natl. Acad. Sci. USA *90*, 8123–8127.

Folkers, E., Waddell, S., and Quinn, W.G. (2006). The Drosophila radish gene encodes a protein required for anesthesia-resistant memory. Proc. Natl. Acad. Sci. USA *103*, 17496–17500.

Han, J.-H., Kushner, S.A., Yiu, A.P., Cole, C.J., Matynia, A., Brown, R.A., Neve, R.L., Guzowski, J.F., Silva, A.J., and Josselyn, S.A. (2007). Neuronal competition and selection during memory formation. Science *316*, 457–460.

Handler, A., Graham, T.G.W., Cohn, R., Morantte, I., Siliciano, A.F., Zeng, J., Li, Y., and Ruta, V. (2019). Distinct Dopamine Receptor Pathways Underlie the Temporal Sensitivity of Associative Learning. Cell *178*, 60–75.e19.

Hermitte, G., Pedreira, M.E., Tomsic, D., and Maldonado, H. (1999). Context shift and protein synthesis inhibition disrupt long-term habituation after spaced, but not massed, training in the crab Chasmagnathus. Neurobiol. Learn. Mem. *71*, 34–49.

Hige, T., Aso, Y., Modi, M.N., Rubin, G.M., and Turner, G.C. (2015). Heterosynaptic Plasticity Underlies Aversive Olfactory Learning in Drosophila. Neuron *88*, 985–998.

Hirano, Y., Masuda, T., Naganos, S., Matsuno, M., Ueno, K., Miyashita, T., Horiuchi, J., and Saitoe, M. (2013). Fasting launches CRTC to facilitate long-term memory formation in Drosophila. Science *339*, 443–446.

Huang, C., Zheng, X., Zhao, H., Li, M., Wang, P., Xie, Z., Wang, L., and Zhong, Y. (2012). A permissive role of mushroom body α/β core neurons in long-term memory consolidation in Drosophila. Curr. Biol. *22*, 1981–1989.

Impey, S., Obrietan, K., Wong, S.T., Poser, S., Yano, S., Wayman, G., Deloulme, J.C., Chan, G., and Storm, D.R. (1998). Cross talk between ERK and PKA is required for Ca2+ stimulation of CREB-dependent transcription and ERK nuclear translocation. Neuron *21*, 869–883.

Isabel, G., Pascual, A., and Preat, T. (2004). Exclusive consolidated memory phases in Drosophila. Science *304*, 1024–1027.

Jenett, A., Rubin, G.M., Ngo, T.T.B., Shepherd, D., Murphy, C., Dionne, H., Pfeiffer, B.D., Cavallaro, A., Hall, D., Jeter, J., et al. (2012). A GAL4-driver line resource for Drosophila neurobiology. Cell Rep. *2*, 991–1001.

Kaun, K.R., Azanchi, R., Maung, Z., Hirsh, J., and Heberlein, U. (2011). A Drosophila model for alcohol reward. Nat. Neurosci. *14*, 612–619.

Keleman, K., Vrontou, E., Krüttner, S., Yu, J.Y., Kurtovic-Kozaric, A., and Dickson, B.J. (2012). Dopamine neurons modulate pheromone responses in Drosophila courtship learning. Nature *489*, 145–149.

Kim, Y.-C., Lee, H.-G., and Han, K.-A. (2007). D1 dopamine receptor dDA1 is required in the mushroom body neurons for aversive and appetitive learning in Drosophila. J. Neurosci. *27*, 7640–7647.

Kitamoto, T. (2001). Conditional modification of behavior in Drosophila by targeted expression of a temperature-sensitive shibire allele in defined neurons. J. Neurobiol. *47*, 81–92.

Kogan, J.H., Frankland, P.W., Blendy, J.A., Coblentz, J., Marowitz, Z., Schütz, G., and Silva, A.J. (1997). Spaced training induces normal long-term memory in CREB mutant mice. Curr. Biol. *7*, 1–11.

König, C., Khalili, A., Ganesan, M., Nishu, A.P., Garza, A.P., Niewalda, T., Gerber, B., Aso, Y., and Yarali, A. (2018). Reinforcement signaling of punishment versus relief in fruit flies. Learn. Mem. *25*, 247–257.

Lewis, L.P.C.C., Siju, K.P., Aso, Y., Friedrich, A.B., Bulteel, A.J.B.B., Rubin, G.M., and Grunwald Kadow, I.C. (2015). A Higher Brain Circuit for Immediate Integration of Conflicting Sensory Information in Drosophila. Curr. Biol. *25*, 2203–2214.

Lin, S., Owald, D., Chandra, V., Talbot, C., Huetteroth, W., and Waddell, S. (2014). Neural correlates of water reward in thirsty Drosophila. Nat. Neurosci. *17*, 1536–1542.

Liu, C., Plaçais, P.Y., Yamagata, N., Pfeiffer, B.D., Aso, Y., Friedrich, A.B., Siwanowicz, I., Rubin, G.M., Preat, T., and Tanimoto, H. (2012). A subset of dopamine neurons signals reward for odour memory in Drosophila. Nature *488*, 512–516.

Liu, R.Y., Zhang, Y., Baxter, D.A., Smolen, P., Cleary, L.J., and Byrne, J.H. (2013). Deficit in long-term synaptic plasticity is rescued by a computationally predicted stimulus protocol. J. Neurosci. *33*, 6944–6949.

Masek, P., and Heisenberg, M. (2008). Distinct memories of odor intensity and quality in Drosophila. Proc. Natl. Acad. Sci. USA *105*, 15985–15990.

Menzel, R., Manz, G., Menzel, R., and Greggers, U. (2001). Massed and spaced learning in honeybees: the role of CS, US, the intertrial interval, and the test interval. Learn. Mem. *8*, 198–208.

Miyashita, T., Oda, Y., Horiuchi, J., Yin, J.C.P., Morimoto, T., and Saitoe, M. (2012). Mg(2+) block of Drosophila NMDA receptors is required for long-term memory formation and CREB-dependent gene expression. Neuron *74*, 887–898.

Miyashita, T., Kikuchi, E., Horiuchi, J., and Saitoe, M. (2018). Long-Term Memory Engram Cells Are Established by c-Fos/CREB Transcriptional Cycling. Cell Rep. *25*, 2716–2728.e3.

Mohammad, F., Stewart, J.C., Ott, S., Chlebikova, K., Chua, J.Y., Koh, T.W., Ho, J., and Claridge-Chang, A. (2017). Optogenetic inhibition of behavior with anion channelrhodopsins. Nat. Methods *14*, 271–274.

Mohammadi, M., Bergado-Acosta, J.R., and Fendt, M. (2014). Relief learning is distinguished from safety learning by the requirement of the nucleus accumbens. Behav. Brain Res. *272*, 40–45.

Otto, N., Pleijzier, M.W., Morgan, I.C., Edmondson-Stait, A.J., Heinz, K.J., Stark, I., Dempsey, G., Ito, M., Kapoor, I., Hsu, J., et al. (2020). Input connectivity reveals additional heterogeneity of dopaminergic reinforcement in Drosophila. bioRxiv. https://doi.org/10.1101/2020.02.19.952648.

Owald, D., Felsenberg, J., Talbot, C.B., Das, G., Perisse, E., Huetteroth, W., and Waddell, S. (2015). Activity of defined mushroom body output neurons underlies learned olfactory behavior in Drosophila. Neuron *86*, 417–427.

Pagani, M.R., Oishi, K., Gelb, B.D., and Zhong, Y. (2009). The phosphatase SHP2 regulates the spacing effect for long-term memory induction. Cell *139*, 186–198.

Pai, T.-P., Chen, C.-C., Lin, H.-H., Chin, A.-L., Lai, J.S.-Y., Lee, P.-T., Tully, T., and Chiang, A.-S. (2013). Drosophila ORB protein in two mushroom body output neurons is necessary for long-term memory formation. Proc. Natl. Acad. Sci. USA *110*, 7898–7903.

Park, S., Kramer, E.E., Mercaldo, V., Rashid, A.J., Insel, N., Frankland, P.W., and Josselyn, S.A. (2016). Neuronal Allocation to a Hippocampal Engram. Neuropsychopharmacology *41*, 2987–2993.

Pascual, A., and Préat, T. (2001). Localization of long-term memory within the Drosophila mushroom body. Science *294*, 1115–1117.

Pavlov, I.V. (1927). Conditioned reflexes: An investigation of the physiological activity of the cerebral cortex (London: Oxford University Press).

CellPress
OPEN ACCESS

Perisse, E., Yin, Y., Lin, A.C., Lin, S., Huetteroth, W., and Waddell, S. (2013). Different kenyon cell populations drive learned approach and avoidance in Drosophila. Neuron *79*, 945–956.

Perisse, E., Owald, D., Barnstedt, O., Talbot, C.B.B., Huetteroth, W., and Waddell, S. (2016). Aversive Learning and Appetitive Motivation Toggle Feed-Forward Inhibition in the Drosophila Mushroom Body. Neuron *90*, 1086–1099.

Plaçais, P.-Y., Trannoy, S., Isabel, G., Aso, Y., Siwanowicz, I., Belliart-Guérin, G., Vernier, P., Birman, S., Tanimoto, H., and Preat, T. (2012). Slow oscillations in two pairs of dopaminergic neurons gate long-term memory formation in Drosophila. Nat. Neurosci. *15*, 592–599.

Plaçais, P.-Y., Trannoy, S., Friedrich, A.B., Tanimoto, H., and Preat, T. (2013). Two pairs of mushroom body efferent neurons are required for appetitive long-term memory retrieval in Drosophila. Cell Rep. *5*, 769–780.

Pologruto, T.A., Sabatini, B.L., and Svoboda, K. (2003). ScanImage: flexible software for operating laser scanning microscopes. Biomed. Eng. Online *2*, 13.

Qin, H., Cressy, M., Li, W., Coravos, J.S., Izzi, S.A., and Dubnau, J. (2012). Gamma neurons mediate dopaminergic input during aversive olfactory memory formation in Drosophila. Curr. Biol. *22*, 608–614.

Scaplen, K.M., Talay, M., Salamon, S., Nuñez, K.M., Waterman, A.G., Gang, S., Song, S.L., Barnea, G., and Kaun, K.R. (2019). Circuits that encode and predict alcohol associated preference. bioRxiv. https://doi.org/10.1101/578401.

Schindelin, J., Arganda-Carreras, I., Frise, E., Kaynig, V., Longair, M., Pietzsch, T., Preibisch, S., Rueden, C., Saalfeld, S., Schmid, B., et al. (2012). Fiji: an open-source platform for biological-image analysis. Nat. Methods *9*, 676–682.

Schleyer, M., Fendt, M., Schuller, S., and Gerber, B. (2018). Associative learning of stimuli paired and unpaired with reinforcement: Evaluating evidence from maggots, flies, bees, and rats. Front. Psychol. *9*, 1494.

Séjourné, J., Plaçais, P.-Y., Aso, Y., Siwanowicz, I., Trannoy, S., Thoma, V., Tedjakumala, S.R., Rubin, G.M., Tchénio, P., Ito, K., et al. (2011). Mushroom body efferent neurons responsible for aversive olfactory memory retrieval in Drosophila. Nat. Neurosci. *14*, 903–910.

Senapati, B., Tsao, C.-H., Juan, Y.-A., Chiu, T.H., Wu, C.L., Waddell, S., and Lin, S. (2019). A neural mechanism for deprivation state-specific expression of relevant memories in Drosophila. Nat. Neurosci. *22*, 2029–2039.

Shang, Y., Claridge-Chang, A., Sjulson, L., Pypaert, M., and Miesenböck, G. (2007). Excitatory local circuits and their implications for olfactory processing in the fly antennal lobe. Cell *128*, 601–612.

Shyu, W.H., Chiu, T.H., Chiang, M.H., Cheng, Y.C., Tsai, Y.L., Fu, T.F., Wu, T., and Wu, C.L. (2017). Neural circuits for long-term water-reward memory processing in thirsty Drosophila. Nat. Commun. *8*, 15230.

Smolen, P., Zhang, Y., and Byrne, J.H. (2016). The right time to learn: mechanisms and optimization of spaced learning. Nat. Rev. Neurosci. *17*, 77–88.

Tanaka, N.K., Tanimoto, H., and Ito, K. (2008). Neuronal assemblies of the Drosophila mushroom body. J. Comp. Neurol. *508*, 711–755.

Tanimoto, H., Heisenberg, M., and Gerber, B. (2004). Experimental psychology: event timing turns punishment to reward. Nature *430*, 983.

Tempel, B.L., Bonini, N., Dawson, D.R., and Quinn, W.G. (1983). Reward learning in normal and mutant Drosophila. Proc. Natl. Acad. Sci. USA *80*, 1482–1486.

Thomas, G.M., and Huganir, R.L. (2004). MAPK cascade signalling and synaptic plasticity. Nat. Rev. Neurosci. *5*, 173–183.

Tomchik, S.M., and Davis, R.L. (2009). Dynamics of learning-related cAMP signaling and stimulus integration in the Drosophila olfactory pathway. Neuron *64*, 510–521.

Tully, T., and Quinn, W.G. (1985). Classical conditioning and retention in normal and mutant Drosophila melanogaster. J. Comp. Physiol. A Neuroethol. Sens. Neural Behav. Physiol. *157*, 263–277.

Tully, T., Preat, T., Boynton, S.C., and Del Vecchio, M. (1994). Genetic dissection of consolidated memory in Drosophila. Cell *79*, 35–47.

Wu, J.K., Tai, C.Y., Feng, K.L., Chen, S.L., Chen, C.C., and Chiang, A.S. (2017). Long-term memory requires sequential protein synthesis in three subsets of mushroom body output neurons in Drosophila. Sci. Rep. *7*, 7112.

Yamagata, N., Hiroi, M., Kondo, S., Abe, A., and Tanimoto, H. (2016). Suppression of Dopamine Neurons Mediates Reward. PLoS Biol. *14*, e1002586.

Yin, J.C.P., Wallach, J.S., Del Vecchio, M., Wilder, E.L., Zhou, H., Quinn, W.G., and Tully, T. (1994). Induction of a dominant negative CREB transgene specifically blocks long-term memory in Drosophila. Cell *79*, 49–58.

Yu, D., Akalal, D.-B.G., and Davis, R.L. (2006). Drosophila α/β mushroom body neurons form a branch-specific, long-term cellular memory trace after spaced olfactory conditioning. Neuron *52*, 845–855.

Zhang, S., and Roman, G. (2013). Presynaptic inhibition of gamma lobe neurons is required for olfactory learning in Drosophila. Curr. Biol. *23*, 2519–2527.

Zhang, Y., Liu, R.Y., Heberton, G.A., Smolen, P., Baxter, D.A., Cleary, L.J., and Byrne, J.H. (2012). Computational design of enhanced learning protocols. Nat. Neurosci. *15*, 294–297.

Zheng, Z., Lauritzen, J.S., Perlman, E., Robinson, C.G., Nichols, M., Milkie, D., Torrens, O., Price, J., Fisher, C.B., Sharifi, N., et al. (2018). A Complete Electron Microscopy Volume of the Brain of Adult Drosophila melanogaster. Cell *174*, 730–743.e22.

Zhou, Y., Won, J., Karlsson, M.G., Zhou, M., Rogerson, T., Balaji, J., Neve, R., Poirazi, P., and Silva, A.J. (2009). CREB regulates excitability and the allocation of memory to subsets of neurons in the amygdala. Nat. Neurosci. *12*, 1438–1443.

STAR★METHODS

KEY RESOURCES TABLE

REAGENT or RESOURCE	SOURCE	IDENTIFIER
**Chemicals, Peptides, and Recombinant Proteins**		
N-Tris	Sigma-Aldrich	Cat#T5691
NaCl	Sigma-Aldrich	Cat#S7653
KCl	Sigma-Aldrich	Cat#P9333
$NaHCO_3$	Sigma-Aldrich	Cat#S6297
NaH_2PO_4	Sigma-Aldrich	Cat#S8282
$CaCl_2$	Sigma-Aldrich	Cat#21115
$MgCl_2$	Sigma-Aldrich	Cat#M1028
Trehalose	Sigma-Aldrich	Cat#T9531
Glucose	Sigma-Aldrich	Cat#G7528
Sucrose	Sigma-Aldrich	Cat# S0389
Mineral Oil	Sigma-Aldrich	Cat#M5904
4-methylcyclohexanol (98%)	Sigma-Aldrich	Cat#218405
3-octanol (99%)	Sigma-Aldrich	Cat#153095
Isopentyl acetate (99%)	Sigma-Aldrich	Cat#306967
Cycloheximide	Sigma-Aldrich	Cat#7698
**Experimental Models: Organisms/Strains**		
D. melanogaster: MB110C-Gal4	Bloomington Drosophila Stock Center; Aso et al., 2014a, 2014b	RRID:BDSC_68262
D. melanogaster: R66C08-Gal4	Bloomington Drosophila Stock Center; Owald et al., 2015	RRID:BDSC_49412
D. melanogaster: R39A05-Gal4	Bloomington Drosophila Stock Center; Jenett et al., 2012	RRID:BDSC_50033
D. melanogaster: R71D08-Gal4	Bloomington Drosophila Stock Center; Jenett et al., 2012	RRID:BDSC_61645
D. melanogaster: G0239-Gal4	Bloomington Drosophila Stock Center; Pai et al., 2013	RRID:BDSC_12639
D. melanogaster: R58E02-Gal4	Bloomington Drosophila Stock Center; Liu et al., 2012	RRID:BDSC_41347
D. melanogaster: MB504B-Gal4	Bloomington Drosophila Stock Center; Aso et al., 2014a, 2014b	RRID:BDSC_68329
D. melanogaster: MB056B-Gal4	Bloomington Drosophila Stock Center; Aso et al., 2014a, 2014b	RRID:BDSC_68276
D. melanogaster: MB441B-Gal4	Bloomington Drosophila Stock Center; Aso et al., 2014a, 2014b	RRID:BDSC_68251
D. melanogaster: MB025B-Gal4	Bloomington Drosophila Stock Center; Aso et al., 2014a, 2014b	RRID:BDSC_68299
D. melanogaster: *radish* mutant	Folkers et al., 1993	N/A
D. melanogaster: UAS-GCaMP6m	Bloomington Drosophila Stock Center; Chen et al., 2013	RRID:BDSC_42748
D. melanogaster: UAS-GtACR1	Mohammad et al., 2017	N/A
D. melanogaster: UAS-*Shi*^{ts1}	Kitamoto, 2001	N/A
**Software and Algorithms**		
Fiji	NIH; Schindelin et al., 2012	http://fiji.sc/
MATLAB R2017b	The Mathworks, Natick, MA	https://www.mathworks.com/products/matlab.html

(*Continued on next page*)

Continued

REAGENT or RESOURCE	SOURCE	IDENTIFIER
GraphPad Prism 7	GraphPad Software, La Jolla, CA	https://www.graphpad.com/scientific-software/prism/
Adobe Illustrator CC	Adobe Systems, San Jose, CA	https://www.adobe.com/uk/products/illustrator.html
ScanImage 3.8 software	Pologruto et al., 2003	https://vidriotechnologies.com/

RESOURCE AVAILABILITY

Lead Contact and Materials Availability

Further information and requests for resources and reagents should be directed to and will be fulfilled by the Lead Contact, Scott Waddell (scott.waddell@cncb.ox.ac.uk). This study did not generate new unique reagents.

DATA AND CODE AVAILABILITY

The datasets and customized MATLAB and Fiji scripts supporting the current study have not been deposited in a public repository because they are still in development, but are available from the Lead Contact on request and without restriction.

EXPERIMENTAL MODEL AND SUBJECT DETAILS

Fly strains

All *Drosophila melanogaster* strains were reared at 25°C and 40%–50% humidity on standard cornmeal-agar food in 12:12 h light:dark cycle. Flies from the wild-type (WT) Canton-S and mutant *radish* (Folkers et al., 1993) strains were used. Transgenes were expressed with previously described GAL4 lines: R58E02-GAL4 (Liu et al., 2012), MB110C-GAL4, MB504B-GAL4, MB056B-GAL4, MB441B-GAL4 and MB025B-GAL4 (Aso et al., 2014a, 2014b), R66C08-GAL4 (Owald et al., 2015), R39A05-GAL4 and R71D08 (Jenett et al., 2012); G0239-GAL4 (Pai et al., 2013). For behavioral experiments UAS-Shi^{ts1} (Kitamoto, 2001) and GtACR1 (Mohammad et al., 2017) were expressed under the control of the respective GAL4–line. For the imaging experiments UAS-GCaMP6m (Chen et al., 2013) was expressed with the respective GAL4. Behavioral experiments used 4 to 9-day old mixed-sex flies. Calcium imaging was performed on 3-8 day old mixed-sex flies.

METHOD DETAILS

Behavioral experiments

Male flies from the GAL4 lines were crossed to UAS-Shi^{ts} or GtACR1 females. Approximately 80-100 flies were placed in a 25 mL vial containing standard food and a 20 × 60 mm piece of filter paper for 14–22 h before behavioral experiments, except where noted. Odors used in all experiments were 4-methylcyclohexanol (MCH), 3-octanol (OCT) and isopentyl acetate (IAA) diluted in mineral oil to an odor dilution of ~1:10^3 (specifically, 8-12 μL OCT, 8-9 μL MCH or 16-18 μL IAA in 8 mL mineral oil). The concentrations of the odors vary slightly in order to achieve balanced naive avoidance between the two test odors (across genotypes and test days, etc.). Aversive learning does not differ when flies are trained with odors within this concentration range (Masek and Heisenberg, 2008; Felsenberg et al., 2018). All experiments were performed at 23°C, except where noted, and 55%–65% relative humidity.

For experiments involving neuronal blockade with Shi^{ts1}, the time courses of the temperature shifts are provided alongside each graph of memory performance. Flies were transferred to the restrictive 32°C 30 min before the targeted time, except where noted, to allow for acclimatization to the new temperature. Prior to optogenetic experiments all flies were housed on standard cornmeal food supplemented with 1 mM retinal for 3 days.

Aversive olfactory conditioning in the T-maze was conducted as previously described (Tully and Quinn, 1985; Perisse et al., 2016). Groups of flies were trained with either one cycle of aversive training, six consecutive cycles (massed training) or six cycles spaced by 15 min inter-trial intervals (spaced training) (Tully et al., 1994). After each cycle of spaced training flies were transferred from the training tube back into their starter vial until the start of the next cycle. Except where noted, during each cycle of training flies were exposed to a first odor for 1 min (the conditioned stimulus+, CS+) paired with twelve 90 V electric shocks at 5 s intervals. Following 45 s of clean air, a second odor (the conditioned stimulus-, CS−) was presented for 1 min without shock. Flies were kept in food vials at 23°C between training and test. Memory was subsequently assessed 24 h after training by testing flies for their odor preference between the CS- and the CS+ or the CS+ or CS- versus novel odor in a T-maze (2 min in darkness).

The testing odors were always MCH and OCT. To isolate the individual CS+ and CS- memories the novel odor IAA was introduced during training where it replaced either the CS- or CS+ odor. Briefly, for testing the CS+ memory; in half of the reciprocal training experiments MCH was used as CS+ and OCT was the CS+ in the others, IAA was always the CS-. For testing the CS- memory,

in half of the reciprocal training MCH was used as CS- and OCT was CS- in the others, IAA was always the CS+. Performance Index was calculated as the number of flies in the CS+ arm minus the number in the CS- arm, divided by the total number of flies (Tully and Quinn, 1985). When the performance was tested against a novel odor the Performance Index was calculated as the number of flies in the CS+ or CS- arm minus the number in the Novel arm, divided by the total number of flies. A single sample, or n, represents the average performance score from two reciprocally trained groups.

To test CS+ memory and CS- memory against Air, the flies were trained with the different paradigms using MCH and OCT, in a reciprocal manner. Flies then chose between CS+ versus Air (i.e, air bubbled through mineral oil). Alternatively flies chose CS- versus Air. Performance Index was calculated as the number of flies in the odor (MCH or OCT) arm minus the number in the Air arm, divided by the total number of flies.

For each trial of spaced relief training, flies were exposed to air for 1 min paired with twelve 90 V electric shocks at 5 s intervals. Following an ISI of 45, 90 or 135 s with clean air, the odor (A) was presented for 1 min without shock. Training cycles were separated with a 15 min ITI. MCH and OCT were used as odor A in a reciprocal manner. For testing, flies chose between odor A and a Novel odor. When odor A was MCH the novel odor was OCT, and vice-versa. Flies were either tested immediately after the last training cycle or 24 h later. Flies were kept in food vials at 23°C between training and test.

To test olfactory acuity, untrained flies were given 2 min to choose between a diluted Odor (specifically, 9 μL OCT, 9 μL MCH or 17 μL IAA in 8 mL mineral oil) as used in conditioning and Air bubbled through mineral oil in the T-Maze. An Avoidance Index was calculated as the number of flies in the Odor arm minus the number in the Air arm. To test shock acuity, untrained flies were given 1 min to choose between a tube containing an electrified grid (12 90V shocks) and a tube containing a non-electrified grid. Avoidance Index was calculated as the number of flies in the electrified arm minus the number in the non-electrified arm. No statistical differences were observed between the relevant genotypes (Table S2). An individual *n*, represents a single experiment.

CXM feeding

WT flies were fed with cycloheximide (CXM) for 12-16 h prior to training as reported before (Tully et al., 1994; Yin et al., 1994). In brief, filter paper strips were soaked with 250 μL 5% glucose solution laced with 35 mM CXM. For control flies the filter paper strips were soaked with 250 μL 5% glucose. Flies were then transferred to the training apparatus and subjected to spaced training. They were then transferred to test tubes containing filter paper strips soaked with 5% glucose during the 24 h retention interval before testing.

Two-Photon Calcium Imaging

3-8 day old flies were imaged 23-25 h after aversive conditioning. Flies were trained as described above. Imaging experiments were performed essentially as described previously (Owald et al., 2015; Perisse et al., 2016; Felsenberg et al., 2018). In brief, flies were immobilized on ice and mounted in a custom-made chamber allowing free movement of the antennae and legs. The head capsule was opened under room temperature carbogenated (95% O_2, 5% CO_2) buffer solution (103 mM NaCl, 3 mM KCl, 5mM N-Tris, 10 mM trehalose, 10 mM glucose, 7mM sucrose, 26 mM $NaHCO_3$, 1mM NaH_2PO_4, 1.5 mM $CaCl_2$, 4mM $MgCl_2$, osmolarity 275 mOsm, pH 7.3) and the fly, in the recording chamber, was placed under the Two-Photon microscope (Scientifica).

For imaging MBONs, a constant air stream, carrying vapor from mineral oil solvent (air) was applied. GCaMP responses to the CS+, the CS- and a third odor were measured in the relevant MBONs. Flies were sequentially exposed to the CS+, CS- and a third odor, isopentyl acetate (IAA; 1:10^3 odor concentration) for 5 s. Each odor presentation was followed by 30 s of air. To image the dendritic field and axonal segments of MBON-γ3,γ3β′1, the axonal segments of the MBON-β′2mp and MBON-γ5β′2a processes, the dendritic field of MBON-α2sc and MBON-α3, one hemisphere of the brain was randomly selected. To measure responses in the MBON-β′2mp and MBON-γ5β′2a dendrites, signals were simultaneously acquired from both hemispheres and averaged responses were analyzed.

For imaging DANs, flies were exposed to a protocol composed of a pre-phase, a training-phase and post-phase. In the pre and post-phase, flies were presented with the CS+ and CS- for 5 s. Each odor presentation was followed by 30 s of air. The training-phases follow the behavioral training protocols for spaced training and reversed spaced training. The first, third and sixth training trials were imaged.

Fluorescence was excited using ~140 fs pulses, 80 MHz repetition rate, centered on 910 nm generated by a Ti-Sapphire laser (Chameleon Ultra II, Coherent). Images of 256 × 256 pixels were acquired at 5.92 Hz, controlled by ScanImage 3.8 software (Pologruto et al., 2003). Odors were delivered using a custom-designed system (Shang et al., 2007).

For analysis, two-photon fluorescence images were manually segmented using Fiji (Schindelin et al., 2012). Movement of the animals was small enough such that images did not require registration. For subsequent quantitative analyses, custom Fiji and MATLAB scripts were used. The baseline fluorescence, F_0, was defined for each stimulus response as the mean fluorescence F from 2 s before and up to the point of odor presentation. F/F_0 accordingly describes the fluorescence relative to this baseline.

For the MBON imaging, the area under the curve (AUC) was measured as the integral of F/F_0 during the 5 s odor stimulation. To account for variance between individual flies, the responses of the CS+ and CS− were normalized to the response to IAA. Each AUC was divided by the IAA AUC from the respective trial and individual fly.

For the DAN imaging, in the pre- and post-phase the area under the curve (AUC) was measured as the integral of F/F_0 during the 5 s odor presentation. In the training phase, the mean fluorescence response during the 60 s odor presentation was calculated for each odor. The mean fluorescence was chosen due to the long recording during training, which had more baseline shifts than the short

recordings (for the pre- and post-phase). For the shock analysis, the DAN responses were averaged for the 12 shocks in each training cycle and the mean fluorescence response for the 3 s after the onset of the shock was calculated.

Exclusion criteria for the analyses applied in this study were: flies that did not respond to either of the two training odors, or if they did not respond to the IAA used for normalization (in the case of MBON imaging). Since the number of excluded flies was not always the same for both CS+ odors and for the different paradigms and different regions, this can lead to a different final *n*. Each *n* corresponds to a recording from a single fly. For each MBON imaged, the total number of flies came from 3 different training sessions.

QUANTIFICATION AND STATISTICAL ANALYSIS

Statistical analyses were performed in GraphPad Prism. For the behavioral data, unpaired t-tests were used to compare two relevant groups (e.g., CS+ versus CS- memory and CS+ versus novel odor; *rad* and CXM experiments). Given the nature of the CS- versus novel odor memory any statistical comparison with CS+ versus CS- memory or CS+ versus novel odor was not appropriate. To analyze if an avoidance for the CS+ or an approach for the CS- was observed one-sample t-test was used to test for a difference between a theoretical mean of 0 (i.e., significant difference from zero means that flies either avoid or approach the odor, respectively). One-way ANOVA followed by Dunnett's multiple comparisons test (for planned comparisons to a specific group) or Tukey's multiple comparisons test (for comparison between different genotypes) were used as post hoc tests to compare data between groups. No statistical methods were used to predetermine sample size.

For the imaging experiments normalized responses were compared by a paired t-test for normally distributed data, otherwise a Wilcoxon matched-pairs signed rank test was used for non-Gaussian distributed data. Normality was tested using the Shapiro-Wilk normality test. Repeated-measures ANOVA followed by Bonferroni's multiple comparisons test was used to compare pre versus post odor-evoked responses in DANs. For imaging data, a method for outlier identification was run for each dataset (ROUT method), which is based on the False Discovery Rate (FDR). The FDR was set to the highest Q value possible (10%). In the datasets in which potential outliers were identified, statistical analyses were performed by removing the CS+ and CS- responses for those flies. The analyses with or without the outliers were not different, so we decided to maintain and present the complete datasets, which may contain potential outliers.

All statistical tests used, the *n* numbers and the *p* values are shown in Table S1.

Neuron, Volume *106*

Supplemental Information

Spaced Training Forms Complementary Long-Term Memories of Opposite Valence in *Drosophila*

Pedro F. Jacob and Scott Waddell

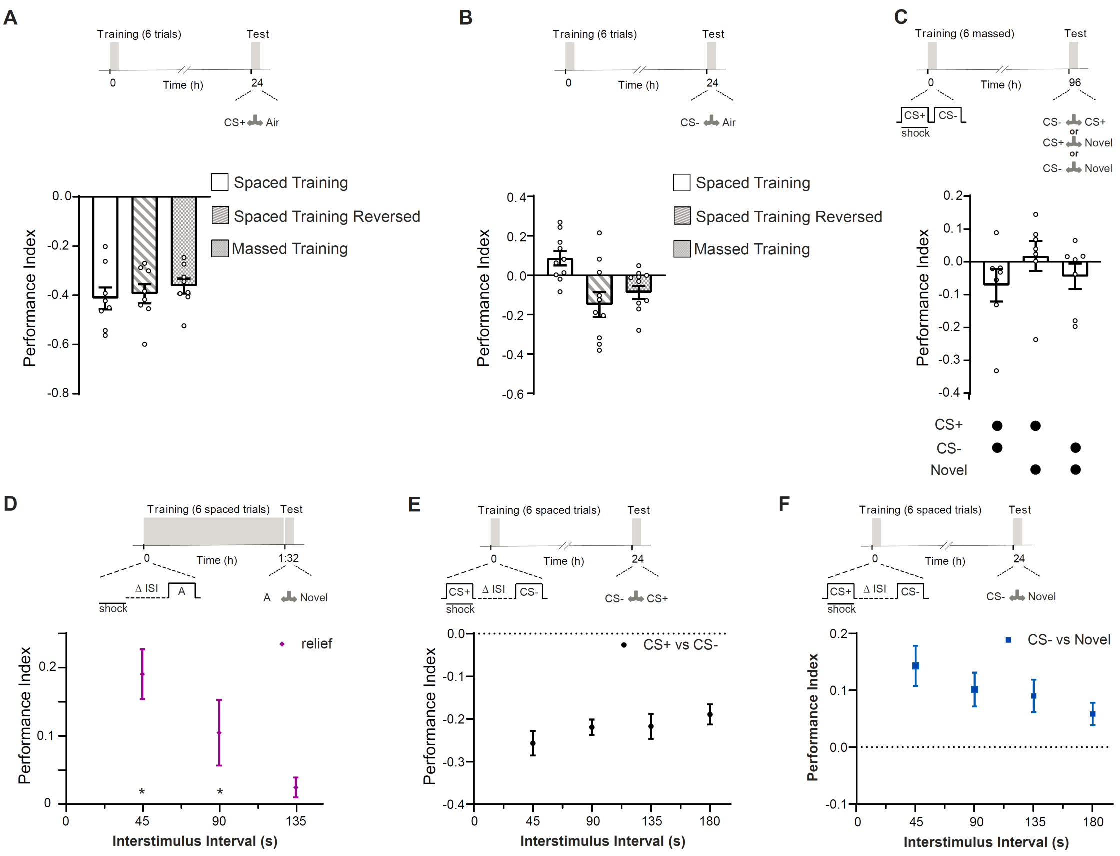

Figure S1. Related to Figure 1.

Spaced training forms CS+ aversive memory and CS- approach memory.

(**A**) Spaced, reversed spaced and massed training generated a similar 24 h CS+ aversive memory when flies were tested CS+ *vs.* Air. (**B**) A 24 h CS- approach memory (CS- *vs.* Air), was only measurable following spaced training spaced. After reversed spaced and massed training flies avoided the CS- odor. (**C**) Massed training did not generate measurable 96 h memory. (**D**) Spaced relief training (6 trials, shock then odor A training, with 15 min ITI) generated approach memory to odor A, when measured immediately after training. Relief memory to odor A was not formed when ISI was increased from 45 to 135 s. Varying ISI between CS+ and CS- in spaced training produced similar 24 h LTM (**E**) and CS- approach memory (**F**). Asterisks denote significant difference. Data mean ± SEM. Individual data points displayed as dots. See Table S1 for statistics.

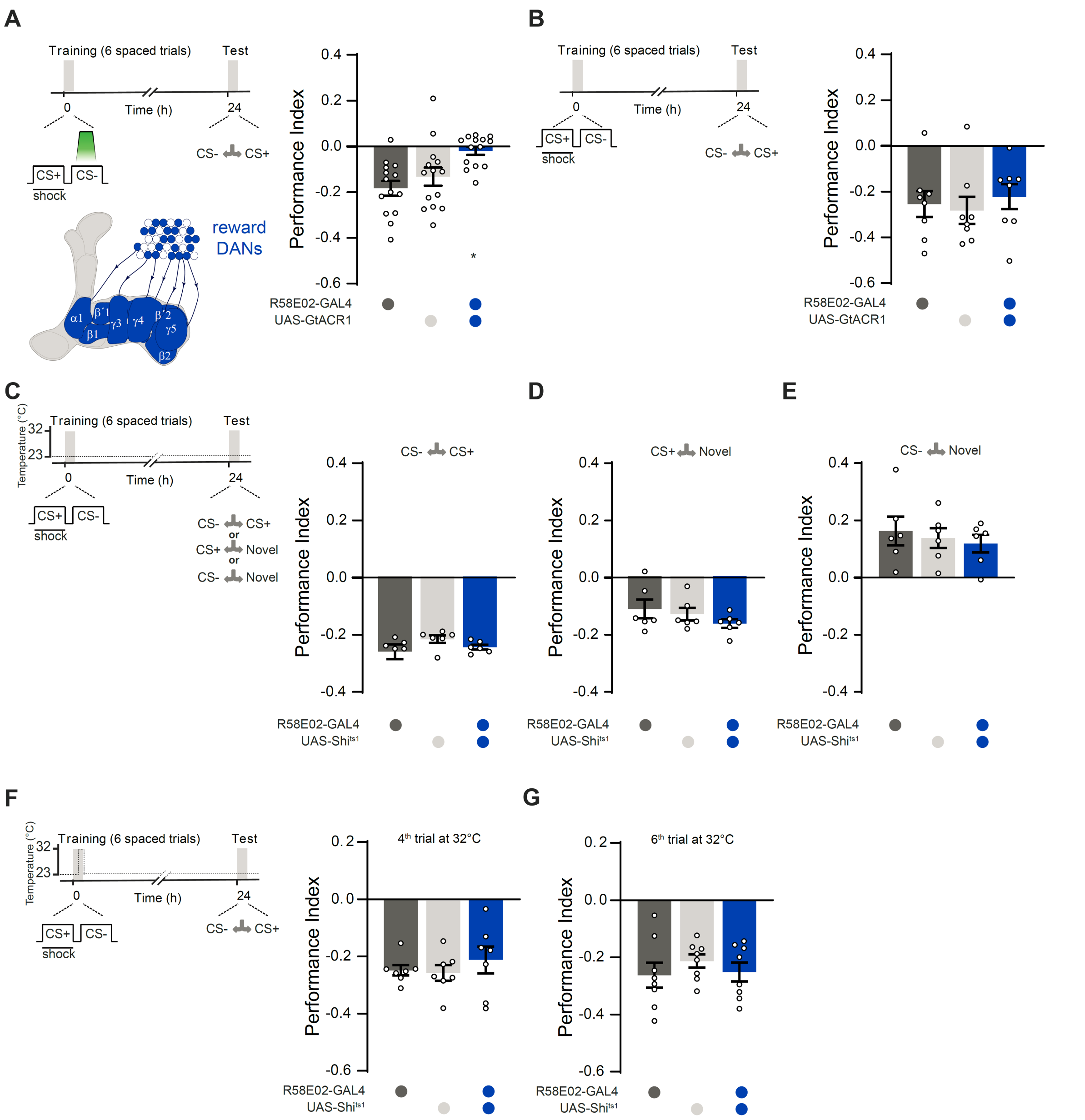

Figure S2. Related to Figure 2.

CS- approach memory requires PAM DANs during CS- presentation.

(**A**) Left: protocol with green light exposure during CS- presentation and PAM/R58E02-GAL4 DAN schematic. Right: Blocking PAM DANs with R58E02-GAL4; UAS-GtACR1 during CS- impaired 24 h memory. (**B**) Left: protocol without green light. Right: UAS-GtACR1 expression in PAM DANs (R58E02-GAL4) does not disrupt 24 h memory after spaced training. (**C**) Left: protocol. Right: Expressing UAS-*Shi*^{ts1} in R58E02-GAL4 DANs does not disrupt 24 h memory after spaced training at permissive 23˚C. (**D**) CS+ aversive memory is unaffected. (**E**) CS- approach memory is unaffected. (**F**) Left: protocol with temperature shifting, dashed line. Right: Blocking DANs with R58E02-GAL4; UAS-*Shi*^{ts1}, only during the 4th trial or (**G**) only the 6th trial did not alter 24 h memory. Asterisks denote significant differences. Data mean ± SEM. Individual data points displayed as dots. See Table S1 for statistics.

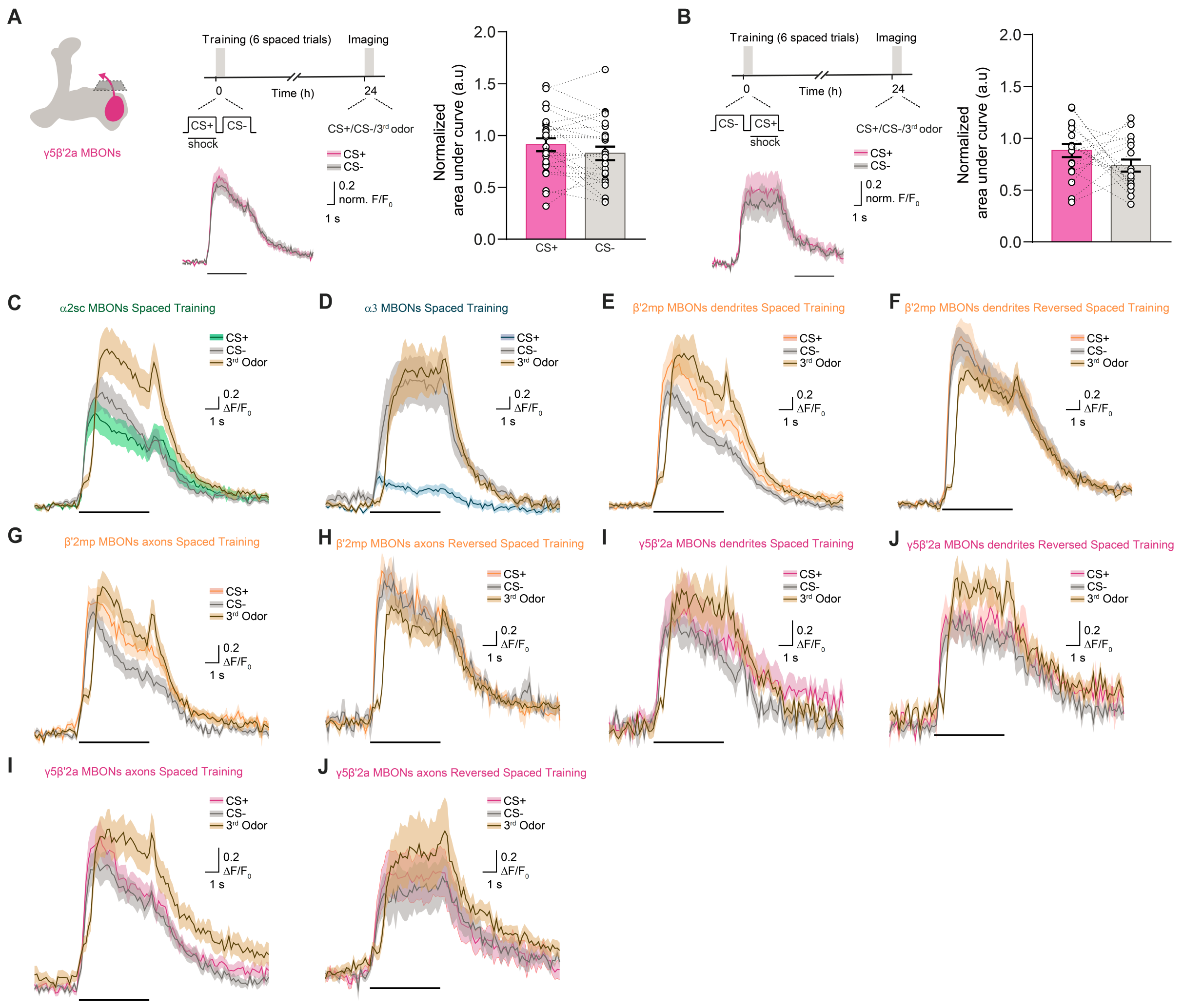

Figure S3. Related to Figure 3.

Parallel aversive and safety memories can be recorded as depression of odor-specific responses in corresponding MBONs.

Odor responses in MBON-γ5β′2a are unchanged following spaced training. Imaging plane in presynaptic terminals of MBON-γ5β′2a and training and imaging protocols. (**A**) Spaced training and (**B**) reversed spaced training do not change odor-evoked responses in MBON-γ5β′2a. (**C-J**) Non-normalized imaging traces for the MBON recordings reported in Figure 3 and S3A and B, in the order depicted in each figure. Odor-evoked activity traces show mean (solid line) with SEM (shadow). Black line underneath, 5 s odor. Asterisks denote significant difference between averaged CS+ and CS- responses. See Table S1 for statistics.

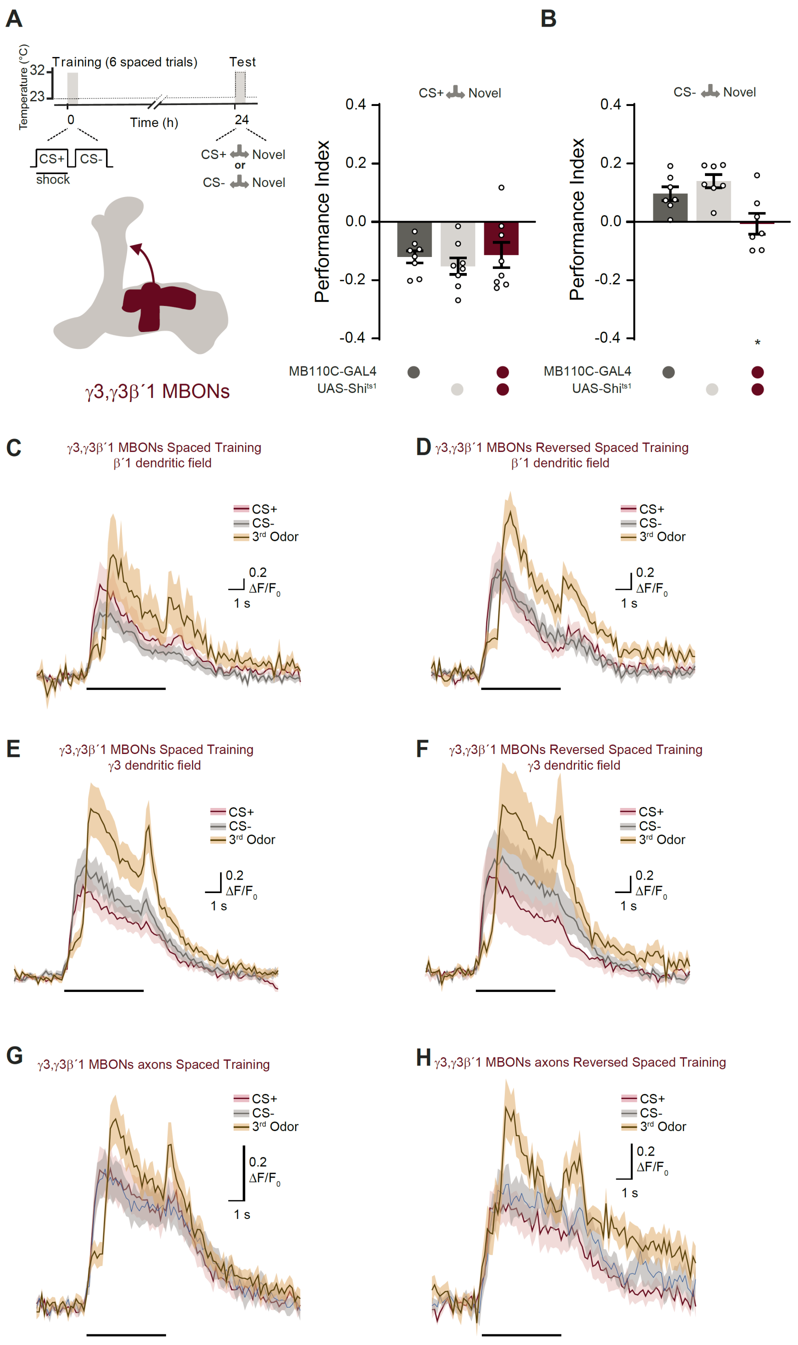

Figure S4. Related to Figure 4.

Output from γ3, γ3β′1 MBONs is required for expression of CS- approach memory.

(**A**) Left: protocol with temperature shifting (dashed line) and illustration of γ3, γ3β′1 MBONs. Right: blocking γ3, γ3β′1 MBONs during testing with MB110C-GAL4/UAS-*Shi*^{ts1} did not impair CS+ aversive memory (CS+ *vs*. Novel). (**B**) Blocking γ3, γ3β′1 MBONs during testing impaired CS- approach memory. (**C-H**) Non-normalized imaging traces for the recordings of γ3, γ3β′1 MBON responses reported in Figure 4, in the order they are depicted in the figure. Odor-evoked activity traces show mean (solid line) with SEM (shadow). Black line underneath, 5 s odor. Asterisks denote significant difference. Data mean ± SEM. Individual data points displayed as dots. See Table S1 for statistics.

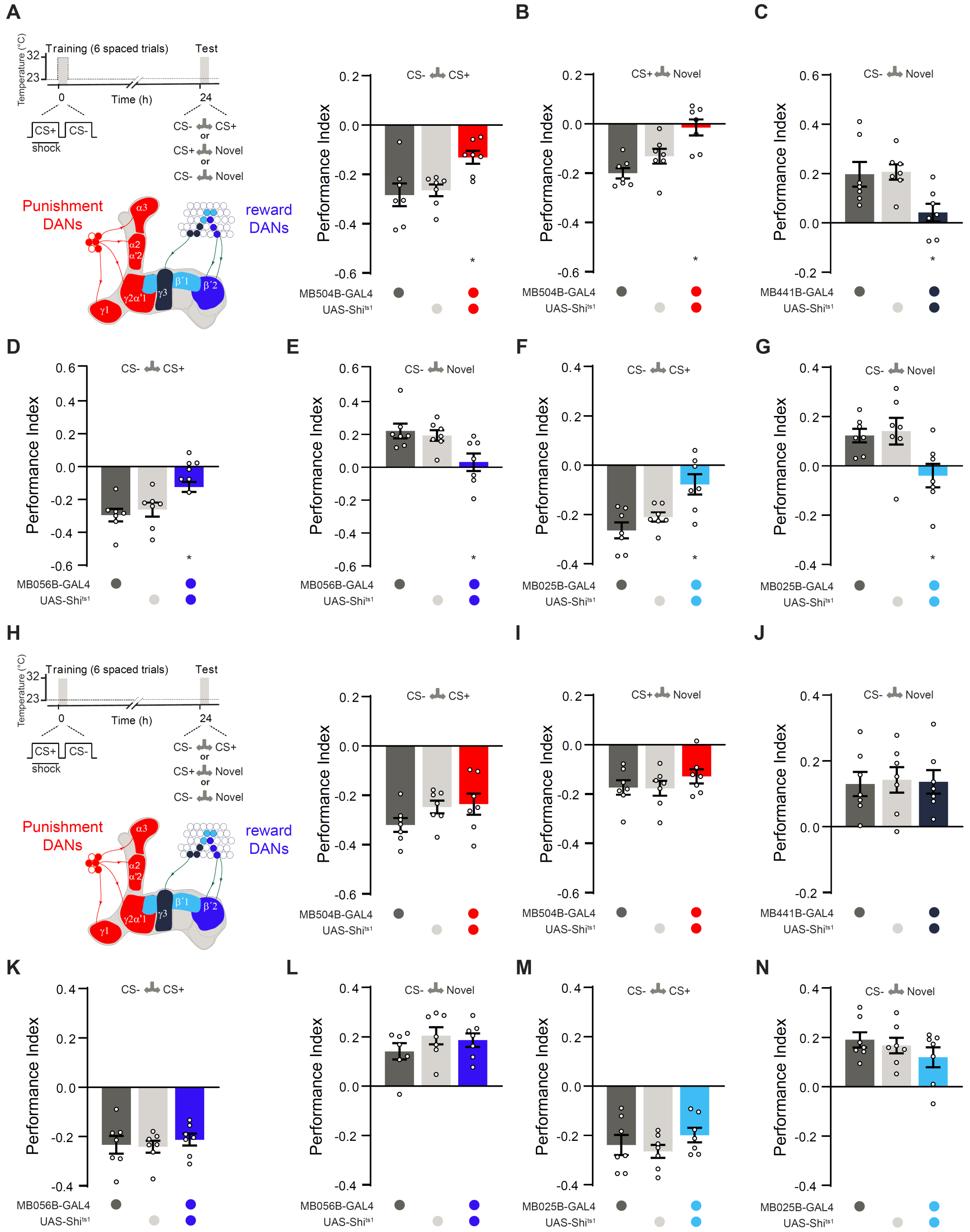

Figure S5. Related to Figure 5.

Blocking specific dopaminergic neurons during spaced training localises discrete sites of aversive and safety memory.

GAL4 driver and temperature control experiments for data presented in Figure 5. (**A**) Left: protocol with temperature shifting (dashed line) and color-coded illustration of DANs labelled with each GAL4. Right: blocking PPL1-DANs during spaced training with MB504B-GAL4; UAS-*Shi*^{ts1} impaired 24 h memory and (**B**) CS+ aversive memory (CS+ *vs*. Novel). (**C**) CS- approach memory was impaired with PAM-γ3 block. (**D**) Blocking PAM-β′2mp impaired 24 h memory and (**E**) CS- approach memory. (**F**) Blocking PAM-β′1 impaired 24 h memory and

(**G**) CS- approach memory. (**H**) Left: protocol (dashed line) and color-coded illustration of DANs labelled with each GAL4. Right: Expressing UAS-*Shi*^{ts1} in PPL1-DANs (MB504B-GAL4) did not disrupt 24 h memory after spaced training at permissive 23˚C. (**I**) CS+ aversive memory was also unaffected. (**J**) CS- approach memory was unaffected for MB441B-GAL4; UAS-*Shi*^{ts1}(PAM-γ3) flies at permissive 23˚C. (**K**) At permissive 23˚C, 24 h memory of MB056B-GAL4; UAS-*Shi*^{ts1} (PAM-β′2mp) flies was unaffected, as was (**L**) MB056B-GAL4; UAS-*Shi*^{ts1} CS- approach memory, (**M**) MB025B-GAL4; UAS-*Shi*^{ts1} (PAM-β′1) 24 h memory, and (**N**) MB025B-GAL4; UAS-*Shi*^{ts1} CS- approach memory. Asterisks denote significant differences. Data mean ± SEM. Individual data points displayed as dots. See Table S1 for statistics.

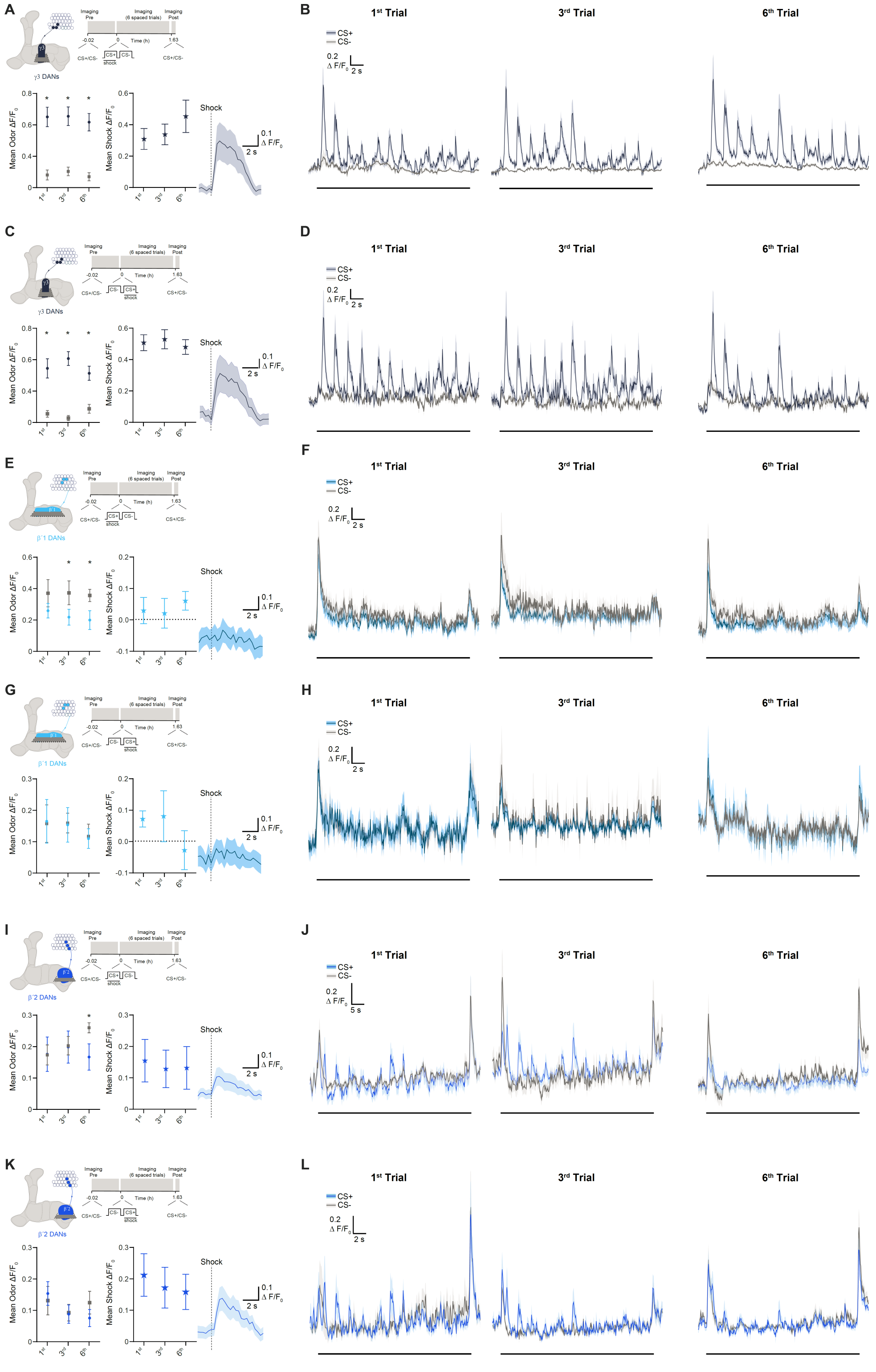

Figure S6. Related to Figure 6.

Spaced training enhanced CS+ responses in aversive γ3 DANs and CS- responses in rewarding β′1 and β′2mp DANs.

(**A and C**) Top: Imaging plane in the presynaptic field of γ3 DANs and training and imaging protocol. (**A**) Bottom left panel: From the 1st training trial the CS+ showed an increased

mean response relative to the CS-. This was also observed during reversed spaced training (**C**). (**A and C**) Bottom right panels: γ3 DANs showed a strong calcium response to electric shock. (**B and D**) Calcium responses during the 1st, 3rd and 6th training trials. Odor-evoked activity traces show mean (solid line) with SEM (shadow). Black line underneath, 60 s odor. (**E**) Top: Imaging plane in the presynaptic field of β′1 DANs and training and imaging protocol. Bottom left: From the 3rd training trial CS- responses were increased relative to CS+ responses. Bottom right: β′1 DANs did not respond to shock with an increase in calcium. (**F**) Calcium responses during the 1st, 3rd and 6th trials of spaced training. Horizontal line denotes odor stimulus. (**G**) Top: Imaging plane in the presynaptic field of β′1 DANs and training and imaging protocol. Bottom left: No differences between the CS+ and CS- responses were observed during reversed spaced training. Bottom right: β′1 DANs did not respond to shock with an increase in calcium. (**H**) Calcium responses during the 1st, 3rd and 6th trials of reversed spaced training. Horizontal line denotes odor stimulus. (**I**) Top: Imaging plane in the presynaptic field of β′2mp DANs and training and imaging protocol. Bottom left: At the 6th training trial, the CS- evoked an increased mean response (over the 60 s of odor presentation) in comparison to the CS+, but not for the other trials analyzed. Bottom right: Aligning the activity of β′2mp DANs to time of each of the 12 shocks (dashed line) reveals a shock-evoked response. Calcium responses were calculated by averaging the fluorescence over the 3 s following each shock. (**J**) β′2mp DAN calcium responses during the 1st, 3rd and 6th trials of spaced training. Horizontal line denotes odor stimulus. (**K**) Top: Imaging plane in presynaptic field of β′2mp DANs and training and imaging protocol. Bottom left: No changes were observed between CS+ and CS- responses during reversed spaced training. Bottom right: β′2mp DANs responded to shock. (**L**) β′2mp DAN calcium responses during the 1st, 3rd and 6th trial of reversed spaced training. Horizontal line denotes odor stimulus. CS+ data corresponds to average data in which 50% of trials used MCH as CS+ and 50% were OCT CS+. Same applies for CS- data. Asterisks denote significant difference between averaged CS+ and CS- responses. See Table S1 for statistics.

Table S1. Statistical details. Related to Figures 1-6 and S1-S6.

Figure	Experiment	n	Normally distributed	Statistical test	p value
Figure 1A	CS+ memory (CS+ vs Novel) vs. LTM (CS+ vs CS-); spaced training	8-13	yes	unpaired t-test t(19)= 2.890	p=0.0094
	CS- vs Novel; spaced training	10	yes	one sample t-test t(9)=3.312	p=0.0091
Figure 1B	CS+ memory (CS+ vs Novel) vs. LTM (CS+ vs CS-); reversed spaced training	8-11	yes	unpaired t-test t(17)=0.6628	p=0.5163
	Performance of CS- vs Novel different from zero; reversed spaced training	8	yes	one sample t-test t(7)=1.546	p=0.1660
Figure 1C	CS+ memory (CS+ vs Novel) vs. LTM (CS+ vs CS-); massed training	10-15	yes	unpaired t-test t(23)=0.7649	p=0.4521
	Performance of CS- vs Novel different from zero; massed training	10	yes	one sample t-test t(9)=1.151	p=0.2792
Figure 1D	CS+ memory (CS+ vs Novel) vs. LTM (CS+ vs CS-); fasting LTM protocol	10-11	yes	unpaired t-test t(19)=1.802	p=0.09
	Performance of CS- vs Novel different from zero; fasting LTM protocol	10	yes	one sample t-test t(9)=0.4190	p=0.6850
Figure 1E	Time-line of CS+ vs. CS- memory (all time points)	8-10	yes	F[5,44]=20.61	p<0.0001
	Immediate vs. 24 h			Dunnett's multiple comparisons test	p=0.0011
	30 min vs. 24 h				p=0.0122
	3 h vs. 24 h				p=0.0043
	14 h vs. 24 h				p=0.9998
	96 h vs. 24 h				p=0.0028
Figure 1F	Time-line of CS- vs. Novel (all time points)	8-10	yes	F[5,44]=4.33	p=0.0028
	Immediate vs. 24h			Dunnett's multiple comparisons test	p=0.0026
	30 min vs. 24 h				p=0.047
	3 h vs. 24 h				p=0.3717
	14 h vs. 24 h				p=0.9997
	96 h vs. 24 h				p=0.8464
	Performance of CS- vs. Novel different from zero @ Immediate time point	8	yes	one sample t-test t(7)=0.6530	p=0.5346
	Performance of CS- vs. Novel different from zero @ 30 min time point	8		one sample t-test(7)=0.9635	p=0.3674
	Performance of CS- vs. Novel different from zero @ 3 h time point	8		one sample t-test(7)=1.931	p=0.0948
	Performance of CS- vs. Novel different from zero @ 14 h time point	8		one sample t-test(7)=5.557	p<0.0001
	Performance of CS- vs. Novel different from zero @ 24 h time point	8		one sample t-test(7)= 9.305	p=0.0009
	Performance of CS- vs. Novel different from zero @ 96 h time point	10		one sample t-test(9)= 2.885	p=0.0180
	Time-line of CS+ vs. Novel (all time points)	8-10	yes	F[5,44]=20.61	p<0.0001
	Immediate vs. 24h			Dunnett's multiple comparisons test	p=0.0011
	30 min vs. 24 h				p=0.0122
	3 h vs. 24 h				p=0.0043
	14 h vs. 24 h				p=0.9921
	96 h vs. 24 h				p=0.0028
	Performance of CS- vs. Novel different from zero @ Immediate timepoint	10	yes	one sample t-test t(9)=0.1127	p=0.9219
Figure 1G	*rad* vs. WT; spaced training LTM (CS+ vs CS-)	7	yes	unpaired t-test t(12)=2.225	p=0.0416
	rad vs. WT; spaced training CS+ memory (CS+ vs Novel)	8	yes	unpaired t-test t(14)=2.969	p=0.0102
	rad vs. WT; spaced training CS- memory (CS- vs Novel)	7	yes	unpaired t-test t(12)=0.2593	p=0.7998
Figure 1G	CXM vs. WT; spaced training LTM (CS+ vs CS-)	7-8	yes	unpaired test t(13)=3.943	p=0.0017
	CXM vs. WT; spaced training CS+ memory (CS+ vs Novel)	9-12	yes	unpaired t-test t(19)=2.703	p=0.0141
	CXM vs. WT; spaced training CS- memory (CS- vs Novel)	11-13	yes	unpaired t-test t(22)=1.720	p=0.0995
Figure 2A	LTM performance (CS+ vs CS-); PAM block	14-15	yes	F[2,41]=24.93	p<0.0001
	R58E02-GAL4; UAS-Shi^{ts1} vs.+; UAS-Shi^{ts1}			Tukey's multiple comparisons test	p<0.0001
	R58E02-GAL4; UAS-Shi^{ts1} vs. R58E02-GAL4; +				p<0.0001
	R58E02-GAL4; + vs.+; UAS-Shi^{ts1}				p=0.4404
Figure 2B	CS+ memory (CS+ vs Novel); PAM block	6	yes	F[2,15]=0.59	p=0.5668
Figure 2C	CS- memory (CS- vs Novel); PAM block	7-8	yes	F[2,19]=26.32	p<0.0001
	R58E02-GAL4; UAS-Shi^{ts1} vs.+; UAS-Shi^{ts1}			Tukey's multiple comparisons test	p<0.0001
	R58E02-GAL4; UAS-Shi^{ts1} vs. R58E02-GAL4; +				p=0.0002
	R58E02-GAL4; + vs.+; UAS-Shi^{ts1}				p=0.3370
Figure 2D	LTM performance (CS+ vs CS-); PAM block during the 1st and 2nd trial	8	yes	F[2,21]=11.40	p=0.0004
	R58E02-GAL4; UAS-Shi^{ts1} vs.+; UAS-Shi^{ts1}			Tukey's multiple comparisons test	p=0.0013
	R58E02-GAL4; UAS-Shi^{ts1} vs. R58E02-GAL4; +				p=0.0014
	R58E02-GAL4; + vs.+; UAS-Shi^{ts1}				p=0.9992
Figure 2E	LTM performance (CS+ vs CS-); PAM block during the 3rd and 4th trial	8	yes	F[2,21]=9.209	p=0.0004
	R58E02-GAL4; UAS-Shi^{ts1} vs.+; UAS-Shi^{ts1}			Tukey's multiple comparisons test	p=0.0272
	R58E02-GAL4; UAS-Shi^{ts1} vs. R58E02-GAL4; +				p=0.0011
	R58E02-GAL4; + vs.+; UAS-Shi^{ts1}				p=0.3565
Figure 2F	LTM performance (CS+ vs CS-); PAM block during the 5th and 6th trial	8	yes	F[2,21]=7.741	p=0.003
	R58E02-GAL4; UAS-Shi^{ts1} vs.+; UAS-Shi^{ts1}			Tukey's multiple comparisons test	p=0.0083
	R58E02-GAL4; UAS-Shi^{ts1} vs. R58E02-GAL4; +				p=0.0062
	R58E02-GAL4; + vs.+; UAS-Shi^{ts1}				p=0.9916

Figure 3A	R71D08-GAL4 after spaced training	28	yes	paired t-test t(27)=4.277	p=0.0002
Figure 3B	G0239-GAL4 after spaced training	20	no	Wilcoxon matched-pairs signed rank W(19)=169	p<0.0001
Figure 3C	R39A05-GAL4 after spaced training, dendrites	28	yes	paired t-test t(27)=3.374	p=0.023
Figure 3D	R39A05-GAL4 after spaced training reverse order, dendrites	24	yes	paired t-test t(23)=0.19	p=0.851
Figure 3E	R39A05-GAL4 after spaced training, presynaptic terminals	34	yes	paired t-test t(33)=2.639	p=0.013
Figure 3F	R39A05-GAL4 after spaced training reverse order, presynaptic terminals	24	yes	paired t-test t(23)=0.1181	P=0.118
Figure 3G	R66C08-GAL4 after spaced training, dendrites	20	yes	paired t-test t(19)=0.1799	p=0.8591
Figure 3H	R66C08-GAL4 after spaced training reverse order, dendrites	20	yes	paired t-test t(19)=0.03199	p=0.9748
Figure 4A	MB110C-GAL4 after spaced training, β′1 dendrites	34	yes	paired t-test t(33)=3.793	p=0.0006
Figure 4B	MB110C-GAL4 after spaced training reverse order, β′1 dendrites	30	no	Wilcoxon matched-pairs signed rank W(29)=31	p=0.749
Figure 4C	MB110C-GAL4 after spaced training, γ3 dendrites	36	no	Wilcoxon matched-pairs signed rank W(35)=308	p=0.011
Figure 4D	MB110C-GAL4 after spaced training reverse order, γ3 dendrites	30	yes	paired t-test t(29)=2.140	p=0.04
Figure 4E	MB110C-GAL4 after spaced training, presynaptic terminals	34	yes	paired t-test t(33)=0.1275	p=0.899
Figure 4F	MB110C-GAL4 after spaced training reverse order, presynaptic terminals	22	yes	paired t-test t(21)=1.535	p=0.14
Figure 5A	LTM performance (CS+ vs CS-); DAN block	11-13	yes	F[4,53]=5.093	p=0.0015
	MB504B-GAL4; UAS-Shi^{ts1} vs.+; UAS-Shi^{ts1}			Dunnett's multiple comparisons test	p=0.0024
	MB441B-GAL4; UAS-Shi^{ts1} vs.+; UAS-Shi^{ts1}				p=0.8683
	MB056B-GAL4; UAS-Shi^{ts1} vs.+; UAS-Shi^{ts1}				p=0.0055
	MB025B-GAL4; UAS-Shi^{ts1} vs.+; UAS-Shi^{ts1}				p=0.0429
Figure 5B	CS+ memory (CS+ vs Novel); DAN block	8-9	yes	F[4,37]=3.371	p=0.0189
	MB504B-GAL4; UAS-Shi^{ts1} vs.+; UAS-Shi^{ts1}			Dunnett's multiple comparisons test	p=0.0362
	MB441B-GAL4; UAS-Shi^{ts1} vs.+; UAS-Shi^{ts1}				p=0.9663
	MB056B-GAL4; UAS-Shi^{ts1} vs.+; UAS-Shi^{ts1}				p=0.9992
	MB025B-GAL4; UAS-Shi^{ts1} vs.+; UAS-Shi^{ts1}				p=0.9997
Figure 5C	CS- memory (CS- vs Novel); DAN block	8	yes	F[4,335]=11.76	p<0.0001
	MB504B-GAL4; UAS-Shi^{ts1} vs.+; UAS-Shi^{ts1}			Dunnett's multiple comparisons test	p=0.8466
	MB441B-GAL4; UAS-Shi^{ts1} vs.+; UAS-Shi^{ts1}				p=0.0002
	MB056B-GAL4; UAS-Shi^{ts1} vs.+; UAS-Shi^{ts1}				p=0.0007
	MB025B-GAL4; UAS-Shi^{ts1} vs.+; UAS-Shi^{ts1}				p=0.0443
Figure 6B	MB441B-GAL4 after spaced training	20	yes	Repeated measures ANOVA, F[19,57]=5.089	p=0.0102
	pretraining CS+ vs. pretraining CS-			Bonferroni's multiple comparisons test	p>0.9999
	pretraining CS+ vs. posttraining CS+				p=0.0388
	pretraining CS+ vs. posttraining CS-				p=0.0001
	pretraining CS- vs. posttraining CS-				p>0.9999
Figure 6D	MB441B-GAL4 after reversed spaced training	20	yes	Repeated measures ANOVA, F[19,57]=6.417	p=0.0066
	pretraining CS+ vs. pretraining CS-			Bonferroni's multiple comparisons test	p>0.9999
	pretraining CS+ vs. posttraining CS+				p<0.0001
	pretraining CS+ vs. posttraining CS-				P=0.0406
	pretraining CS- vs. posttraining CS-				p>0.9999
Figure 6F	MB025B-GAL4 after spaced training	20	yes	Repeated measures ANOVA, F[19,57]= 5.244	p=0.0066
	pretraining CS+ vs. pretraining CS-			Bonferroni's multiple comparisons test	p=0.6615
	pretraining CS+ vs. posttraining CS+				p=0.0254
	pretraining CS+ vs. posttraining CS-				p=0.0019
	pretraining CS- vs. posttraining CS-				p=0.2918
Figure 6H	MB025B-GAL4 after reversed spaced training	20	yes	Repeated measures ANOVA, F[19,57]= 6.063	p=0.0045
	pretraining CS+ vs. pretraining CS-			Bonferroni's multiple comparisons test	p=0.1846
	pretraining CS+ vs. posttraining CS+				p=0.0048
	pretraining CS+ vs. posttraining CS-				p>0.9999
	pretraining CS- vs. posttraining CS-				p=0.045
Figure 6J	MB056B-GAL4 after spaced training	20	yes	Repeated measures ANOVA, F[19,57]=8.042	p=0.0012
	pretraining CS+ vs. pretraining CS-			Bonferroni's multiple comparisons test	p=0.5877
	pretraining CS+ vs. posttraining CS+				p>0.9999
	pretraining CS+ vs. posttraining CS-				p=0.0276
	pretraining CS- vs. posttraining CS-				p=0.0041
Figure 6L	MB056B-GAL4 after reversed spaced training	20	yes	Repeated measures ANOVA, F[19,57]=1.306	p=0.283

Figure S1A	CS+ vs. Air; different training regimens	8	yes	F[2,21]=0.4275	p=0.65
Figure S1B	CS- vs. Air; different training regimens	10	yes	F[2,27]=7.609	p=0.0024
	spaced vs. reversed spaced			Tukey's multiple comparisons test	p=0.0021
	spaced vs. massed				p=0.0313
	reversed spaced vs. massed				p=0.5231
	Performance of CS- vs. Air different from zero; spaced training			one sample t-test t(9)=2.375	p=0.0416
	Performance of CS- vs. Air different from zero; reversed spaced training			one sample t-test t(9)=2.511	p=0.0332
	Performance of CS- vs. Air different from zero; massed training			one sample t-test t(9)=2.595	p=0.0290
Figure S1C	Performance of CS+ vs. CS- (LTM) different from zero; massed training 96 h	7	yes	one sample t-test t(6)=1.434	p=0.2016
	Performance of CS+ vs. Novel different from zero; massed training 96 h			one sample t-test t(6)=0.3854	p=0.7133
	Performance of CS- vs. Novel different from zero; massed training 96 h			one sample t-test t(6)=1.142	p=0.2969
Figure S1D	Performance of spaced relief training 45 s ITI different from zero	8	yes	one-sample t-test t(7)=5.191	p=0.0013
	Performance of spaced relief training 90 s ITI different from zero			one-sample t-test t(7)=2.425	p=0.0411
	Performance of spaced relief training 135 s ITI different from zero			one-sample t-test t(7)=1.597	p=0.1543
Figure S1E	LTM (CS+ vs CS-) performance with varying ITI	12	yes	F[3,44]=1.228	p=0.31
Figure S1F	CS- memory (CS- vs Novel)	11	yes	F[3,40]=1.462	p=0.2393
Figure S2A	LTM performance (CS+ vs CS-); PAM block	14	yes	F[2,39]=7.229	p=0.0021
	R58E02-GAL4; UAS-GtACR1 vs.+; UAS-GtACR1			Tukey's multiple comparisons test	p=0.0370
	R58E02-GAL4; UAS-GtACR1 vs. R58E02-GAL4; +				p=0.0018
	R58E02-GAL4; + vs.+; UAS-GtACR1				p=0.4920
Figure S2B	LTM performance (CS+ vs CS-); PAM block (light controls)	8	yes	F[2,21]=0.2825	p=0.7567
Figure S2C	LTM performance (CS+ vs CS-); PAM block (temperature controls)	6	yes	F[2,15]=1.605	p=0.2335
Figure S2D	CS+ memory (CS+ vs Novel); PAM block (temperature controls)	6	yes	F[2,15]=1.132	p=0.3484
Figure S2E	CS- memory (CS- vs Novel); PAM block (temperature controls)	6	yes	F[2,15]=0.3142	p=0.7351
Figure S2F	LTM performance (CS+ vs CS-); PAM block during the 4th trial	7	yes	F[2,18]=0.9606	p=0.4014
Figure S2G	LTM performance (CS+ vs CS-); PAM block during the 6th trial	8	yes	F[2,21]=0.5720	p=0.5729
Figure S3A	R66C08-GAL4 after spaced training, presynaptic terminals	24	yes	paired t-test t(23)=1.815	p=0.0825
Figure S3B	R66C08-GAL4 after spaced training reverse order, presynaptic terminals	18	yes	paired t-test t(17)=1.464	p=0.1615
Figure S4A	CS+ memory (CS+ vs Novel); γ3, γ3β′1 MBONs block	8	yes	F[2,21]= 0.4088	p=0.6696
Figure S4B	CS- memory (CS- vs Novel); γ3, γ3β′1 MBONs block	7	yes	F[2,18]=7.316	p=0.0047
	+; MB110C-GAL4/UAS-Shi^{ts1} vs.+; UAS-Shi^{ts1}			Tukey's multiple comparisons test	p=0.0042
	+; MB110C-GAL4/UAS-Shi^{ts1} vs. +; MB110C-GAL4				p=0.0431
	+; MB110C-GAL4 vs.+; UAS-Shi^{ts1}				p=0.5292
Figure S5A	LTM performance (CS+ vs CS-); PPL1-DAN block	7	yes	F[2,18]=6.134	p=0.0093
	MB504B-GAL4; UAS-Shi^{ts1} vs.+; UAS-Shi^{ts1}			Tukey's multiple comparisons test	p=0.0290
	MB504B-GAL4; UAS-Shi^{ts1} vs. MB504B-GAL4; +				p=0.0129
	MB504B-GAL4; + vs.+; UAS-Shi^{ts1}				p=0.9216
Figure S5B	CS+ memory (CS+ vs Novel); PPL1-DAN block	7	yes	F[2,18]=10.99	p=0.0008
	MB504B-GAL4; UAS-Shi^{ts1} vs.+; UAS-Shi^{ts1}			Tukey's multiple comparisons test	p=0.0237
	MB504B-GAL4; UAS-Shi^{ts1} vs. MB504B-GAL4; +				p=0.0006
	MB504B-GAL4; + vs.+; UAS-Shi^{ts1}				p=0.2257
Figure S5C	CS- memory (CS- vs Novel); PAM-γ3 DAN block	7	yes	F[2,18]=5.393	p=0.0146
	MB441B-GAL4; UAS-Shi^{ts1} vs.+; UAS-Shi^{ts1}			Tukey's multiple comparisons test	p=0.0233
	MB441B-GAL4; UAS-Shi^{ts1} vs. MB441B-GAL4; +				p=0.0334
	MB441B-GAL4; + vs.+; UAS-Shi^{ts1}				p=0.9833
Figure S5D	LTM performance (CS+ vs CS-); PAM-β′2mp DAN block	7	yes	F[2,18]=5.855	p=0.0110
	MB056B-GAL4; UAS-Shi^{ts1} vs.+; UAS-Shi^{ts1}			Tukey's multiple comparisons test	p=0.0463
	MB056B-GAL4; UAS-Shi^{ts1} vs. MB056B-GAL4; +				p=0.0122
	MB056B-GAL4; + vs.+; UAS-Shi^{ts1}				p=0.8000
Figure S5E	CS- memory (CS- vs Novel); PAM-β′2mp DAN block	7	yes	F[2,18]=5.395	p=0.0146
	MB056B-GAL4; UAS-Shi^{ts1} vs.+; UAS-Shi^{ts1}			Tukey's multiple comparisons test	p=0.0455
	MB056B-GAL4; UAS-Shi^{ts1} vs. MB056B-GAL4; +				p=0.0184
	MB056B-GAL4; + vs.+; UAS-Shi^{ts1}				p=0.9000
Figure S5F	LTM performance (CS+ vs CS-); PAM-β′1 DAN block	7	yes	F[2,18]=8.944	p=0.002
	MB025B-GAL4; UAS-Shi^{ts1} vs.+; UAS-Shi^{ts1}			Tukey's multiple comparisons test	p=0.0239
	MB025B-GAL4; UAS-Shi^{ts1} vs. MB025B-GAL4; +				p=0.0018
	MB025B-GAL4; + vs.+; UAS-Shi^{ts1}				p=0.4695
Figure S5G	CS- memory (CS- vs Novel); PAM-β′1 DAN block	7	yes	F[2,18]=5	p=0.0188
	MB025B-GAL4; UAS-Shi^{ts1} vs.+; UAS-Shi^{ts1}			Tukey's multiple comparisons test	p=0.0261
	MB025B-GAL4; UAS-Shi^{ts1} vs. MB025B-GAL4; +				p=0.0472
	MB025B-GAL4; + vs.+; UAS-Shi^{ts1}				p=0.9546
Figure S5H	LTM performance (CS+ vs CS-); PPL1-DAN block (temperature controls)	7	yes	F[2,18]=1.925	p=0.1747
Figure S5I	CS+ memory (CS+ vs Novel); PPL1-DAN block (temperature controls)	7	yes	F[2,18]=0.8590	p=0.4402
Figure S5J	CS- memory (CS- vs Novel); PAM-γ3 DAN block (temperature controls)	7	yes	F[2,18]=0.02939	p=0.9711

Figure S5K	LTM performance (CS+ vs CS-); PAM-β′2mp DAN block (temperature controls)	7	yes	F[2,18]=0.2763	p=0.7617
Figure S5L	CS- memory (CS- vs Novel); PAM-β′2mp DAN block (temperature controls)	7	yes	F[2,18]=1.053	p=0.3693
Figure S5M	LTM performance (CS+ vs CS-); PAM-β′1 DAN block (temperature controls)	7	Yes	F[2,18]=1.031	p=0.3769
Figure S5N	CS- memory (CS- vs Novel); PAM-β′1 DAN block (temperature controls)	7	yes	F[2,18]=1.064	p=0.3658
Figure S6A	MB441B-GAL4 after spaced training CS+ vs CS- response 1st trial	20	yes	paired t-test(19)=3.219	p=0.0033
	MB441B-GAL4 after spaced training CS+ vs CS- response 3rd trial	20	Yes	paired t-test(19)=3.485	p=0.0017
	MB441B-GAL4 after spaced training CS+ vs CS- response 6th trial	20	yes	paired t-test(19)=3.805	p=0.0007
Figure S6C	MB441B-GAL4 after reversed spaced training CS+ vs CS- response 1st trial	20	yes	paired t-test(19)=4.443	p=0.0004
	MB441B-GAL4 after reversed spaced training CS+ vs CS- response 3rd trial	20	Yes	paired t-test(19)=8.27	p<0.0001
	MB441B-GAL4 after reversed spaced training CS+ vs CS- response 6th trial	20	yes	paired t-test(19)=3.567	p=0.0026
Figure S6E	MB025B-GAL4 after spaced training CS+ vs CS- response 1st trial	20	yes	paired t-test(19)=1.365	p=0.1855
	MB025B-GAL4 after spaced training CS+ vs CS- response 3rd trial	20	Yes	paired t-test(19)=2.161	p=0.0414
	MB025B-GAL4 after spaced training CS+ vs CS- response 6th trial	20	yes	paired t-test(19)=2.172	p=0.0404
Figure S6G	MB025B-GAL4 after reversed spaced training CS+ vs CS- response 1st trial	20	yes	paired t-test(19)=0.0972	p=0.9246
	MB025B-GAL4 after reversed spaced training CS+ vs CS- response 3rd trial	20	Yes	paired t-test(19)=0.0975	p=0.9242
	MB025B-GAL4 after reversed spaced training CS+ vs CS- response 6th trial	20	yes	paired t-test(19)=0.2381	p=0.8166
Figure S6I	MB056B-GAL4 after spaced training CS+ vs CS- response 1st trial	20	no	Wilcoxon matched-pairs signed rank W(19)=24	p=0.8242
	MB056B-GAL4 after spaced training CS+ vs CS- response 3rd trial	20	no	Wilcoxon matched-pairs signed rank W(19)=17	p=0.8968
	MB056B-GAL4 after spaced training CS+ vs CS- response 6th trial	20	no	Wilcoxon matched-pairs signed rank W(19)=202	p=0.0437
Figure S6K	MB056B-GAL4 after reversed spaced training CS+ vs CS- response 1st trial	20	no	Wilcoxon matched-pairs signed rank W(19)=-86	p=0.4319
	MB056B-GAL4 after reversed spaced training CS+ vs CS- response 3rd trial	20	no	Wilcoxon matched-pairs signed rank W(19)=20	p=0.8609
	MB056B-GAL4 after reversed spaced training CS+ vs CS- response 6th trial	20	no	Wilcoxon matched-pairs signed rank W(19)=184	p=0.0872

Table S2. Odor and shock acuity tests. Related to Figures 1, 2, 5 and S1, S2, S5.

Genotype	Odor Acuity						Shock Acuity	
	23°C			32°C			23°C	32°C
	MCH	OCT	IAA	MCH	OCT	IAA		
WT	-0.32±0.06	-0.30±0.06	-0.35±0.05	N.A.			-0.65±0.03	0.60±0.07
rad	-0.34±0.08	-0.31±0.08	-0.37±0.09	N.A.			-0.65±0.04	0.60±0.03
CXM+	-0.33±0.08	-0.30±0.06	-0.30±0.05	N.A.			-0.69±0.03	0.60±0.04
CXM-	-0.36±0.07	-0.37±0.03	-0.28±0.03	N.A.			-0.65±0.03	0.56±0.04
UAS-*Shi*^{ts1}/+	-0.32±0.07	-0.26±0.03	-0.28±0.04	-0.51±0.04	-0.49±0.03	-0.39±0.05	-0.65±0.06	0.61±0.04
UAS-*Shi*$^{ts1;}$ R58E02	-0.28±0.04	-0.27±0.03	-0.31±0.04	-0.40±0.06	-0.49±0.03	-0.45±0.04	-0.65±0.03	-0.62±0.03
R58E02/+	-0.32±0.04	-0.25±0.03	-0.30±0.04	-0.43±0.08	-0.40±0.05	-0.39±0.04	-0.60±0.03	-0.6±0.04
UAS-GtACR1;R58E02	-0.31±0.07	-0.39±0.06	-0.29±0.06	N.A.			-0.68±0.04	N.A.
UAS-GtACR1/+	-0.32±0.05	-0.34±0.04	-0.35±0.08	N.A.			-0.69±0.05	N.A.
UAS-*Shi*^{ts1};MB504B	-0.36±0.03	-0.29±0.04	-0.30±0.04	-0.51±0.03	-0.41±0.07	-0.34±0.04	-0.77±0.05	0.63±0.04
UAS-*Shi*^{ts1};MB056B	-0.33±0.07	-0.24±0.06	-0.36±0.06	-0.44±0.04	-0.45±0.04	-0.40±0.04	-0.78±0.03	0.66±0.02
UAS-*Shi*^{ts1};MB441B	-0.29±0.05	-0.33±0.03	-0.33±0.03	-0.49±0.04	-0.47±0.05	-0.34±0.03	-0.64±0.07	0.55±0.02
UAS-*Shi*^{ts1};MB025B	-0.36±0.03	-0.37±0.06	-0.29±0.04	-0.41±0.05	-0.51±0.04	-0.39±0.06	-0.72±0.05	0.7±0.02
MB504B/+	-0.30±0.05	-0.29±0.02	-0.34±0.06	-0.46±0.09	-0.54±0.05	-0.31±0.07	-0.68±0.03	0.7±0.04
MB056B/+	-0.32±0.04	-0.27±0.05	-0.31±0.06	-0.40±0.04	-0.54±0.05	-0.37±0.04	-0.68±0.04	0.59±0.04
MB441B/+	-0.30±0.02	-0.29±0.02	-0.37±0.05	-0.47±0.04	-0.47±0.05	-0.35±0.06	-0.63±0.05	0.56±0.04
MB025B/+	-0.31±0.03	-0.34±0.05	-0.30±0.03	-0.44±0.06	-0.49±0.03	-0.36±0.04	-0.67±0.05	0.61±0.05

There are no statistical differences between the relevant groups. Data is displayed Mean ± SEM and n=7 for all groups.

N.A. not applicable
